# Quantitative proteomic analysis of human serum using tandem mass tags to predict cardiovascular risks in patients with psoriasis

**DOI:** 10.1038/s41598-023-30103-2

**Published:** 2023-02-17

**Authors:** Na Young Kim, Ji Hyun Back, Jong Hwan Shin, Mi-Jung Ji, Su Jin Lee, Yae Eun Park, Hyun-Mee Park, Man Bock Gu, Ji Eun Lee, Jeong Eun Kim

**Affiliations:** 1 Department of Dermatology, Hanyang University College of Medicine, 222 Wangsimni-ro, Seongdong-gu, Seoul, 04763 Republic of Korea; 2 Department of Biotechnology, College of Life Sciences and Biotechnology, Korea University, Seoul, 02841 Republic of Korea; 3 Chemical & Biological Integrative Research Center, Biomedical Research Institute, Korea Institute of Science and Technology, Seoul, 02792 Republic of Korea; 4 Advanced Analysis and Data Center, Korea Institute of Science and Technology, Seoul, 02792 Republic of Korea; 5 Hanyang Institute of Bioscience and Biotechnology, Hanyang University, Seoul, 04763 Republic of Korea

**Keywords:** Skin diseases, Biotechnology, Biomarkers, Risk factors

## Abstract

Although biomarker candidates associated with psoriasis have been suggested, those for predicting the risk of cardiovascular disease (CVD) early in patients with psoriasis are lacking. We aimed to identify candidate biomarkers that can predict the occurrence of CVD in psoriasis patients. We pursued quantitative proteomic analysis of serum samples composed of three groups: psoriasis patients with and those without CVD risk factors, and healthy controls. Age/Sex-matched serum samples were selected and labeled with 16-plex tandem mass tag (TMT) and analyzed using liquid chromatography-mass spectrometry and subsequent verification with ELISA. Of the 184 proteins that showed statistical significance (*P*-value < 0.05) among the three groups according to TMT-based quantitative analysis, 98 proteins showed significant differences (> 2.0-fold) between the psoriasis groups with and without CVD risk factors. Verification by ELISA revealed that caldesmon (CALD1), myeloid cell nuclear differentiation antigen (MNDA), and zyxin (ZYX) levels were significantly increased in the psoriasis group with CVD risk factors. Further network analysis identified pathways including integrin signaling, which could be related to platelet aggregation, and actin cytoskeleton signaling. Three novel candidates (MNDA, ZYX, and CALD1) could be potential biomarkers for predicting CVD risks in psoriasis patients. We expect these biomarker candidates can be used to predict CVD risk in psoriasis patients in clinical settings although further studies including large validation are needed.

## Introduction

Psoriasis is a chronic and inflammatory disorder that mainly affects the skin, with an estimated prevalence of 2–4% worldwide^1,2^. In recent years, psoriasis is now more than beyond skin and has important systemic manifestations such as psoriatic arthritis, metabolic syndrome, and cardiovascular disease^3^.

Epidemiologic studies have shown a pathophysiological correlation between psoriasis and cardiovascular disease (CVD) based on the concept of systemic inflammation^1,4,5^. These studies suggest that possible mechanisms such as insulin resistance, oxidative stress, and common inflammatory cytokines, can be used to explain the link between psoriasis and CVD^1,3,6^. Risk factors of CVD including hypertension, diabetes mellitus, dyslipidemia, smoking, alcohol intake, and obesity are found more frequently in patients with psoriasis than in healthy people^7^. As the prevalence of CVD risk factors is increasing, life expectancy has been shown to decrease in patients with psoriasis, emphasizing that a more accurate assessment of CVD risks can allow for making earlier treatments and prevention of clinical events and death in patients with psoriasis.

During the last few decades, proteomics technology has been extensively employed to investigate several aspects of psoriasis and CVD. Additionally, it has been widely used to expand the pathogenesis and pathophysiology of diseases, and discovering novel biomarkers for diagnosis, prognosis, and evaluation of treatment efficacy^8^. Although biomarker candidates associated with psoriasis or CVD, respectively, have been suggested^2,9,10^, they are limited in reflecting CVD occurrence in patients with psoriasis. Thus, the discovery of novel protein biomarkers specific to CVD occurrence in patients with psoriasis is expected to improve the risk assessment for CVD in these patients. Therefore, this study aimed to identify novel biomarker candidates for predicting the risk of CVD in patients with psoriasis and tandem mass tag (TMT)-based quantitative proteomic analysis allowing for simultaneous quantification of multiple samples was pursued using serum samples composed of psoriasis groups with and without CVD risk factors and healthy controls.

## Methods

### Ethics statement

The study protocol was approved by the Institutional Review Board of Hanyang University Hospital (HYU 2018-05-017), and all of the research was carried out in accordance with the applicable rules and regulations. All participants provided written informed consent prior to enrollment.

### Participants and serum samples

The study participants were divided into three groups (Group A, B, and C) recruited from the dermatology outpatients at the Hanyang University Hospital. Group A (n = 7) included psoriasis patients with CVD risk factors. The patients had been diagnosed with psoriasis and had at least one underlying condition including hypertension, diabetes mellitus, and dyslipidemia. Group B (n = 4) included psoriasis patients who had no underlying conditions as described above. Group C (n = 3) included healthy patients as the control group. We excluded participants with underlying diseases other than CVD risk factors that could affect the systemic inflammatory responses. We collected serum samples from the participants and selected a total of 14 age- and sex-matched individuals for proteomic analysis, as shown in Table 1. Blood samples from each participant was collected in a plain tube (6 mL Vacutainer (BD, Franklin Lakes, NJ, USA)). The blood samples were allowed to clot for 15–30 min at room temperature. Finally, serum from peripheral blood was prepared by centrifugation (1300×*g*, 10 min) and stored in a deep freezer (at < − 70 ℃) until analysis.

**Table 1:** The demographic data of participants in three groups.

Characteristics	Group A	Group B	Group C	*P*-value
Number of patients (n)	7	4	3	–
Mean age (years, mean ± SD)	38.14 ± 5.11	36.00 ± 9.80	37.00 ± 8.89	0.19
Sex				
Male (n, %)	7 (100%)	4 (100%)	3 (100%)	–
Female (n, %)	0 (0%)	0 (0%)	0 (0%)	–
PASI score (mean ± SD)	14.41 ± 6.81	9.9 ± 8.33	N/C	0.23
BMI (mean ± SD)	27.44 ± 5.41	22.00 ± 1.83	23.44 ± 2.22	0.16
Disease duration of psoriasis (months, mean ± SD)	106.29 ± 77.90	99.00 ± 59.09	N/C	0.99

(Group A: psoriasis patients with CVD risk factors, Group B: psoriasis patients without CVD risk factors, Group C: healthy control).

SD, standard deviation; PASI, psoriasis area severity index; BMI, body mass index.

### Immunoaffinity depletion of top 14 high-abundance proteins and in-solution tryptic digestion

A protease inhibitor cocktail (Roche Diagnostics, Mannheim, Germany) was added to the 14 serum samples from the psoriasis with and without CVD risk factors (n = 7 and n = 4, respectively) and healthy control (n = 3) groups that were selected for proteomic analysis. The protein concentrations of each serum samples were determined using a bicinchoninic acid (BCA) protein assay kit (Thermo Fisher Scientific, Bremen, Germany). Each serum sample was then depleted of the top 14 high-abundance proteins (albumin, alpha-1 acid glycoprotein, alpha-1 antitrypsin, alpha-2 macroglobulin, apolipoprotein A-I, apolipoprotein A-II, complement C3, fibrinogen, haptoglobin, immunoglobulin A, immunoglobulin G, immunoglobulin M, serotransferrin, and transthyretin) using a human multiple affinity removal system (MARS)-14 column (4.6 × 50 mm, Agilent, Santa Clara, CA, USA) according to the manufacturer’s instructions. 1100 HPLC system (Agilent, Santa Clara, CA, USA) with a fraction collector was used to collect the flow-through fraction. The flow-through fraction not bound to the MARS-14 column in each serum sample was concentrated using Amicon Ultracel-3 centrifugal filter devices (3 kDa cutoff, Millipore, Billerica, MA, USA) and the protein concentrations of serum samples after the removal of the top 14 high-abundance proteins were determined using the BCA protein assay kit. The concentrated samples were resuspended in 8 M urea buffer/25 mM ammonium bicarbonate, and subsequently reduced and alkylated using 5 mM tris (2-carboxyethyl) phosphine hydrochloride and 10 mM iodoacetamide, respectively. Afterwards, 25 mM ammonium bicarbonate was added to the serum samples to decrease the urea concentration to less than 1 M and digested with lysyl endopeptidaseR (Lys-C, Fujifilm Wako Pure Chemical Corporation, Osaka, Japan) in an enzyme/substrate ratio of 1 mAU Lys-C per 50 μg of total protein at 25 °C for 2 h^11^. Trypsin (Promega, Madison, WI, USA) was then added to the samples at a protease/substrate ratio of 1:50 (wt/wt) and incubated at 37 °C overnight. Digested peptide samples were acidified and desalted using a Sep-Pak tC18 cartridge (Waters Corporation, Milford, MA, USA). The desalted peptide samples were then dried in a miVAC vacuum concentrator (Genevac Ltd., Ipswich, UK).

### Tandem mass tags (TMT) labeling

The desalted peptides from each serum samples were resuspended in 30 μL of 100 mM triethyl ammonium bicarbonate (TEAB) and the peptide concentrations were determined using a quantitative colorimetric peptide assay kit (Thermo Fisher Scientific, Rockford, IL, USA). After TMTpro 16plex label reagents (0.5 mg per vial, Thermo Fisher Scientific, Rockford, IL, USA, VI306840 (lot number)) were resuspended in 60 μL of anhydrous acetonitrile (ACN), 30 μL of each label reagent was added to 30 μg of each peptide samples for labeling. In addition to the 14 serum samples used for proteomic analysis, two serum samples obtained from patients diagnosed as psoriatic arthritis were also labeled because a total of 16 TMT label reagents were available; however, the two serum samples were not used for TMT-based quantitative analysis (labels used 126: psoriasis patient 1 with CVD risk factors; 127N: psoriasis patient 2 with CVD risk factors; 127C: psoriasis patient 3 with CVD risk factors; 128N: psoriasis patient 1 without CVD risk factors; 128C: psoriasis patient 2 without CVD risk factors; 129N: psoriasis patient 3 without CVD risk factors; 129C: psoriasis patient 4 without CVD risk factors; 130N: control 1; 130C: control 2; 131N: control 3; 131C: psoriasis patient 4 with CVD risk factors; 132N: psoriasis patient 5 with CVD risk factors; 132C: psoriasis patient 6 with CVD risk factors; 133N: patient 1 with psoriatic arthritis; 133C: patient 2 with psoriatic arthritis; 134N: psoriasis patient 7 with CVD risk factors). After the labeling reaction was made for 60 min at room temperature, 5 μL of 5% hydroxylamine in 100 mM TEAB was added to each peptide sample and incubated for 15 min to quench the labeling reactions. Equal amounts of TMT-labeled samples were combined and desalted using a Sep-Pak tC18 cartridge and dried in an miVAC vacuum concentrator.

### Basic pH reversed‑phase liquid chromatography

The TMT-labeled peptide samples were resuspended in 10 mM ammonium formate and the peptide concentrations were determined using a quantitative colorimetric peptide assay kit. Afterwards, 292 μg of each peptide sample was loaded onto an X-Bridge peptide BEH C18 column (4.6 mm i.d. × 250 mm length; pore size 130 Å; particle size 3.5 μM, Waters Corporation, Milford, MA, USA) and fractionated by basic pH reversed-phase liquid chromatography using an Agilent 1290 Infinity liquid chromatography (LC) system (Agilent Technology, Santa Clara, CA). The peptides were separated at a flow rate of 0.5 mL/min with the following gradient conditions: 0 min 100% buffer A (10 mM ammonium formate, pH 10) and 0% buffer B (10 mM ammonium formate (pH 10) in 90% acetonitrile); 0–10 min, 0–5% B; 10–48.5 min, 5–40% B; 48.5–62.5 min, 40–70% B; 62.5–72.5 min, 70% B; 72.5–82.5 min, 70–5% B; and 82.5–92.5 min, 5% B. Fractionation was pursued by collecting 96 wells (1 well/0.8 min, Restek corporation, Bellefonte, PA, USA) during the chromatographic run (from 10 to 82.5 min). The resultant 96 fractions were pooled into 24 concatenated fractions using the following rule: A set of arithmetic sequences with a common difference of 24 was pooled into one concatenated fraction; for example, fractions 1, 25, 49, and 63 were pooled into the first concatenated fraction. The 24 peptide fractions were dried and subsequently resuspended in 12.16 μL of 0.4% acetic acid.

### Liquid chromatography and tandem mass spectrometry (LC–MS/MS) analysis

3 µg of the fractionated peptide sample was injected onto a trap column (2 cm × 75 µm i.d., 100 Å, 3 µm) and separated on a reversed-phase Acclaim PepMap RSLC C18 column (50 cm × 75 µm i.d., 100 Å, 2 µm) using an UltiMate 3000 RSLCnano System (Thermo Fisher Scientific, Bremen, Germany). The column temperature was constantly set to 50 ℃ by using a column heater. The operating flow rate was 300 nL/min with the following gradient conditions: 0 min 95% buffer A (100% water with 0.1% formic acid) and 5% buffer B (100% acetonitrile with 0.1% formic acid); 0–4 min, 5% B; 4–13 min, 5–10% B; 13–150 min, 10–25% B; 150–155 min, 25–28% B; 155–160 min, 28–40% B; 160–165 min, 40–80% B; 165–170 min, 80% B; 170–170.1 min, 80–5% B; and 170.1–180 min, 5–0% B. The nano UHPLC system was coupled to an Orbitrap Eclipse Tribrid mass spectrometer (Thermo Fisher Scientific, Bremen, Germany). MS1 data were collected using an Orbitrap (120,000 resolution; scan range of 350–2000 m*/z*; maximum injection time 50 ms; AGC 4 × 10^5^). Determined charge states between 2 and 6 were required for sequencing and a 30 s dynamic exclusion window was used. Data-dependent ‘top 20’ MS2 scans were performed in 0.5 Da isolation window of the ion trap with collision-induced dissociation (CID) fragmentation (NCE 35%; maximum injection time 35 ms; AGC 1 × 10^4^). MS3 quantification scans were performed using the multi-notch MS3-based TMT method (ten synchronous precursor selection (SPS) ions; resolution, 50,000; NCE, 55% for higher-energy collisional dissociation (HCD); the maximum injection time was 130 ms; AGC 1.5 × 10^5^)^12^.

### Data processing for protein identification and quantification

MS raw files were searched against the SwissProt human database (May 2020) with 20,329 entries using Proteome Discoverer software (version 2.4, Thermo Fisher Scientific). The search criteria were set to a mass tolerance of 10 ppm for MS data and 0.6 Da for MS/MS data with fixed modifications of carbamidomethylation of cysteine (+ 57.021 Da) and TMT on lysine residues and peptide N termini (+ 304.207 Da) and variable modification of methionine oxidation (+ 15.995 Da). The false discovery rate (FDR) was set at 0.01 for the identification of peptides and proteins. All the proteins were identified by two or more unique peptides. Reporter ion isotopic distribution correction was pursued in order to correct the factors for natural carbon isotopes and incomplete isotope incorporation. The reporter ion isotopic distributions obtained from the product data sheet (VI306840) of the TMTpro reagent were used as isotope correction factors in TMTpro 16plex method template in Proteome Discoverer software (version 2.4). Reporter ion quantification was performed with a mass tolerance of 20 ppm. While signal-to-noise ratio values of reporter ions were used for quantification of peptides, only spectra with an average reporter signal-to-noise ratio threshold of ≥ 10 across 16 TMTpro 16plex channels were considered for the quantification. The signal-to-noise ratio values of each reporter ion channel were summed across all quantified proteins and normalized so that the summed signal-to-noise ratio values of each channel could be equal across all 16 channels, which were called normalized abundance values. The normalized abundance values were first log-transformed and missing values were then replaced using values computed from the normal distribution with a width of 0.3 and a downshift of 1.8. Proteins exhibiting statistical significances among the psoriasis groups with and without CVD risk factors and a healthy control group were detected using one-way analysis of variance (ANOVA) comparison of the log_2_(normalized abundance) values using Perseus software (1.6.14.0)^13^. The proteins with a *P*-value < 0.05, from ANOVA analysis, were then further subjected to a Scheffe’s post-hoc analysis to identify those proteins that exhibited statistically significant changes between the psoriasis group with CVD risk factors and the psoriasis group without CVD risk factors (*P*-value < 0.05). For hierarchical clustering of the proteins with statistically significant changes (*P*-value < 0.05, > 2.0-fold) between psoriasis groups with and without CVD risk factors, log_2_(normalized abundance) values were first normalized using z-score and then clustering of both columns and rows was pursued based on Euclidean distance using the average linkage method using Perseus (1.6.14.0).

### Gene Ontology (GO) enrichment analysis and ingenuity pathway analysis (IPA)

To identify functional implications and signaling pathways involved in proteins showing significant increases in psoriatic patients with CVD risk factors, we carried out Gene Ontology (GO) and Ingenuity Pathway Analysis (IPA). GO functional classifications of the proteins showing statistically significant increases (*P*-value < 0.05, > 2.0-fold) in the psoriasis group with CVD risk factors were analyzed using the DAVID software (http://david.abcc.ncifcrf.gov) to identify GO terms that were significantly enriched in the proteins. Additionally, IPA software (data version 76765844; QIAGEN, Redwood City, CA, USA) was used to analyze molecular and cellular functions, canonical pathways, and the associated network functions of the proteins exhibiting statistically significant increases in the psoriasis group with CVD risk factors.

### Enzyme-linked immunosorbent assay (ELISA)

From the TMT-based proteomic data, caldesmon (CALD1), LIM and SH3 domain protein 1 (LASP1), myeloid cell nuclear differentiation antigen (MNDA), and zyxin (ZYX) showed statistically significant increases in the psoriasis group with CVD risk factors in compared to the group without CVD risk factors, and were selected for verification of ELISA using 42 serum samples consisting of 22 psoriasis samples from participants with CVD risk factors and 20 psoriasis samples from participants without CVD risk factors (see Supplementary Table S1 online for the validation cohort). The concentrations of CALD1 (Novus Biologicals, Centennial, CO, USA), LASP1 (Cusabio, Houston, TX, USA), MNDA (Mybiosource, Inc., San Diego, CA, USA), and ZYX (Cusabio, Houston, TX, USA) were determined using ELISA kits. The serum samples were diluted 1:3, 1:3, 1:2, and 1:2 for measurement of concentrations of CALD1, LASP1, MNDA, and ZYX, respectively (see Supplementary Fig. S1 online for standard curves for the measurement of concentrations of each protein).

### Statistical analysis

Data were analyzed with using SPSS software version 25 (SPSS, Chicago, IL, USA). Differences in demographic data and baseline clinical findings among the three groups were assessed using the Kruskal–Wallis test. The results of proteomic analysis using LC/MS were compared using one-way ANOVA and post-hoc analysis was performed using pairwise multiple comparison procedures (Scheffe’s method) to evaluate differences between the psoriasis groups with and without CVD risk factors. Subsequent verification with ELISA were analyzed using the independent t-test and Mann–Whitney U-test.

## Results

### Baseline characteristics

Fourteen age- and sex-matched individuals were included in this proteomic analysis. The demographic and descriptive variables are shown in Table 1. All groups consisted of male participants with a mean age of 37.5 ± 6.43 years. The mean of Psoriasis Area Severity Index (PASI) score was not significantly different among the three groups (*P*-value = 0.23). In addition, the differences in body mass index (BMI) and disease duration among the three groups were not statistically significant (*P*-value = 0.16, *P*-value = 0.99, respectively).

### Proteomic analysis of TMT-labeled samples

**Figure 1:**
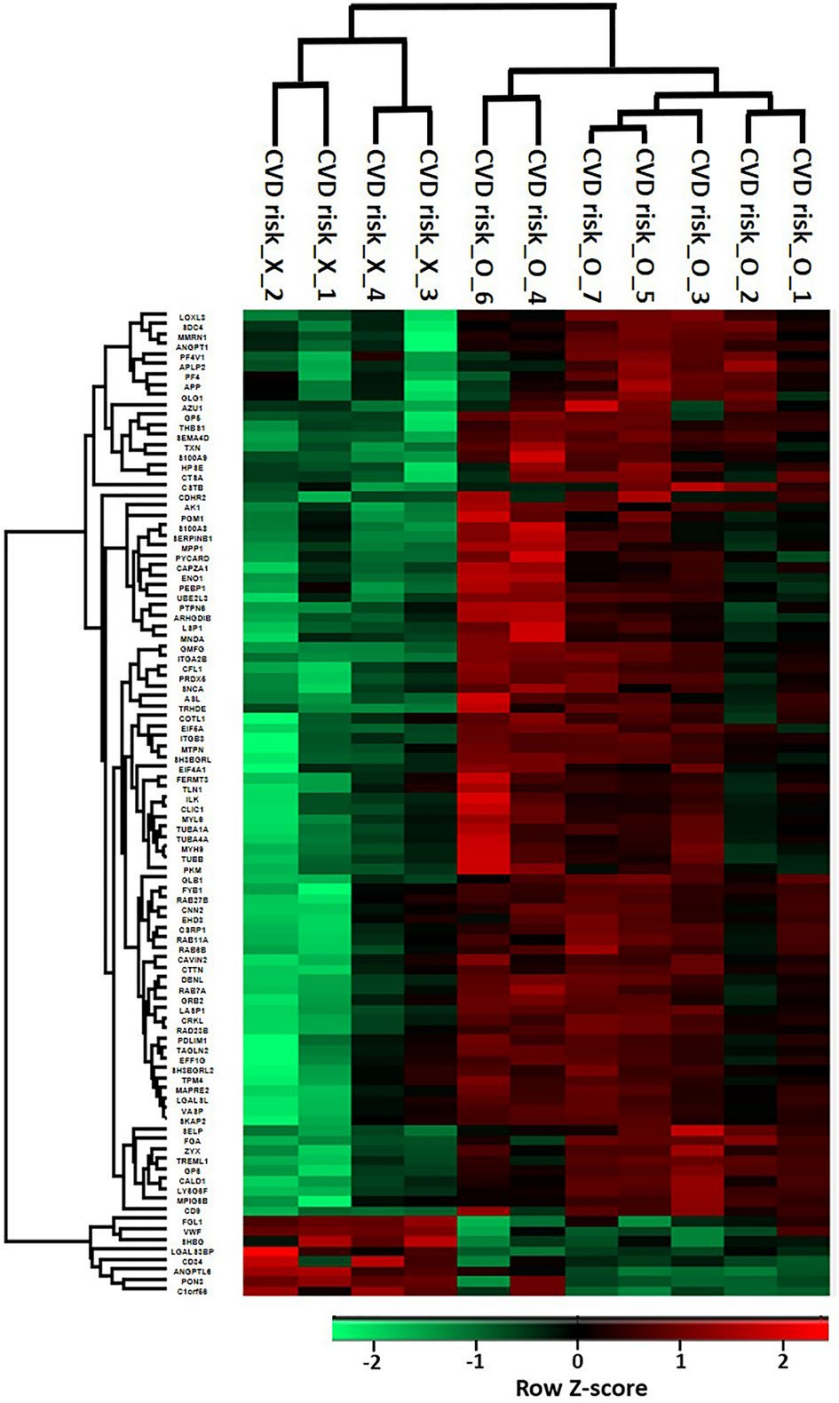
Heat map exhibiting hierarchical clustering of 98 proteins with statistically significant changes (> 2.0-fold, P-value < 0.05 from ANOVA followed by Scheffe’s post-hoc analysis) between psoriasis groups with and without cardio vascular disease (CVD) risk factors. The rows represent each protein and the columns show four and seven biological replicates of psoriasis serum samples without and with CVD risk factors, respectively. Hierarchical clustering of the 98 proteins was performed in Perseus software (1.6.14.0) on log-transformed normalized signal-to-noise ratio values after z-score normalization of the data.

From the MS analysis of the TMT-labeled serum samples, a total of 1079 proteins were identified by two or more unique peptides and 1041 proteins of the identified proteins were quantifiable (see Supplementary Table S2 online). From one-way ANOVA analysis of the three groups consisting of the psoriasis groups with and without CVD risk factors and the healthy control group, 184 proteins were found to show statistical significance (*P*-value < 0.05) (see Supplementary Fig. S2online for hierarchical clustering analysis of the 184 proteins). Then, the 184 proteins with a *P*-value < 0.05 from the ANOVA analysis were subsequently subjected to Scheffe’s post-hoc analyses, respectively, for a psoriasis group with CVD risk factors and a healthy control group, a psoriasis group without CVD risk factors and a healthy control group, and the psoriasis groups with and without CVD risk factors to evaluate differences between the two groups (see Supplementary Table S3 online). From the results, 73, 3, and 143 proteins were, respectively, statistically significant (*P*-value < 0.05) from pairwise multiple comparisons between the psoriasis group with CVD risk factors and the healthy control group, between the psoriasis group without CVD risk factors and the healthy control group, and between the psoriasis groups with and without CVD risk factors. These results showed some similarity with the heat map exhibiting hierarchical clustering analysis of the 184 proteins as seen in Supplementary Fig. S2 because the psoriasis group without CVD risk factors and the healthy group did not reveal distinct expression patterns from the hierarchical clustering analysis. Since we originally attempted to identify those proteins that exhibited statistically significant changes between psoriasis groups with and without CVD risk factors, we primarily focused on the psoriasis groups with and without CVD risk factors for further analysis. While 98 proteins showed statistically significant changes (> 2.0-fold, *P*-value < 0.05) between the two groups, a hierarchical clustering analysis of the 98 proteins showed that most of the proteins with statistically significant changes increased in psoriasis group with CVD risk factors in comparison to that without CVD risk factors (Fig. 1). Actually, 90 proteins among 98 proteins showed significant increases in psoriasis group with CVD risk factors compared to that without CVD risk factors (Table 2).

**Table 2:** Proteins showing statistically significant increases (> 2.0-fold, *P*-value < 0.05) from TMT-labeling based quantitative analyses of psoriasis groups with and without CVD risk factors.

Accession no.	Protein description	Gene symbol	Scheffe *P*-value^a^	Ratio (CVD risk_O/CVD risk_X)^b^
Q86YW5	Trem-like transcript 1 protein	TREML1	7.968E−04	12.328
P41218	Myeloid cell nuclear differentiation antigen	MNDA	4.203E−02	8.715
Q5SQ64	Lymphocyte antigen 6 complex locus protein G6f	LY6G6F	8.427E−04	8.342
Q9HCN6	Platelet glycoprotein VI	GP6	2.081E−04	7.608
O95866	Megakaryocyte and platelet inhibitory receptor G6b	C6orf25; MPIG6B	1.483E−02	7.588
Q15942	Zyxin	ZYX	6.255E−04	7.239
Q13418	Integrin-linked protein kinase	ILK	2.972E−02	7.061
P60842	Eukaryotic initiation factor 4A-I	EIF4A1	3.786E−02	5.462
P60660	Myosin light polypeptide 6	MYL6	1.358E−02	5.211
Q15555	Microtubule-associated protein RP/EB family member 2	MAPRE2	5.198E−03	5.175
O00151	PDZ and LIM domain protein 1	PDLIM1	2.892E−02	5.098
Q05682	Caldesmon	CALD1	2.690E−03	5.096
Q71U36	Tubulin alpha-1A chain	TUBA1A	1.041E−02	4.920
Q99439	Calponin-2	CNN2	9.596E−03	4.873
Q14247	Src substrate cortactin	CTTN	6.885E−03	4.806
P23528	Cofilin-1	CFL1	2.882E−03	4.660
P68366	Tubulin alpha-4A chain	TUBA4A	1.688E−02	4.603
P46109	Crk-like protein	CRKL	1.306E−03	4.521
P37802	Transgelin-2	TAGLN2	2.489E−02	4.379
P21291	Cysteine and glycine-rich protein 1	CSRP1	7.556E−03	4.378
Q14847	LIM and SH3 domain protein 1	LASP1	9.592E−04	4.264
Q9UJU6	Drebrin-like protein	DBNL	3.671E−03	4.247
O15117	FYN-binding protein 1	FYB; FYB1	1.371E−02	4.239
O00194	Ras-related protein Rab-27B	RAB27B	3.156E−02	4.198
Q9BYE9	Cadherin-related family member 2	CDHR2	1.429E−02	4.113
P50552	Vasodilator-stimulated phosphoprotein	VASP	1.569E−02	4.113
Q9ULZ3	Apoptosis-associated speck-like protein containing a CARD	PYCARD	2.569E−02	3.914
P35579	Myosin-9	MYH9	3.000E−02	3.902
P52907	F-actin-capping protein subunit alpha-1	CAPZA1	1.523E−02	3.896
P33241	Lymphocyte-specific protein 1	LSP1	1.614E−02	3.835
Q9UKU6	Thyrotropin-releasing hormone-degrading ectoenzyme	TRHDE	2.576E−03	3.835
O00299	Chloride intracellular channel protein 1	CLIC1	2.839E−02	3.811
P51149	Ras-related protein Rab-7a	RAB7A	6.280E−03	3.719
Q00013	55 kDa erythrocyte membrane protein	MPP1	4.040E−02	3.716
P58546	Myotrophin	MTPN	1.021E−02	3.636
Q86UX7	Fermitin family homolog 3	FERMT3	4.073E−02	3.598
P05109	Protein S100-A8	S100A8	3.568E−02	3.583
O95810	Caveolae-associated protein 2	SDPR; CAVIN2	2.629E−02	3.552
P26641	Elongation factor 1-gamma	EEF1G	3.258E−02	3.549
P16109	P-selectin	SELP	1.228E−03	3.549
P21926	CD9 antigen	CD9	6.960E−04	3.548
O60234	Glia maturation factor gamma	GMFG	1.077E−04	3.442
P05106	Integrin beta-3	ITGB3	2.263E−03	3.430
P62491	Ras-related protein Rab-11A	RAB11A	7.720E−03	3.429
Q3ZCW2	Galectin-related protein	LGALSL	1.791E−02	3.366
Q9Y490	Talin-1	TLN1	4.487E−02	3.365
P06702	Protein S100-A9	S100A9	5.082E−03	3.362
P29350	Tyrosine-protein phosphatase non-receptor type 6	PTPN6	3.993E-02	3.274
P06733	Alpha-enolase	ENO1	2.772E-02	3.273
P63241	Eukaryotic translation initiation factor 5A-1	EIF5A	1.259E-02	3.079
P07437	Tubulin beta chain	TUBB	3.953E-02	3.053
P67936	Tropomyosin alpha-4 chain	TPM4	4.901E-02	3.046
P30044	Peroxiredoxin-5, mitochondrial	PRDX5	1.445E-03	3.009
Q9NZN3	EH domain-containing protein 3	EHD3	3.863E−02	2.985
P62993	Growth factor receptor-bound protein 2	GRB2	2.234E−02	2.934
P30740	Leukocyte elastase inhibitor	SERPINB1	1.135E−02	2.901
P04424	Argininosuccinate lyase	ASL	3.284E−03	2.898
P04080	Cystatin-B	CSTB	2.804E−02	2.867
P02671	Fibrinogen alpha chain	FGA	1.683E−03	2.864
P07996	Thrombospondin-1	THBS1	4.174E−04	2.851
P52566	Rho GDP-dissociation inhibitor 2	ARHGDIB	4.755E−02	2.812
Q92854	Semaphorin-4D	SEMA4D	9.741E−06	2.702
P37840	Alpha-synuclein	SNCA	4.651E−02	2.689
P10599	Thioredoxin	TXN	4.125E−03	2.655
P16278	Beta-galactosidase	GLB1	6.025E−04	2.647
P31431	Syndecan-4	SDC4	2.594E−03	2.641
P08514	Integrin alpha-IIb	ITGA2B	1.734E−04	2.624
P40197	Platelet glycoprotein V	GP5	2.550E−03	2.602
Q9Y251	Heparanase	HPSE	1.041E−02	2.596
Q14019	Coactosin-like protein	COTL1	4.029E−02	2.583
P54727	UV excision repair protein RAD23 homolog B	RAD23B	7.377E−03	2.565
Q9UJC5	SH3 domain-binding glutamic acid-rich-like protein 2	SH3BGRL2	4.271E−02	2.539
P00568	Adenylate kinase isoenzyme 1	AK1	1.584E−02	2.537
P30086	Phosphatidylethanolamine-binding protein 1	PEBP1	3.199E−02	2.461
P36871	Phosphoglucomutase-1	PGM1	1.386E−02	2.429
P10720	Platelet factor 4 variant	PF4V1	2.392E−02	2.425
O75368	SH3 domain-binding glutamic acid-rich-like protein	SH3BGRL	1.663E−02	2.335
Q06481	Amyloid-like protein 2	APLP2	4.829E−03	2.333
P14618	Pyruvate kinase PKM	PKM	1.573E−02	2.326
Q9NRW1	Ras-related protein Rab-6B	RAB6B	4.226E−03	2.316
P58215	Lysyl oxidase homolog 3	LOXL3	3.526E−03	2.263
P68036	Ubiquitin-conjugating enzyme E2 L3	UBE2L3	1.655E−02	2.256
P02776	Platelet factor 4	PF4	4.996E−02	2.125
Q13201	Multimerin-1	MMRN1	1.455E−02	2.108
Q15389	Angiopoietin-1	ANGPT1	1.387E−02	2.102
P10619	Lysosomal protective protein	CTSA	1.060E−02	2.093
O75563	Src kinase-associated phosphoprotein 2	SKAP2	1.482E−02	2.087
P20160	Azurocidin	AZU1	3.807E−02	2.048
P05067	Amyloid-beta precursor protein	APP	4.441E−02	2.046
Q92896	Golgi apparatus protein 1	GLG1	4.198E−02	2.030

^a^Scheffe *P*-values were obtained from post-hoc analysis performed by pairwise multiple comparison procedures (Scheffe's method) between psoriasis groups with and without CVD risk factors.

^b^Ratio represents relative abundance ratios based on normalized signal-to-noise value between psoriasis groups with and without CVD risk factors.

### Over-represented functional processes and ingenuity network analysis

**Figure 2:**
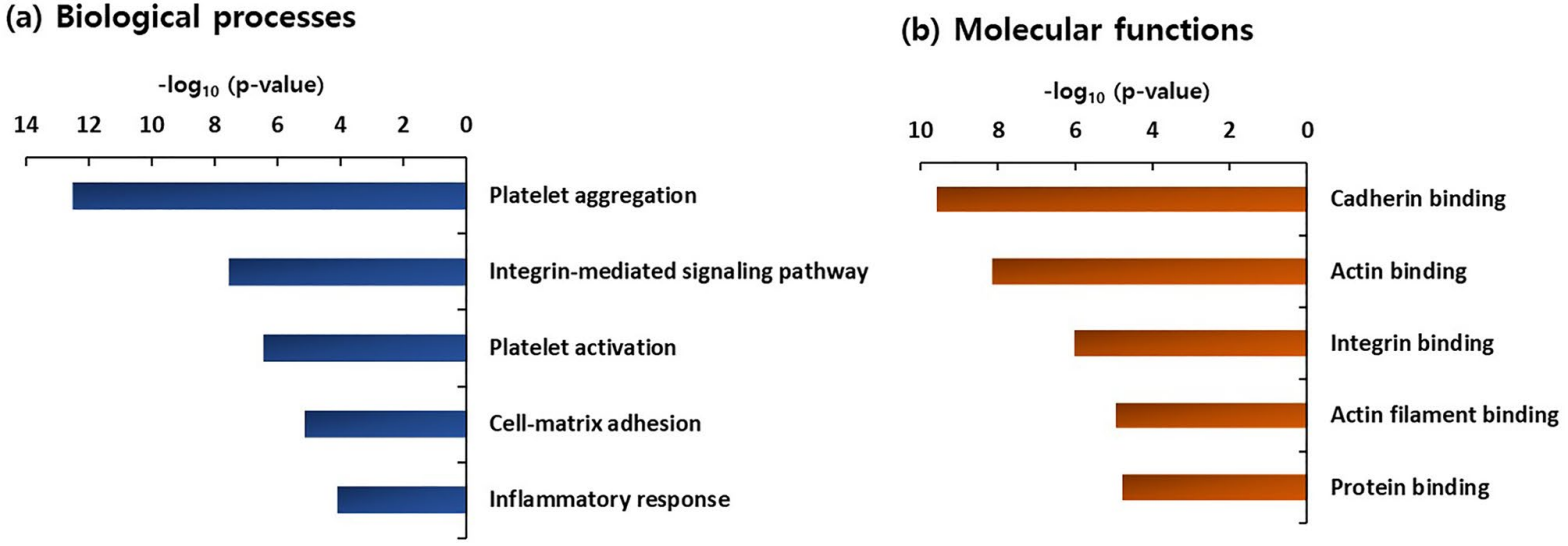
Top 5 biological process (BP) and molecular function (MF) terms (**a** and **b**), respectively, over-represented in proteins showing statistically significant increases (*P*-value < 0.05, > 2.0-fold) by Gene Enrichment (GO) analysis.

**Figure 3:**
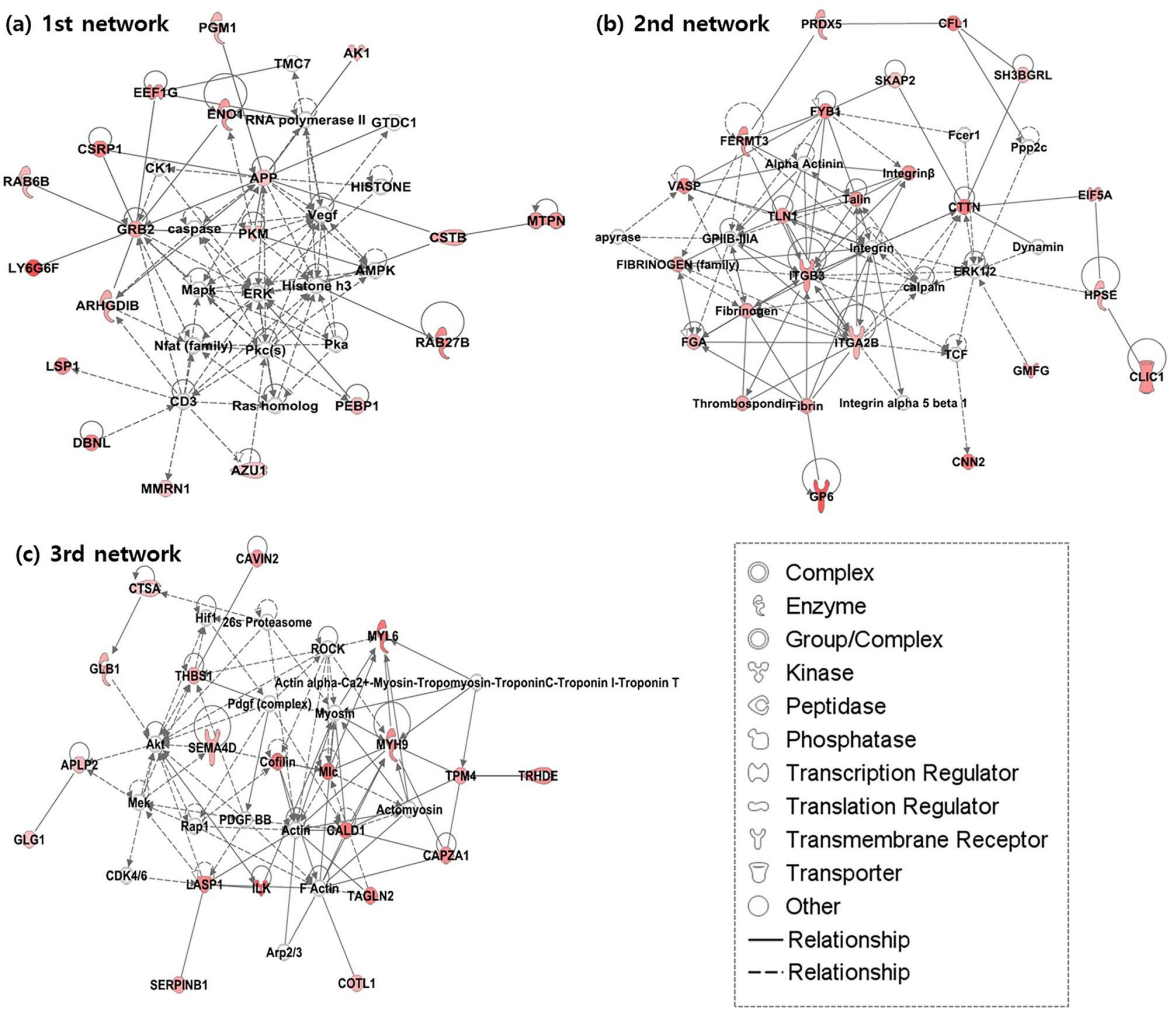
Top three interaction networks generated by ingenuity pathway analysis (IPA) software (data version 76765844) are displayed, respectively, in (**a**)–(**c**). Proteins showing statistically significant increases (P-value < 0.05, > 2.0-fold) in psoriasis patients with CVD risk factors are in red. ERK, integrin/ERK1/2, and actin are, respectively, are seen as the major hubs in each network. Straight lines indicate direct interactions, whereas dashed lines indicate indirect ones.

From the GO enrichment analysis of the 90 proteins showing statistically significant increases (> 2.0-fold) in the psoriasis group with CVD risk factors, platelet aggregation and platelet activation terms were enriched in the top five biological process terms (Fig. 2A). In addition, actin binding and integrin-binding terms were found in the top five molecular functions (Fig. 2B). In addition to GO enrichment analysis, integrin signaling was also found to be the top canonical pathway from the IPA analysis of the 90 proteins exhibiting significant increases in the psoriasis patients with CVD risk factors (see Supplementary Table S4 online). Actin cytoskeleton signaling that was included as one of the top five canonical pathways for the 90 proteins from the IPA analysis has been reported to be implicated in inflammation in cardiovascular disease^14^. The top five molecular and cellular functions in order of significance were; ‘cell to cell signaling and interaction, ‘cellular movement’, ‘cellular assembly and organization’, ‘cellular function and maintenance’, and ‘cellular morphology’. The top five canonical pathways were ‘integrin signaling, ‘remodeling of epithelial adherens junctions’, ‘GP6 signaling pathway’, ‘axonal guidance signaling’, and ‘actin cytoskeleton signaling’. The top three networks, respectively, containing 19, 18, and 18 proteins contained extracellular signal-regulated kinase (ERK), integrin/ERK1/2, and actin as the major hubs (Fig. 3).

### Selection of biomarker candidates and subsequent verification using ELISA

To identify potential biomarker candidates for further verification, we used both mass spectrometric data and existing evidence in the current literature on biomarkers related to CVD. Regarding proteins that showed statistically significant increases (Scheffe’s test *P*-value < 0.05, > 2.0-fold) in the psoriasis group with CVD risk factors compared to those without CVD risk factors, we attempted to select novel biomarker candidates, which were studied in relation to CVD but their roles in psoriasis were not previously reported from literature search. Finally, four biomarker candidates including CALD1, LASP1, MNDA, and ZYX were selected for verification based on previous literature reviews^15–25^, the availability of suitable ELISA kits, and available sample volumes.

**Figure 4:**
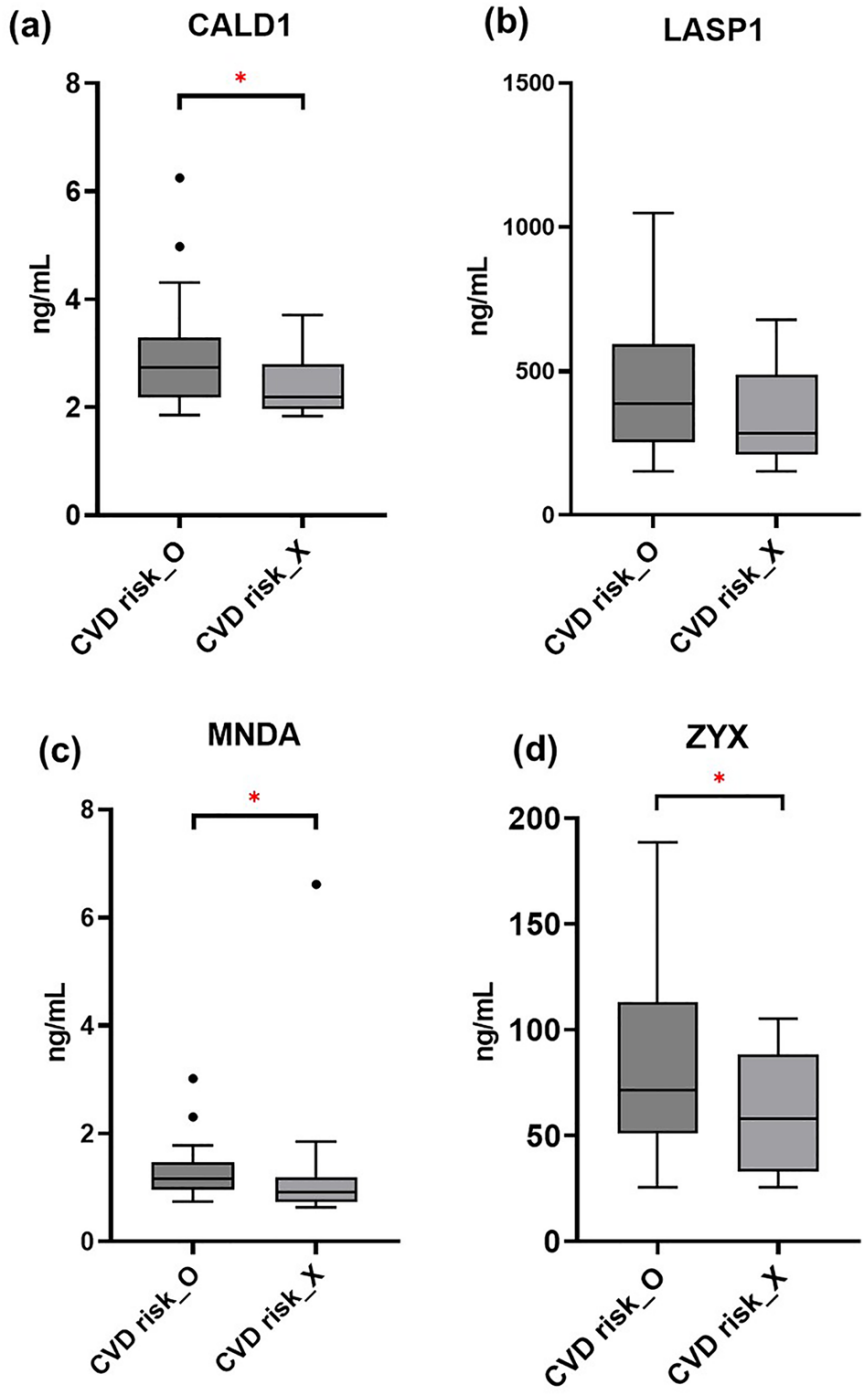
Boxplots of biomarker candidates verified by ELISA. The order of groups is identical in all charts as indicated on the bottom (CVD risk_O: psoriasis groups with CVD risk factors, CVD risk_X: psoriasis groups without CVD risk factors). The groups that show significant differences of the respective protein are indicated by brackets. Statistical significance was defined as *P < 0.05. (**a**) CALD1: Caldesmon, (**b**) LASP1: LIM and SH3 domain protein, (**c**) MNDA: Myeloid cell nuclear differentiation antigen, (**d**) ZYX: Zyxin.

While all of four proteins (CALD1, LASP1, MNDA, and ZYX) were differentially expressed between psoriatic patients with and without CVD risk factors (Fig. 4 and Supplementary Table S5 online for raw data of ELISA), the concentrations of CALD1, MNDA, and ZYX were significantly higher in the psoriasis group with CVD risk factors than in the psoriasis group without CVD risk factors (*P*-value < 0.05).

## Discussion

Psoriasis is a common immune-mediated inflammatory disorder with an increased risk of CVD, which implies that chronic cutaneous inflammation expands to the systemic comorbidities^26,27^. In fact, systemic inflammatory processes could cause endothelial dysfunction, increased arterial thickness, vascular inflammation, and insulin resistance, which could lead to CVD in patients with psoriasis^28–31^. Early studies of biomarkers for psoriasis focused on underlying pathophysiologic processes and various circulating biomarkers for diagnosis, severity, and treatment response of in patients with psoriasis have been reported^2,6,8,32^. For example, serum levels of inflammatory and pro-inflammatory markers including C-reactive protein (CRP), complement 3 (C3), platelet P-selectin, tumor necrosis factor (TNF)-α, interleukin (IL)-6, IL-8, IL-12, and IL-18 were increased in patients with psoriasis^9,10^. Furthermore, S100 proteins such as S100A7, S100A8, and S100A9 are involved in abnormal keratinocyte differentiation and are upregulated in psoriatic skin lesion as well as in the blood^2,33^. Moreover, cytoskeletal and Ca2+-binding proteins such as thymosin-γ4, profilin-1, and talin-1 were upregulated in patients with psoriasis^34^. From our TMT-labeled quantitative analysis, 56 proteins were found to show statistically significant increases (P-value < 0.05, > 2.0-fold) in psoriasis group with/without CVD risk factors in comparison to healthy control group (see Supplementary Table S3 online). Among these proteins, well-known circulating markers for psoriasis such as P-selectin (P16109), S100A8 (P05109), S100A9 (P06702), and talin-1 (Q9Y490) were found to be upregulated in patients with psoriasis compared to healthy control.

Various potential biomarkers for CVD have been suggested with regard to myocardial injury or stress (cardiac troponins and natriuretic peptides), plaque instability (lipoprotein-associated phospholipase A2, matrix metalloproteinase-9), and adhesion molecules (VCAM-1, ICAM-1, and E-selectin)^35–39^. Also, general inflammatory biomarkers such as CRP, TNF-α, IL-6, and IL-8, systemic stress, and calcium homeostasis were regarded as important pathophysiological processes in CVD^40,41^. Additionally, many biomarkers associated with oxidative stress including isoprostanes, lipoprotein-associated phospholipase A2, coenzyme Q10, and glutathione, have been found^40^. However, there have been no practical biomarkers available to early predict the risk of CVD in psoriasis patients in real clinical settings. This study investigated a set of serum proteins related to CVD risk factors to identify those associated with subclinical cardiovascular events in the setting of psoriasis. Oxidative stress related proteins such as Peroxiredoxin-5 (P30044) and thioredoxin (P10599) were more upregulated in psoriasis with CVD risk factors than in psoriasis without CVD risk factors in the current study. The effects of oxidative stress and elevated antioxidant levels have already been reported in CVD-like atherosclerosis^42,43^.

Through TMT as one of isobaric labeling tags-based quantitative proteomic analysis and subsequent verification using ELISA, we especially identified three novel biomarkers associated with CVD risks in patients with psoriasis; CALD1, ZYX, and MNDA. All showed statistically significant differences between psoriatic patients with and those without CVD risk factors in ELISA experiments. In addition, GO and IPA analyses of the proteins showing statistically significant increases in psoriasis group with CVD risk factors in comparison to that without CVD risk factors revealed that biological processes of platelet aggregation/activation and molecular processes of integrin and actin bindings were overrepresented. Furthermore, integrin and actin were as the major hubs of signaling network analysis using IPA. As a group of cytoskeletal proteins, the main function of CALD1 and ZYX is actually a vital mediator in many biological pathways from cell to cell adhesion and migration to cellular proliferation^44^. Specifically, CALD1 is a calcium-calmodulin and actin-binding protein found most abundantly in smooth muscles, which is involved in stabilizing actin filaments, cellular proliferation, and migration^15,45^. Some studies have shown the possible relationship between CALD1 expression in tissue or blood and CVD^15–17^. ZYX is a focal adhesion protein that regulates actin cytoskeleton and shows intracellular distribution where actin filaments are highly concentrated^46^. As a stretch-sensitive regulator, ZYX is known to be stimulated by prolonged hypertension, an important predisposing factor in the development of endothelial dysfunctions, which subsequently induces a broad range of inflammatory responses^23,24^. Regarding molecular functions, ZYX transmits external forces like hypertension to focal adhesion proteins, enhances actin assembly, and subsequently interacts with integrins, which are associated with multiple adaptor proteins in various signaling pathways^25^. The functions and the associations of the cytoskeletal proteins with CVD risks in psoriatic patients have not been clarified; however, those proteins could be associated with CVD risks in psoriatic patients based on our current findings that molecular functions and interaction networks related to actin/integrin binding were overrepresented in psoriatic patients with CVD risk factors. In particular, CALD1 was closely related to actin, the main hub of the third network in our IPA results, which corresponds to the main function of CALD1.

Besides, platelet was included in the main biological processes associated with CVD in patients with psoriasis by GO analysis. In both psoriasis and CVD, platelets are actually known to play a critical role in the pathogenesis. In coronary artery diseases, increased platelet activation or aggregation is known to be associated with the risk factors including smoking, hypertension, and hypercholesterolemia^47–49^. Furthermore, platelets also act as immune cells that initiate and regulate inflammatory processes, which could be related to increased risks of cardiovascular events^48^. The hemostatic imbalance as well as immunologic factors are important in prothrombotic circumstances, which might be induced by platelet activation or aggregation^50^. While integrin binding was included in the top five molecular function terms, integrin signaling was found to be related to cardiovascular diseases such as atherosclerosis via MAP kinase signaling and cardiac hypertrophy via Rho GTPase signaling^51,52^. Specifically, a dimer of integrin alpha-IIb (P08514)/integrin beta-3 (P05106), which showed significant increases (> 2.0-fold) in psoriasis patient with CVD risk factors in our current study (Table 2), is known to be activated through binding of fibrinogen followed by platelet aggregation^51,53^. ERK signaling was also reported to be associated with the cardiac hypertrophy^54^ and the relationship ERK and MAP kinase was identified in the first network in our IPA results.

MNDA has been proposed to be a transcriptional factor in myeloid cells, and it plays a role in both differentiation or apoptosis of myeloid cells and inflammatory pathways^55,56^. In particular, MNDA is related to the neutrophil death program and proper elimination of neutrophils is required for the resolution of inflammation^55^. In psoriatic skin, abundant neutrophils including Munro’s microabscesses are important histopathologic hallmarks. Circulating neutrophils are collected in skin lesions and activate subsequent inflammatory responses such as reactive oxygen species (ROS) production, T cell stimulations, and angiogenesis^57^. Also, the neutrophil-to-lymphocyte ratio was increased in patients with psoriasis^58^. Similar to psoriasis, neutrophils have been recognized as important contributors in inflammatory processes to inflammatory processes in CVD. Previous studies suggested there was an association between neutrophils and the risk of CVD^21,22,59–61^. Moreover, it was reported that neutrophils are involved in the early atherosclerotic plaque formation^62^. Taken together, neutrophils may be involved in early damage of both the epidermis and the endothelium. Also, a prior proteomic study on the spondyloarthropathy implicated MNDA as a meaningful biomarker to detect inflammatory responses earlier than other markers such as IL-6 and CRP^63^. Elevated MNDA upregulation could implicate the development of inflammatory processes in various disorders where neutrophils especially play an important role.

Our study has some limitations. First, we had a limited sample size, which could preclude the generalizability of our results. While we attempted to include a similar number of serum samples in the three groups, there was a limit to collecting an equal number of samples when we selected the samples for proteomic analysis. Despite these limitations, our study has several strengths. First, we attempted to divide the patients with psoriasis into two groups according to the strict diagnosis of cardiovascular risk factors and attempted to analyze the differences in protein expression levels of each serum samples. Second, isobaric labeling mass tags-based quantitative analysis in the current study allowed for simultaneous quantification of several samples due to multiplexing capability, resulting in reduction of instrument time and inter-sample variation compared to a label-free quantification method^64,65^. Third, further verification using ELISA for biomarker candidates specific to psoriatic patients with CVD risk factors was performed.

So far, there are no independent biomarkers to predict cardiovascular risk in patients with psoriasis. In this study, we found that differences in the expression of CALD1, ZYX, and MNDA between psoriasis with cardiovascular risk factors and those without cardiovascular risk factors were significant. We expect these biomarker candidates can be used to predict CVD risk in psoriasis in future clinical settings. The results require further external validation and may currently be viewed as a hypothesis requiring future research. The next steps are to confirm the value of the identified proteins in a large size of subjects.

In conclusion, we found that CALD1, ZYX, and MNDA showed significant discrimination between psoriatic patients with and without CVD risk factors. Several pathways have been associated with CVD risk factors in patients with psoriasis, including ‘integrin signaling’ and ‘actin cytoskeleton signaling’. The role of integrin signaling could be related to actin cytoskeleton signaling via ZYX, with regard to mechanoreceptor and subsequent gene regulation. Additionally, platelet aggregation with integrin signaling may be associated with prothrombotic events or inflammatory processes in CVD. Further studies should be performed to evaluate using larger sizes of subjects whether these biomarkers will have practical clinical applications for the early diagnosis and management of psoriasis in patients with CVD risk factors.

## Supplementary Information

Supplementary Information.

## Data Availability

The data analyzed in the current study are available from the corresponding author on reasonable request. The mass spectrometry data have been deposited to the Proteome Xchange Consortium via the PRIDE^66^. Partner repository with the dataset identifier PXD039645.

## References

[1] Hu SC, Lan CE (2017). Psoriasis and cardiovascular comorbidities: Focusing on severe vascular events, cardiovascular risk factors and implications for treatment. Int. J. Mol. Sci..

[2] Reindl J (2016). Proteomic biomarkers for psoriasis and psoriasis arthritis. J. Proteom..

[3] Boehncke WH, Boehncke S, Tobin AM, Kirby B (2011). The 'psoriatic march': A concept of how severe psoriasis may drive cardiovascular comorbidity. Exp. Dermatol..

[4] Davidovici BB (2010). Psoriasis and systemic inflammatory diseases: Potential mechanistic links between skin disease and co-morbid conditions. J. Investig. Dermatol..

[5] Furue M, Tsuji G, Chiba T, Kadono T (2017). Cardiovascular and metabolic diseases comorbid with psoriasis: Beyond the skin. Intern. Med..

[6] Chularojanamontri L, Charoenpipatsin N, Silpa-Archa N, Wongpraparut C, Thongboonkerd V (2019). Proteomics in psoriasis. Int. J. Mol. Sci.

[7] Altobelli E (2009). Risk factors of hypertension, diabetes and obesity in Italian psoriasis patients: A survey on socio-demographic characteristics, smoking habits and alcohol consumption. Eur. J. Dermatol..

[8] Jiang S, Hinchliffe TE, Wu T (2015). Biomarkers of an autoimmune skin disease-psoriasis. Genom. Proteom. Bioinform..

[9] Rocha-Pereira P (2004). The inflammatory response in mild and in severe psoriasis. Br. J. Dermatol..

[10] Garbaraviciene J (2010). Platelet P-selectin reflects a state of cutaneous inflammation: Possible application to monitor treatment efficacy in psoriasis. Exp. Dermatol..

[11] Mertins P (2018). Reproducible workflow for multiplexed deep-scale proteome and phosphoproteome analysis of tumor tissues by liquid chromatography-mass spectrometry. Nat. Protoc..

[12] McAlister GC (2014). MultiNotch MS3 enables accurate, sensitive, and multiplexed detection of differential expression across cancer cell line proteomes. Anal. Chem..

[13] Tyanova S (2016). The Perseus computational platform for comprehensive analysis of (prote)omics data. Nat. Methods.

[14] Thomas T, Advani A (2006). Inflammation in cardiovascular disease and regulation of the actin cytoskeleton in inflammatory cells: The actin cytoskeleton as a target. Cardiovasc. Hematol. Agents Med. Chem..

[15] Hai CM (2008). Caldesmon as a therapeutic target for proliferative vascular diseases. Mini Rev. Med. Chem..

[16] Glukhova MA (1988). Modulation of human aorta smooth muscle cell phenotype: A study of muscle-specific variants of vinculin, caldesmon, and actin expression. Proc. Natl. Acad. Sci. USA.

[17] Touyz RM (2018). Vascular smooth muscle contraction in hypertension. Cardiovasc. Res..

[18] Hong M (2022). Network pharmacology and experimental analysis to reveal the mechanism of Dan-Shen-Yin against endothelial to mesenchymal transition in atherosclerosis. Front. Pharmacol..

[19] Šatrauskienė A, Navickas R, Laucevičius A, Huber HJ (2017). Identifying differential miR and gene consensus patterns in peripheral blood of patients with cardiovascular diseases from literature data. BMC Cardiovasc. Disord..

[20] Lim SH, Lee J, Han MJ (2020). Comprehensive analysis of the cardiac proteome in a rat model of myocardial ischemia-reperfusion using a TMT-based quantitative proteomic strategy. Proteome Sci..

[21] Lin JD (2019). Single-cell analysis of fate-mapped macrophages reveals heterogeneity, including stem-like properties, during atherosclerosis progression and regression. JCI Insight.

[22] Briggs RC, Atkinson JB, Miranda RN (2005). Variable expression of human myeloid specific nuclear antigen MNDA in monocyte lineage cells in atherosclerosis. J. Cell Biochem..

[23] Ghosh S (2015). Loss of the mechanotransducer zyxin promotes a synthetic phenotype of vascular smooth muscle cells. J. Am. Heart Assoc..

[24] Wojtowicz A (2010). Zyxin mediation of stretch-induced gene expression in human endothelial cells. Circ. Res..

[25] Wang YX, Wang DY, Guo YC, Guo J (2019). Zyxin: A mechanotransductor to regulate gene expression. Eur. Rev. Med. Pharmacol. Sci..

[26] Frank O, Nestle M, Daniel H, Kaplan M, Barker J (2009). Mechanisms of disease: Psoriasis. N. Engl. J. Med..

[27] Rapp SR, Feldman SR, Exum ML, Fleischer AB, Reboussin DM (1999). Psoriasis causes as much disability as other major medical diseases. J. Am. Acad. Dermatol..

[28] Balci DD (2009). Increased carotid artery intima-media thickness and impaired endothelial function in psoriasis. J. Eur. Acad. Dermatol. Venereol..

[29] Hjuler KF (2017). Increased global arterial and subcutaneous adipose tissue inflammation in patients with moderate-to-severe psoriasis. Br. J. Dermatol..

[30] Armstrong EJ, Harskamp CT, Armstrong AW (2013). Psoriasis and major adverse cardiovascular events: A systematic review and meta-analysis of observational studies. J. Am. Heart Assoc..

[31] Boehncke S (2007). Psoriasis patients show signs of insulin resistance. Br. J. Dermatol..

[32] Pourani MR, Abdollahimajd F, Zargari O, Shahidi-Dadras M (2022). Soluble biomarkers for diagnosis, monitoring, and therapeutic response assessment in psoriasis. J. Dermatolog. Treat..

[33] Rodriguez-Cerdeira C (2019). Biomarkers of inflammation in obesity-psoriatic patients. Mediators Inflamm..

[34] Plavina T, Hincapie M, Wakshull E, Subramanyam M, Hancock WS (2008). Increased plasma concentrations of cytoskeletal and Ca2+-binding proteins and their peptides in psoriasis patients. Clin. Chem..

[35] Kvisvik B (2017). High-sensitivity troponin T vs I in acute coronary syndrome: Prediction of significant coronary lesions and long-term prognosis. Clin. Chem..

[36] Packard CJ (2000). Lipoprotein-associated phospholipase A2 as an independent predictor of coronary heart disease. N. Engl. J. Med..

[37] Khan SQ (2008). Plasma N-terminal B-Type natriuretic peptide as an indicator of long-term survival after acute myocardial infarction: Comparison with plasma midregional pro-atrial natriuretic peptide: The LAMP (Leicester Acute Myocardial Infarction Peptide) study. J. Am. Coll. Cardiol..

[38] Jefferis BJ (2010). Prospective study of matrix metalloproteinase-9 and risk of myocardial infarction and stroke in older men and women. Atherosclerosis.

[39] Keller T (2009). Sensitive troponin I assay in early diagnosis of acute myocardial infarction. N. Engl. J. Med.

[40] Stoner L (2013). Inflammatory biomarkers for predicting cardiovascular disease. Clin. Biochem..

[41] Lyngbakken MN, Myhre PL, Rosjo H, Omland T (2019). Novel biomarkers of cardiovascular disease: Applications in clinical practice. Crit. Rev. Clin. Lab. Sci..

[42] Khosravi M, Poursaleh A, Ghasempour G, Farhad S, Najafi M (2019). The effects of oxidative stress on the development of atherosclerosis. Biol. Chem..

[43] Jeong SJ, Park JG, Oh GT (2021). Peroxiredoxins as potential targets for cardiovascular disease. Antioxidants (Basel).

[44] Ogawa E, Sato Y, Minagawa A, Okuyama R (2018). Pathogenesis of psoriasis and development of treatment. J. Dermatol..

[45] Hai CM, Gu Z (2006). Caldesmon phosphorylation in actin cytoskeletal remodeling. Eur. J. Cell Biol..

[46] Drees BE, Andrews KM, Beckerle MC (1999). Molecular dissection of zyxin function reveals its involvement in cell motility. J. Cell Biol..

[47] Gregg D, Goldschmidt-Clermont PJ (2003). Cardiology patient page. Platelets and cardiovascular disease. Circulation.

[48] Corinaldesi G (2011). Platelet activation in cardiovascular disease. Blood.

[49] Lebas H, Yahiaoui K, Martos R, Boulaftali Y (2019). Platelets are at the nexus of vascular diseases. Front. Cardiovasc. Med..

[50] Fan Z, Wang L, Jiang H, Lin Y, Wang Z (2021). Platelet dysfunction and its role in the pathogenesis of psoriasis. Dermatology.

[51] Finney AC, Stokes KY, Pattillo CB, Orr AW (2017). Integrin signaling in atherosclerosis. Cell Mol. Life Sci..

[52] Lal H (2009). Integrins and proximal signaling mechanisms in cardiovascular disease. Front. Biosci..

[53] Clemetson KJ, Clemetson JM (1998). Integrins and cardiovascular disease. Cell Mol. Life Sci..

[54] Gallo S, Vitacolonna A, Bonzano A, Comoglio P, Crepaldi T (2019). ERK: A key player in the pathophysiology of cardiac hypertrophy. Int. J. Mol. Sci..

[55] Milot E, Fotouhi-Ardakani N, Filep JG (2012). Myeloid nuclear differentiation antigen, neutrophil apoptosis and sepsis. Front. Immunol..

[56] Suzuki T (2012). Reconstruction of monocyte transcriptional regulatory network accompanies monocytic functions in human fibroblasts. PLoS ONE.

[57] Chiang CC, Cheng WJ, Korinek M, Lin CY, Hwang TL (2019). Neutrophils in psoriasis. Front. Immunol..

[58] Sen BB (2014). Neutrophil to lymphocyte ratio as a measure of systemic inflammation in psoriasis. Cutan. Ocul. Toxicol..

[59] Haim M, Boyko V, Goldbourt U, Battler A, Behar S (2004). Predictive value of elevated white blood cell count in patients with preexisting coronary heart disease: The Bezafibrate Infarction Prevention Study. Arch. Intern. Med..

[60] Kannel WB, Anderson K, Wilson PW (1992). White blood cell count and cardiovascular disease. Insights from the Framingham study. JAMA.

[61] Ensrud K, Grimm RH (1992). The white blood cell count and risk for coronary heart disease. Am. Heart J..

[62] Quillard T (2015). TLR2 and neutrophils potentiate endothelial stress, apoptosis and detachment: Implications for superficial erosion. Eur. Heart J..

[63] Wang C (2019). LTF, PRTN3, and MNDA in Synovial fluid as promising biomarkers for periprosthetic joint infection: Identification by quadrupole orbital-trap mass spectrometry. J. Bone Joint Surg. Am..

[64] Thompson A (2003). Tandem mass tags: A novel quantification strategy for comparative analysis of complex protein mixtures by MS/MS. Anal. Chem..

[65] Li J (2020). TMTpro reagents: A set of isobaric labeling mass tags enables simultaneous proteome-wide measurements across 16 samples. Nat. Methods.

[66] Perez-Riverol Y, Bai J, BandlaLi C, Hewapathirana S, García-Seisdedos D, Kamatchinathan S, Kundu D, Prakash A, Frericks-Zipper A, Eisenacher M, Walzer M, Wang S, Brazma A, Vizcaíno JA (2022). The PRIDE database resources in 2022: A Hub for mass spectrometry-based proteomics evidences. Nucleic
Acids Res.

