# Supplementary material for: Quantitative proteomic analysis of human serum using tandem mass tags to predict cardiovascular risks in patients with psoriasis
Source: Sci Rep. 2023 Feb 17;13:2869. doi: 10.1038/s41598-023-30103-2 (PMC9938257; doi:10.1038/s41598-023-30103-2)
Supplement: Supplementary file 1 — Supplementary Information. [file 41598_2023_30103_MOESM1_ESM.pdf]

Quantitative proteomic analysis of human serum using tandem mass tags to predict cardiovascular risks in patients with psoriasis

Na Young Kim, Ji Hyun Back, Jong Hwan Shin, Mi-Jung Ji, Su Jin Lee, Yae Eun Park, Hyun-Mee Park, Man Bock Gu, Ji Eun Lee, and Jeong Eun Kim

Supplementary Materials

Supplementary Tables

1) Supplementary Table S1
2) Supplementary Table S2
3) Supplementary Table S3
4) Supplementary Table S4
5) Supplementary Table S5

Supplementary Figures

1) Supplementary Figure S1
2) Supplementary Figure S2

Supplementary Table S1. Demographic data of the two groups for ELISA
(Group A: Psoriasis patients with CVD risk factors, Group B: Psoriasis patients without CVD risk factors)

**Characteristics**		Group A	Group B	*P* -value
Number of patients (n)		22	20	-
Mean age (years, mean±SD)		48.73±16.21	31.25±10.83	0.01
Sex	Male (n, %)	16 (72.73%)	13 (65%)	-
	Female (n, %)	6 (27.27%)	7 (35%)	-
PASI score (mean±SD)		11.32±6.30	8.21±6.06	0.111
BMI (mean±SD)		26.66±3.96	23.69±3.07	0.01
Disease duration of psoriasis (mean±SD)		98.00±23.01	68.70±14.14	0.286

SD: Standard Deviation, PASI: Psoriasis Area Severity Index, BMI: Body Mass Index

Supplementary Table S2. Identified proteins from TMTpro-labeled serum samples

[a] PSM represents peptide spectrum match, which corresponds to tandem MS/MS spectrum matched to identification of a protein.

[b] AAs and [c] MW represent, respectively, amino acids and molecular weight.

[d] CVD risk O represents normalized signal-to-noise ratio values from psoriasis group with CVD risk factors.

[e] CVD risk X represents normalized signal-to-noise ratio values from psoriasis group without CVD risk factors.

[f] Control represents normalized signal-to-noise ratio values from control group.

[g] Quantifiable Proteins mean proteins having signal-to-noise ratio values of reporter ions from TMT labeling.

Accession No.	Protein Description	Gene Symbol	Sum PEP Score	Coverage [%]	# PSMs[a]	# Unique Peptides	# AAs[b]	MW[c] [kDa]	CVD risk O 1[d]	CVD risk O 2[d]	CVD risk O 3[d]	CVD risk O 4[d]	CVD risk O 5[d]	CVD risk O 6[d]	CVD risk O 7[d]	CVD risk X 1[e]	CVD risk X 2[e]	CVD risk X 3[e]	CVD risk X 4[e]	Control 1[f]	Control 2[f]	Control 3[f]	Quantifiable Proteins[g]
Q04446	1,4-alpha-glucan-branching enzyme	GBE1	9.193	4	2	2	702	80.4	169	153	187	306	237	441	191	275	187	155	151	135	184	225	O
P31946	14-3-3 protein beta/alpha	YWHAB	32.656	49	11	5	246	28.1	382	394	906	1792	521	1902	562	563	180	407	279	193	649	412	O
P62258	14-3-3 protein epsilon	YWHAE	25.376	31	9	5	255	29.2	270	469	602	1220	416	1208	475	625	290	372	283	200	708	530	O
Q04917	14-3-3 protein eta	YWHAH	26.94	25	7	3	246	28.2	160	113	276	474	191	768	168	111	18	121	106	86	120	191	O
P61981	14-3-3 protein gamma	YWHAG	27.812	28	8	3	247	28.3	260	288	520	807	348	1162	282	378	129	252	196	163	313	361	O
P27348	14-3-3 protein theta	YWHAQ	27.802	31	8	3	245	27.7	204	285	1161	720	253	991	309	290	181	235	197	102	371	248	O
P63104	14-3-3 protein zeta/delta	YWHAZ	62.086	59	16	10	245	27.7	772	751	1761	3073	986	5455	850	941	212	1141	707	347	844	947	O
P62333	26S proteasome regulatory subunit 10B	PSMC6	5.45	7	2	2	389	44.1															X
P42765	3-ketoacyl-CoA thiolase, mitochondrial	ACAA2	12.8	7	5	5	397	41.9	227	203	416	336	362	233	249	233	240	344	318	349	286	260	O
P25325	3-mercaptopyruvate sulfurtransferase	MPST	5.28	14	2	2	297	33.2	71	109	131	410	94	449	151	230	52	44	31	33	258	75	O
Q9BRK5	45 kDa calcium-binding protein	SDF4	5.937	8	3	3	362	41.8	515	540	548	313	587	247	453	380	388	312	347	351	402	620	O
P08195	4F2 cell-surface antigen heavy chain	SLC3A2	59.338	24	16	12	630	68	1666	2148	1689	2456	1829	2329	2110	3927	2069	2481	2525	2890	1883	3508	O
P32754	4-hydroxyphenylpyruvate dioxygenase	HPD	21.993	20	6	6	393	44.9	467	153	277	621	512	2041	637	380	470	225	252	162	446	562	O
Q96BZ4	5'-3' exonuclease PLD4	PLD4	18.348	10	4	4	506	55.6	129	230	186	129	141	149	126	146	177	216	176	210	122	237	O
Q00013	55 kDa erythrocyte membrane protein	MPP1	8.397	5	2	2	466	52.3	186	116	192	546	200	535	289	112	57	81	68	45	366	211	O
P05387	60S acidic ribosomal protein P2	RPLP2	7.54	27	2	2	115	11.7	205	75	192	453	99	476	78	139	57	94	81	51	267	80	O
P52209	6-phosphogluconate dehydrogenase, decarboxylating	PGD	59.934	36	15	14	483	53.1	624	764	913	3982	967	3101	1027	768	353	484	434	355	1107	614	O
O95336	6-phosphogluconolactonase	PGLS	16.343	33	5	5	258	27.5	219	156	237	742	199	682	293	281	119	179	114	99	338	164	O
P08253	72 kDa type IV collagenase	MMP2	156.583	47	38	23	660	73.8	4999	2673	2268	1998	2610	2233	2146	5326	2333	2918	2591	2808	2416	3855	O
Q76LX8	A disintegrin and metalloproteinase with thrombospondin motifs 13	ADAMTS13	140.709	28	37	29	1427	153.5	2218	2149	1765	1728	2772	2317	2879	2906	3842	3571	3596	3937	2575	3131	O
P22303	Acetylcholinesterase	ACHE	9.463	6	3	3	614	67.8	29	28	34	32	33	64	24	40	35	30	37	34	25	43	O
Q9BWD1	Acetyl-CoA acetyltransferase, cytosolic	ACAT2	16.692	13	5	4	397	41.3	88	83	111	99	111	211	113	89	55	47	44	41	139	107	O
Q13510	Acid ceramidase	ASAH1	11.596	15	4	4	395	44.6	349	278	274	484	476	206	423	206	235	180	252	203	163	137	O
Q92484	Acid sphingomyelinase-like phosphodiesterase 3a	SMPDL3A	10.455	7	4	3	453	51.2	251	354	331	243	226	322	263	269	244	610	702	574	238	809	O
Q92688	Acidic leucine-rich nuclear phosphoprotein 32 family member B	ANP32B	16.402	18	4	4	251	28.8	61	81	172	622	155	392	107	130	18	88	76	40	145	60	O
Q99798	Aconitate hydratase, mitochondrial	ACO2	32.869	13	10	10	780	85.4	844	726	1552	1281	1395	823	1108	919	989	808	1059	1137	1018	1058	O
P68032	Actin, alpha cardiac muscle 1	ACTC1	100.746	43	82	4	377	42	113	87	125	298	120	471	105	132	55	85	89	76	105	105	O
P60709	Actin, cytoplasmic 1	ACTB	152.163	57	92	7	375	41.7	7026	5414	8832	17895	8134	27461	7271	5588	2942	6626	5141	4502	6211	7349	O
P61160	Actin-related protein 2	ACTR2	12.346	8	2	2	394	44.7	36	29	64	197	43	120	43	43	31	36	25	16	36	40	O
O15143	Actin-related protein 2/3 complex subunit 1B	ARPC1B	17.387	17	5	5	372	40.9	99	67	106	630	127	991	101	149	25	93	68	50	107	74	O
O15144	Actin-related protein 2/3 complex subunit 2	ARPC2	15.104	18	6	6	300	34.3	293	248	446	1592	401	1909	335	351	71	261	208	155	296	279	O
P59998	Actin-related protein 2/3 complex subunit 4	ARPC4	15.372	23	3	3	168	19.7	71	53	92	360	88	403	91	77	8	65	63	35	77	60	O
O15511	Actin-related protein 2/3 complex subunit 5	ARPC5	16.54	35	4	4	151	16.3	161	149	165	566	154	772	170	187	93	145	133	109	183	130	O
P61158	Actin-related protein 3	ACTR3	27.339	26	7	7	418	47.3	195	180	296	1422	290	1794	236	339	72	243	160	116	231	209	O
P13798	Acylamino-acid-releasing enzyme	APEH	14.073	5	3	3	732	81.2	210	398	437	2245	286	1402	473	1173	187	183	170	179	1625	426	O
O15204	ADAM DEC1	ADAMDEC1	11.138	5	3	2	470	52.7	161	231	209	126	179	98	146	119	151	163	159	172	102	265	O
Q86TH1	ADAMTS-like protein 2	ADAMTSL2	53.693	23	16	13	951	104.6	1031	1612	1209	552	1358	917	1041	692	1127	1326	1474	1258	679	1968	O
Q6UY14	ADAMTS-like protein 4	ADAMTSL4	53.624	17	13	13	1074	116.5	547	686	802	326	932	458	881	688	661	894	751	859	449	1145	O
Q9NZK5	Adenosine deaminase 2	CECR1; ADA2	46.472	24	15	12	511	58.9	1200	1348	1721	1992	1726	2283	1815	2270	1468	2477	1802	1369	1361	2099	O
P23526	Adenosylhomocysteinase	AHCY	23.068	16	6	6	432	47.7	130	215	245	1031	174	657	238	554	146	142	83	111	601	242	O
P00568	Adenylate kinase isoenzyme 1	AK1	29.129	42	9	7	194	21.6	347	476	534	622	372	864	577	297	189	207	161	209	913	370	O
Q01518	Adenylyl cyclase-associated protein 1	CAP1	32.187	17	7	7	475	51.9	543	415	923	1995	932	3250	1010	258	168	1115	624	176	468	484	O
P48960	Adhesion G protein-coupled receptor E5	CD97; ADGRE5	37.409	9	9	5	835	91.8	624	910	917	533	990	508	999	541	909	1145	985	786	645	1409	O
Q8IZF2	Adhesion G protein-coupled receptor F5	GPR116; ADGRF5	59.754	11	15	11	1346	149.4	1148	1946	1836	1408	1693	1169	1584	1381	1604	1914	1899	1572	962	2430	O
Q9HBW9	Adhesion G protein-coupled receptor L4	ELTD1; ADGRL4	32.886	14	11	8	690	77.8	568	1233	817	882	1046	698	823	956	594	961	1004	680	439	1566	O
Q8IZP9	Adhesion G-protein coupled receptor G2	GPR64; ADGRG2	18.786	5	3	3	1017	111.5	92	176	127	97	189	90	141	159	181	200	170	203	101	209	O
Q86SQ4	Adhesion G-protein coupled receptor G6	GPR126; ADGRG6	26.383	8	7	7	1221	136.6	510	537	630	410	487	441	512	356	500	486	432	519	379	584	O
Q9HDC9	Adipocyte plasma membrane-associated protein	APMAP	55.497	39	20	14	416	46.5	3638	1141	850	1054	2103	276	3828	625	1903	3362	2831	2749	4618	2009	O
Q15848	Adiponectin	ADIPOQ	46.341	21	33	4	244	26.4	1437	1763	1482	603	1032	1083	1660	2027	1900	1513	2502	2821	1039	2522	O
P12235	ADP/ATP translocase 1	SLC25A4	16.736	16	7	2	298	33	793	775	1333	986	1288	564	989	826	1010	914	983	1168	1056	950	O
Q10588	ADP-ribosyl cyclase/cyclic ADP-ribose hydrolase 2	BST1	30.158	28	6	6	318	35.7	367	700	498	200	674	260	542	576	579	599	486	640	453	335	O
P43652	Afamin	AFM	359.526	65	411	48	599	69	28658	50650	58194	38107	65015	44579	46777	31584	44286	36137	42704	42927	34902	52342	O
P16112	Aggrecan core protein	ACAN	13.184	2	2	2	2530	261.2	54	103	107	50	90	46	70	66	109	122	141	157	83	95	O
O00468	Agrin	AGRN	8.742	2	3	3	2068	217.2															X
P24298	Alanine aminotransferase 1	GPT	59.936	35	12	11	496	54.6	815	551	753	521	1925	4151	1370	1407	536	426	643	479	715	1156	O
P02768	Albumin	ALB	727.424	85	1069	72	609	69.3	50847	62785	150884	110112	128863	44489	88387	7012	78099	115809	276934	202395	94277	128247	O
P07327	Alcohol dehydrogenase 1A	ADH1A	12.516	17	5	2	375	39.8	314	119	359	444	759	1286	337	185	497	160	126	119	304	233	O
P14550	Aldo-keto reductase family 1 member A1	AKR1A1	17.157	18	4	4	325	36.6	139	124	216	632	196	672	163	386	121	144	124	106	237	155	O
P05186	Alkaline phosphatase, tissue-nonspecific isozyme	ALPL	30.653	18	8	8	524	57.3	545	458	364	392	488	438	482	460	463	548	576	396	457	752	O
P08319	All-trans-retinol dehydrogenase [NAD(+)] ADH4	ADH4	39.808	27	7	5	380	40.2	247	69	208	189	497	363	226	74	301	85	93	76	165	142	O
P26572	Alpha-1,3-mannosyl-glycoprotein 2-beta-N-acetylglucosaminyltransferase	MGAT1	21.887	14	5	5	445	50.8	384	518	383	391	429	346	577	259	523	435	473	514	457	522	O
Q10469	Alpha-1,6-mannosyl-glycoprotein 2-beta-N-acetylglucosaminyltransferase	MGAT2	5.444	6	2	2	447	51.5	36	66	78	50	32	43	79	79	85	75	82	51	36	75	O
P02763	Alpha-1-acid glycoprotein 1	ORM1	83.238	41	82	6	201	23.5	1551	1911	999	854	1689	661	347	1495	957	740	1210	982	1080	860	O
P19652	Alpha-1-acid glycoprotein 2	ORM2	67.571	43	56	7	201	23.6	1607	2243	1565	599	1078	611	645	1184	1070	698	1644	1283	1357	805	O
P01011	Alpha-1-antichymotrypsin	SERPINA3	425.811	70	724	32	423	47.6	59687	52598	33467	45741	38160	22238	42861	56739	42379	53493	55035	45308	50388	51491	O
P01009	Alpha-1-antitrypsin	SERPINA1	308.845	67	206	31	418	46.7	26995	9114	11482	5016	30974	1054	7207	4548	7885	15070	14465	14746	19368	9998	O
P04217	Alpha-1B-glycoprotein	A1BG	200.032	54	393	23	495	54.2	34362	33036	28695	21561	32886	22529	14515	28945	28008	34469	36244	32110	27186	37084	O
P08697	Alpha-2-antiplasmin	SERPINF2	208.083	48	125	20	491	54.5	12181	10718	10884	10384	9613	12035	10936	14168	14076	10817	10035	11968	12845	13063	O
P02765	Alpha-2-HS-glycoprotein	AHSG	197.279	64	465	20	367	39.3	22033	35595	41933	27387	36006	19510	30382	28015	37330	34126	39675	34928	27273	37946	O
P01023	Alpha-2-macroglobulin	A2M	395.33	51	156	47	1474	163.2	11262	10669	3145	23871	8170	5151	9794	7329	11705	27224	21493	18577	10674	29162	O
P12814	Alpha-actinin-1	ACTN1	118.7	32	30	14	892	103	1554	1075	3233	9892	2292	14613	2101	909	87	2370	1379	884	1553	3282	O
P35609	Alpha-actinin-2	ACTN2	35.241	9	10	2	894	103.8															X
O43707	Alpha-actinin-4	ACTN4	68.224	18	20	6	911	104.8	234	190	676	1740	345	1481	281	203	35	266	194	123	296	251	O
P04745	Alpha-amylase 1	AMY1A; AMY1C; AMY1B	77.929	40	22	2	511	57.7	3998	7944	3473	1809	3134	1878	4001	2618	1308	1633	1618	1344	1844	1147	O
P06733	Alpha-enolase	ENO1	73.909	60	24	14	434	47.1	2089	1991	3887	8736	3150	9254	2768	2048	916	1388	1214	1125	2433	1794	O
P06280	Alpha-galactosidase A	GLA	19.033	14	5	5	429	48.7	426	415	450	326	458	486	622	370	346	386	423	381	390	501	O
Q9NZD4	Alpha-hemoglobin-stabilizing protein	AHSP	9.219	26	3	3	102	11.8	294	715	447	980	323	583	221	415	10	151	179	131	953	485	O
Q16706	Alpha-mannosidase 2	MAN2A1	110.173	26	35	25	1144	131.1	3478	3758	3693	6380	4164	5543	3889	6828	4590	4019	4863	5336	4391	4785	O
P49641	Alpha-mannosidase 2x	MAN2A2	58.219	19	15	14	1150	130.5	905	995	861	1248	819	1144	1040	1191	931	1127	1025	1107	1030	1158	O
P54802	Alpha-N-acetylglucosaminidase	NAGLU	92.104	30	19	15	743	82.2	1411	1530	1302	1488	1935	2608	1585	2680	1129	2201	1873	2164	1850	2270	O
P37840	Alpha-synuclein	SNCA	21.365	48	5	5	140	14.5	733	557	928	1628	654	1094	1253	179	290	537	449	196	1312	358	O
Q03154	Aminoacylase-1	ACY1	45.715	34	9	9	408	45.9	837	422	714	644	1239	2999	937	500	437	557	623	491	690	712	O
Q9H4A4	Aminopeptidase B	RNPEP	6.772	3	2	2	650	72.5	79	96	112	234	93	169	117	181	83	94	72	69	89	112	O
P15144	Aminopeptidase N	ANPEP	235.184	44	66	39	967	109.5	4155	5085	4537	7096	5122	8705	6758	9382	5717	6748	6135	7430	4886	7540	O
P05067	Amyloid-beta precursor protein	APP	98.857	35	33	21	770	86.9	4409	6091	6694	4503	9819	3039	4900	2145	4082	1198	3591	2665	3396	1893	O
Q06481	Amyloid-like protein 2	APLP2	21.497	8	5	5	763	86.9	259	445	308	182	423	183	281	92	143	100	175	124	139	111	O
P03950	Angiogenin	ANG	44.695	59	19	8	147	16.5	2398	3208	3512	1382	2481	1850	1787	1932	2731	2410	3153	2488	2524	1957	O
Q15389	Angiopoietin-1	ANGPT1	29.258	14	5	5	498	57.5	1246	1257	1537	1148	1681	1128	1657	612	863	284	866	602	864	338	O
Q02763	Angiopoietin-1 receptor	TEK	13.352	4	4	4	1124	125.7	105	167	122	150	152	132	150	194	202	190	168	217	163	231	O
Q6UXH0	Angiopoietin-like protein 8	C19orf80; ANGPTL8	7.189	10	2	2	198	22.1	70	77	77	64	116	109	41	76	59	74	59	28	64	66	O
Q9Y5C1	Angiopoietin-related protein 3	ANGPTL3	61.908	34	18	13	460	53.6	2697	2564	2607	1983	2588	2364	2103	2037	2090	3216	2526	2668	1961	3662	O

Q8NI99	Angiopoietin-related protein 6	ANGPTL6	31.375	26	7	7	470	51.7	287	243	261	389	245	453	302	790	749	543	491	615	875	570	O
P12821	Angiotensin-converting enzyme	ACE	76.473	17	19	19	1306	149.6	789	913	683	1027	1187	1129	1233	2158	888	2015	1561	1248	790	1059	O
P01019	Angiotensinogen	AGT	250.307	45	303	21	485	53.1	25204	29241	24922	25504	20284	28851	35427	44323	39644	30132	36482	35908	27054	38241	O
P04083	Annexin A1	ANXA1	30.943	28	8	8	346	38.7	165	128	288	6516	504	406	389	286	206	325	244	133	557	188	O
P07355	Annexin A2	ANXA2	16.1	18	5	5	339	38.6	75	68	642	113	64	71	78	75	90	83	55	86	152	68	O
P58335	Anthrax toxin receptor 2	ANTXR2	5.212	4	2	2	489	53.6	81	93	62	363	110	85	88	111	126	125	182	113	60	139	O
P03973	Antileukoproteinase	SLPI	9.02	23	4	4	132	14.3	110	155	150	53	139	78	90	103	163	120	137	140	89	188	O
P01008	Antithrombin-III	SERPINC1	455.889	65	743	45	464	52.6	34544	41108	38709	38802	29599	32812	37930	75418	53704	59434	51287	66567	46280	72408	O
P02647	Apolipoprotein A-I	APOA1	172.588	73	193	26	267	30.8	4563	5137	8169	90509	4387	14414	4403	10113	4837	7017	25425	19617	4968	7836	O
P02652	Apolipoprotein A-II	APOA2	53.633	69	55	9	100	11.2	2863	2002	1648	10366	5179	7341	3325	808	6455	1985	3691	2336	2051	2528	O
P06727	Apolipoprotein A-IV	APOA4	363.801	85	339	46	396	45.3	32117	57282	41398	15908	25675	58456	23979	67931	11637	24295	18534	27080	11562	24245	O
Q6Q788	Apolipoprotein A-V	APOA5	10.976	15	5	4	366	41.2	410	294	355	443	340	286	311	215	364	267	468	412	561	251	O
P04114	Apolipoprotein B-100	APOB	3192.734	73	2572	343	4563	515.3	299013	219447	239166	308670	169270	337764	267625	200044	291216	244638	180199	205331	295684	229263	O
P02654	Apolipoprotein C-I	APOC1	29.975	35	17	5	83	9.3	4445	999	1464	4248	1060	1373	4345	1441	4014	1274	867	1017	2395	1209	O
P02655	Apolipoprotein C-II	APOC2	42.068	56	31	5	101	11.3	2686	986	1684	19644	1275	3091	3655	1994	2036	582	603	468	1327	917	O
P02656	Apolipoprotein C-III	APOC3	46.015	59	58	8	99	10.8	17620	3525	5486	8641	4602	1047	14336	958	10285	3264	2978	2205	7940	5222	O
P55056	Apolipoprotein C-IV	APOC4; APOC4-APOC2	31.588	43	12	6	127	14.5	928	411	428	4790	246	2043	790	906	331	190	220	198	541	286	O
P05090	Apolipoprotein D	APOD	33.639	31	15	7	189	21.3	1570	1204	1062	2904	653	856	1253	785	1408	1733	1955	1620	1478	1389	O
P02649	Apolipoprotein E	APOE	194.117	79	168	33	317	36.1	6659	2681	2923	14223	3442	11019	5345	4900	8012	2629	6250	4866	3788	3122	O
Q13790	Apolipoprotein F	APOF	39.218	21	12	5	326	35.4	4585	1032	1155	1399	455	499	1720	565	2686	1786	1287	1439	1775	1080	O
O14791	Apolipoprotein L1	APOL1	50.762	36	18	14	398	43.9	1468	896	1066	6192	1392	1750	1603	860	3814	1798	2025	1832	2714	569	O
O95445	Apolipoprotein M	APOM	42.316	49	19	7	188	21.2	1601	1027	1131	3922	928	2164	1174	762	1626	749	769	681	1144	980	O
P08519	Apolipoprotein(a)	LPA	118.176	32	33	24	4548	501	8587	1334	2463	999	992	595	1511	3584	693	934	1834	1510	15374	936	O
Q9ULZ3	Apoptosis-associated speck-like protein containing a CARD	PYCARD	12.151	18	3	3	195	21.6	62	118	177	596	165	272	119	92	32	48	48	38	76	48	O
P05089	Arginase-1	ARG1	5.582	9	2	2	322	34.7															X
P04424	Argininosuccinate lyase	ASL	21.031	17	8	7	464	51.6	737	453	524	837	817	1755	729	228	267	303	356	324	395	538	O
Q86TY3	Armadillo-like helical domain-containing protein 4	C14orf37	13.218	3	2	2	774	84.1	15	36	27	13	24	27	30	24	35	42	30	40	24	41	O
P15289	Arylsulfatase A	ARSA	20.184	15	4	4	507	53.6	139	86	93	141	156	142	85	108	106	125	108	107	56	122	O
P07307	Asialoglycoprotein receptor 2	ASGR2	16.245	19	6	5	311	35.1	818	956	633	397	605	258	498	466	491	561	739	746	312	717	O
P17174	Aspartate aminotransferase, cytoplasmic	GOT1	47.554	31	11	11	413	46.2	922	785	926	1908	1073	2830	1486	2158	952	980	962	906	1227	1654	O
P00505	Aspartate aminotransferase, mitochondrial	GOT2	9.694	9	3	3	430	47.5	140	128	342	295	259	451	203	351	162	208	230	265	197	228	O
Q9BXN1	Asporin	ASPN	10.341	8	3	3	380	43.4	479	384	274	444	315	273	417	535	601	540	437	419	336	661	O
P25705	ATP synthase subunit alpha, mitochondrial	ATP5A1	64.301	29	16	14	553	59.7	871	886	1620	965	1199	620	962	827	945	946	941	1141	993	965	O
P06576	ATP synthase subunit beta, mitochondrial	ATP5B	50.604	29	11	10	529	56.5	911	903	1439	871	1167	557	810	870	953	1017	871	998	1010	815	O
P53396	ATP-citrate synthase	ACLY	19.029	6	6	5	1101	120.8	89	172	244	838	153	462	202	407	69	67	81	97	526	158	O
O75882	Attractin	ATRN	347.279	36	210	46	1429	158.4	13802	16733	14354	16240	18990	20230	18181	17807	16690	19041	17343	20636	14284	15303	O
P20160	Azurocidin	AZU1	5.79	10	2	2	251	26.9	170	252	129	232	286	148	441	142	138	80	102	92	134	105	O
P50895	Basal cell adhesion molecule	BCAM	16.736	11	4	4	628	67.4	146	285	241	152	152	115	181	203	229	236	244	233	179	331	O
P98160	Basement membrane-specific heparan sulfate proteoglycan core protein	HSPG2	285.516	17	71	55	4391	468.5	3345	4959	3529	2738	4071	2990	4023	4617	4196	4339	4270	4477	3920	5792	O
Q16620	BDNF/NT-3 growth factors receptor	NTRK2	16.25	5	3	2	822	91.9	102	176	136	110	175	96	132	137	174	167	136	143	94	221	O
P15291	Beta-1,4-galactosyltransferase 1	B4GALT1	25.022	17	4	4	398	43.9	156	175	114	157	163	119	172	170	192	106	130	145	127	170	O
O43505	Beta-1,4-glucuronyltransferase 1	B3GNT1; B4GAT1	22.142	15	4	4	415	47.1	488	499	527	458	422	545	608	796	716	595	687	791	534	761	O
P02749	Beta-2-glycoprotein 1	APOH	249.267	75	424	25	345	38.3	23095	31709	33305	19843	20647	20005	27356	26581	28853	27078	25333	28703	20554	31807	O
P61769	Beta-2-microglobulin	B2M	13.078	17	6	2	119	13.7	1309	2027	1443	995	1985	967	1319	1330	1550	1598	1558	1698	1028	2280	O
Q96KN2	Beta-Ala-His dipeptidase	CNDP1	220.591	55	136	24	507	56.7	11772	10958	12839	15809	11984	21240	13143	12372	17934	10058	13266	13215	12330	13663	O
P16278	Beta-galactosidase	GLB1	8.102	5	3	3	677	76	223	147	205	195	251	137	236	50	48	94	109	79	74	58	O
P15907	Beta-galactoside alpha-2,6-sialyltransferase 1	ST6GAL1	12.071	10	3	3	406	46.6	351	253	226	423	317	200	324	143	316	193	315	197	258	187	O
P06865	Beta-hexosaminidase subunit alpha	HEXA	18.956	11	7	7	529	60.7	329	217	228	320	249	252	221	232	154	273	228	217	240	199	O
P07686	Beta-hexosaminidase subunit beta	HEXB	24.586	15	7	7	556	63.1	636	688	915	1063	740	1057	766	956	433	988	795	786	676	705	O
O00462	Beta-mannosidase	MANBA	40.373	14	10	9	879	100.8	575	660	671	521	696	642	515	772	493	578	545	539	518	696	O
Q9HBI1	Beta-parvin	PARVB	16.512	12	4	4	364	41.7	616	303	591	851	733	3153	871	83	11	823	452	122	235	498	O
P31939	Bifunctional purine biosynthesis protein ATIC	ATIC	6.241	4	2	2	592	64.6	114	60	64	181	43	176	54	126	29	55	43	29	68	42	O
P21810	Biglycan	BGN	13.996	14	4	3	368	41.6	314	232	249	283	357	184	282	208	237	210	244	251	214	278	O
P53004	Biliverdin reductase A	BLVRA	9.971	7	2	2	296	33.4	196	174	156	570	117	412	219	266	92	138	98	73	361	149	O
P43251	Biotinidase	BTD	176.674	35	83	15	543	61.1	6335	6446	6233	5848	6162	6777	8226	5908	9012	7483	7128	6328	6959	8011	O
P07738	Bisphosphoglycerate mutase	BPGM	35.8	27	9	6	259	30	307	570	615	2266	401	1504	604	1616	297	295	255	346	1622	547	O
Q13867	Bleomycin hydrolase	BLMH	32.045	23	9	7	455	52.5	419	631	916	1185	651	1468	549	1231	705	603	566	737	634	539	O
P13727	Bone marrow proteoglycan	PRG2	38.707	30	10	7	222	25.2	1267	999	514	1131	1243	680	1022	1635	2298	899	797	936	881	1699	O
P13497	Bone morphogenetic protein 1	BMP1	67.993	17	17	16	986	111.2	1245	1435	1298	2336	1249	2251	2032	1484	1588	1031	1389	1596	1096	1573	O
P80723	Brain acid soluble protein 1	BASP1	12.349	25	3	3	227	22.7	151	309	261	251	253	209	156	216	169	226	177	182	142	177	O
Q9UBW5	Bridging integrator 2	BIN2	17.905	11	6	5	565	61.8	206	161	647	173	379	261	492	3		258	137	6	27	82	O
Q96CX2	BTB/POZ domain-containing protein KCTD12	KCTD12	8.667	7	2	2	325	35.7	173	244	204	208	225	166	195	456	242	268	220	217	176	235	O
P04003	C4b-binding protein alpha chain	C4BPA	282.583	57	388	30	597	67	52560	57655	52367	30123	40088	43203	59039	29522	44604	28968	45362	44068	42084	34227	O
P20851	C4b-binding protein beta chain	C4BPB	135.017	55	113	12	252	28.3	11767	14737	12991	9593	10253	9860	16240	7175	12503	8044	11170	11464	10381	9287	O
P12830	Cadherin-1	CDH1	54.069	15	11	10	882	97.4	311	299	350	205	458	256	311	266	321	543	344	245	313	844	O
P55287	Cadherin-11	CDH11	7.754	3	2	2	796	87.9	374	508	391	268	534	230	476	371	492	477	459	417	413	636	O
P55290	Cadherin-13	CDH13	69.293	22	21	15	713	78.2	2103	4238	2768	2452	3445	1835	2564	2949	3126	4133	3236	3433	2214	4442	O
Q12864	Cadherin-17	CDH17	10.706	5	4	4	832	92.2	266	344	626	450	451	237	508	231	181	2056	1113	209	386	2138	O
Q9H159	Cadherin-19	CDH19	8.55	4	3	3	772	86.9	287	269	314	191	238	179	248	291	303	277	236	268	273	348	O
P19022	Cadherin-2	CDH2	61.088	20	19	12	906	99.7	1497	3010	2532	1623	2788	2135	2179	2361	2672	2455	2627	2467	1809	3883	O
Q9H251	Cadherin-23	CDH23	11.607	2	4	4	3354	369.3	83	65	59	53	51	53	71	63	87	72	109	92	58	97	O
P33151	Cadherin-5	CDH5	109.842	26	47	16	784	87.5	3005	5873	3824	2656	4546	2195	3909	3305	4349	5590	4820	4194	2459	5866	O
P55285	Cadherin-6	CDH6	40.339	12	8	8	790	88.3	1097	1181	815	694	1318	688	1243	1036	1589	1406	1016	1160	894	1437	O
Q9BYE9	Cadherin-related family member 2	CDHR2	95.554	20	24	19	1310	141.5	2298	1207	1190	1000	5569	4944	2580	330	682	778	820	869	807	1105	O
Q9HBB8	Cadherin-related family member 5	CDHR5	28.803	8	6	5	845	88.2	697	833	557	276	917	423	794	504	924	457	472	433	605	507	O
Q05682	Caldesmon	CALD1	11.756	6	4	4	793	93.2	341	205	717	181	414	230	407	39	23	125	93	39	54	124	O
P0DP25	Calmodulin-3	CALM3; CALM2; CALM1	20.589	68	8	6	149	16.8	250	279	550	574	463	773	562	122	56	387	271	104	305	250	O
P27482	Calmodulin-like protein 3	CALML3	13.536	38	5	3	149	16.9	13	11	574	40	14	14	21	20	17	27	13	21	28	21	O
Q9NZT1	Calmodulin-like protein 5	CALML5	18.395	32	3	2	146	15.9	67	98	455	48	223	101	85	123	134	148	76	132	129	144	O
P27824	Calnexin	CANX	8.715	4	2	2	592	67.5	128	192	223	174	158	110	168	221	157	185	162	172	119	203	O
P07384	Calpain-1 catalytic subunit	CAPN1	18.514	12	5	5	714	81.8	18	22	21	47	20	26	18	22	15	23	9	8	37	22	O
P17655	Calpain-2 catalytic subunit	CAPN2	9.92	3	2	2	700	79.9	80	92	98	132	89	159	91	206	85	105	90	90	99	90	O
Q99439	Calponin-2	CNN2	8.61	9	2	2	309	33.7	628	234	438	1031	858	418	904	17	17	290	205	61	70	138	O
P27797	Calreticulin	CALR	63.739	47	21	15	417	48.1	1610	2005	1991	1540	1746	1350	1573	2292	1502	2132	1731	1511	1580	2165	O
O94985	Calsyntenin-1	CLSTN1	62.422	19	19	16	981	109.7	2009	2347	2126	1458	2349	1824	2150	2132	2462	2335	2146	2325	1534	2619	O
O43852	Calumenin	CALU	57.233	35	14	9	315	37.1	1293	1870	2049	1343	2101	1006	1752	666	1024	481	1136	820	909	593	O
Q9NRB3	Carbohydrate sulfotransferase 12	CHST12	5.724	5	2	2	414	48.4	53	72	90	60	84	64	77	49	63	63	68	71	48	59	O
Q7LGC8	Carbohydrate sulfotransferase 3	CHST3	7.653	10	3	3	479	54.7	156	208	219	156	185	123	173	193	194	212	199	230	143	268	O
P00915	Carbonic anhydrase 1	CA1	112.782	59	65	12	261	28.9	3841	7924	8902	18950	4247	10933	7992	13876	4152	3285	3006	2515	19873	7276	O
P00918	Carbonic anhydrase 2	CA2	66.986	54	20	10	260	29.2	1021	1996	2270	5531	1066	4163	1876	3563	868	1011	795	612	5181	1547	O
P07451	Carbonic anhydrase 3	CA3	7.639	13	3	3	260	29.5	172	252	297	567	393	257	294	699	243	282	175	130	480	321	O
Q6UWW8	Carboxylesterase 3	CES3	9.303	6	2	2	571	62.2	126	103	111	199	125	244	167	87	107	96	116	118	183	103	O
P15085	Carboxypeptidase A1	CPA1	12.656	13	4	4	419	47.1	781	689	431	593	555	539	1045	1505	658	420	389	492	852	570	O
P15086	Carboxypeptidase B	CPB1	4.656	6	2	2	417	47.3	46	42	33	42	47	42	45	57	35	24	31	33	42	28	O
Q96IY4	Carboxypeptidase B2	CPB2	100.744	49	82	19	423	48.4	9261	10607	9167	10237	7916	13670	11804	17690	10716	10420	10876	15786	11347	12781	O
O75976	Carboxypeptidase D	CPD	7.978	2	2	2	1380	152.8															X
P14384	Carboxypeptidase M	CPM	9.941	7	3	3	443	50.5	245	269	211	242	304	417	319	318	243	281	237	277	272	398	O
P15169	Carboxypeptidase N catalytic chain	CPN1	130.464	50	69	17	458	52.3	6916	6093	5064	5565	4246	7824	6049	7440	7685	6110	6303	7347	5168	5963	O
P22792	Carboxypeptidase N subunit 2	CPN2	139.394	51	99	18	545	60.5	11934	12147	9699	9020	8915	12160	10136	9599	14105	10744	11804	13137	8849	12602	O
Q9Y646	Carboxypeptidase Q	CPQ	34.018	20	8	8	472	51.9	605	673	947	509	1014	976	684	1069	793	838	647	896	597	834	O
P13688	Carcinoembryonic antigen-related cell adhesion molecule 1	CEACAM1	12.046	6	2	2	526	57.5	56	98	101	44	105	64	76	72	67	76	97	81	56	101	O

Q9NQ79	Cartilage acidic protein 1	CRTAC1	133.879	46	44	22	661	71.4	2218	2486	2667	1283	1830	1698	2056	3489	1869	3655	2624	2647	1629	3126	O
O75339	Cartilage intermediate layer protein 1	CILP	29.933	8	7	7	1184	132.5	73	133	105	58	141	50	84	102	133	117	112	94	115	166	O
Q8IUL8	Cartilage intermediate layer protein 2	CILP2	14.402	5	5	5	1156	126.2	241	491	338	432	470	311	351	316	526	459	468	499	167	506	O
P49747	Cartilage oligomeric matrix protein	COMP	180.216	56	57	25	757	82.8	2572	5791	2755	2904	3629	2537	3772	3899	3875	4363	3672	2692	2962	5343	O
P31944	Caspase-14	CASP14	41.852	37	9	9	242	27.7	296	309	1338	298	464	537	442	544	466	812	268	574	445	753	O
P04040	Catalase	CAT	74.621	42	28	19	527	59.7	941	1451	1272	4911	1021	3451	1428	3396	851	757	677	735	2850	1281	O
P49913	Cathelicidin antimicrobial peptide	CAMP	10.008	18	4	3	170	19.3	134	85	80	192	127	105	156	83	158	43	27	36	94	81	O
P07858	Cathepsin B	CTSB	25.486	25	7	6	339	37.8	626	809	769	483	881	560	699	798	760	975	910	959	696	1470	O
P07339	Cathepsin D	CTSD	74.63	43	21	13	412	44.5	3174	3908	4205	2855	3956	4827	3496	2398	2426	2991	3044	2594	2743	3998	O
Q9UBX1	Cathepsin F	CTSF	23.421	17	7	7	484	53.3	616	907	1007	437	615	934	756	695	636	1011	1062	1083	479	1658	O
P08311	Cathepsin G	CTSG	10.444	16	3	3	255	28.8	83	160	96	962	231	103	162	77	64	47	42	43	89	38	O
P07711	Cathepsin L1	CTSL; CTSL1	19.282	19	5	5	333	37.5	189	225	224	204	250	160	202	237	274	255	244	232	250	374	O
P25774	Cathepsin S	CTSS	15.036	14	4	4	331	37.5	269	264	251	258	395	377	352	439	272	271	225	308	248	315	O
Q9UBR2	Cathepsin Z	CTSZ	7.028	7	2	2	303	33.8	42	62	52	32	48	30	34	29	24	58	43	36	25	37	O
P11717	Cation-independent mannose-6-phosphate receptor	IGF2R	215.03	27	54	52	2491	274.2	4672	5114	6306	4199	5460	5309	6036	6444	6660	6112	5913	6600	4223	5836	O
O95810	Caveolae-associated protein 2	SDPR; CAVIN2	23.386	13	6	6	425	47.1	119	85	260	109	164	358	181	12	8	118	68	28	37	123	O
Q16627	C-C motif chemokine 14	CCL14	8.062	30	3	2	93	10.7	187	237	188	139	184	130	213	125	284	157	194	214	117	183	O
O15467	C-C motif chemokine 16	CCL16	13.052	33	3	3	120	13.6	298	315	447	186	225	210	308	125	355	285	343	272	211	253	O
P29279	CCN family member 2	CTGF	41.531	32	9	9	349	38.1	587	948	1031	621	792	527	826	410	631	205	460	347	405	386	O
O76076	CCN family member 5	WISP2	6.009	7	2	2	250	26.8															X
Q6YHK3	CD109 antigen	CD109	153.056	28	38	31	1445	161.6	2818	4640	3433	3263	3520	4025	3855	6284	5226	5227	5042	4770	3574	6050	O
Q13740	CD166 antigen	ALCAM	77.462	37	17	14	583	65.1	1570	2298	2119	1461	2896	1427	1797	2069	2284	2432	2173	2631	1442	2893	O
Q15762	CD226 antigen	CD226	15.631	10	2	2	336	38.6	216	312	742	138	372	92	319	101	142	119	126	80	72	149	O
P16070	CD44 antigen	CD44	17.013	5	11	4	742	81.5	1871	3353	2180	1155	3174	1193	1637	2099	2147	2973	2410	2632	1358	3068	O
P09326	CD48 antigen	CD48	5.408	10	2	2	243	27.7	42	27	21	39	27	15	30	25	35	22	20	28	30	37	O
O43866	CD5 antigen-like	CD5L	89.953	58	45	16	347	38.1	3868	5400	5210	5702	8412	5584	15194	4156	7622	7209	6148	9423	5367	4453	O
P13987	CD59 glycoprotein	CD59	9.505	19	2	2	128	14.2	127	200	132	87	197	114	132	137	180	164	150	174	121	270	O
P60033	CD81 antigen	CD81	10.448	15	2	2	236	25.8	124	135	119	126	114	235	144	163	226	129	128	187	151	133	O
P21926	CD9 antigen	CD9	8.083	15	2	2	228	25.4	501	397	827	251	523	873	614	179	108	185	170	114	163	142	O
Q9BY67	Cell adhesion molecule 1	CADM1	41.034	21	11	6	442	48.5	818	1426	991	702	1343	585	890	974	1155	1092	1041	1145	649	1430	O
Q4KMG0	Cell adhesion molecule-related/down-regulated by oncogenes	CDON	28.876	5	5	5	1287	139.1	278	520	388	229	479	330	318	441	373	374	317	396	225	465	O
Q8WUJ3	Cell migration-inducing and hyaluronan-binding protein	KIAA1199; CEMIP	6.871	2	2	2	1361	152.9	66	70	75	68	87	88	60	113	65	88	64	80	64	101	O
P43121	Cell surface glycoprotein MUC18	MCAM	99.326	35	32	18	646	71.6	2786	5164	3810	1489	3187	2673	3316	3902	3282	4665	4704	4138	2765	5268	O
P23435	Cerebellin-1	CBLN1	7.917	19	2	2	193	21.1	121	316	223	182	341	406	558	714	314	200	429	254	191	170	O
Q9NTU7	Cerebellin-4	CBLN4	4.931	9	2	2	201	21.8	108	76	79	57	149	136	110	102	109	109	78	141	63	114	O
O75503	Ceroid-lipofuscinosis neuronal protein 5	CLN5	7.126	6	2	2	358	41.5	254	177	184	158	170	249	323	288	245	363	442	382	240	357	O
P00450	Ceruloplasmin	CP	878.343	74	1264	71	1065	122.1	94942	79032	60944	82958	71603	79468	79794	113888	80800	94582	92011	90135	92613	65253	O
P36222	Chitinase-3-like protein 1	CHI3L1	29.479	22	6	6	383	42.6	435	413	197	383	581	543	236	524	208	618	536	356	630	1636	O
Q13231	Chitotriosidase-1	CHIT1	7.461	8	2	2	466	51.6		50	5	27	8	13	83	58	76	63	78	15	38		O
O00299	Chloride intracellular channel protein 1	CLIC1	35.462	52	8	8	241	26.9	809	718	1418	1993	1045	4368	1076	438	128	688	460	254	806	788	O
P11597	Cholesteryl ester transfer protein	CETP	75.543	26	29	13	493	54.7	6709	5821	4363	1363	4161	923	6056	932	6971	4730	5402	6405	5465	6803	O
P06276	Cholinesterase	BCHE	156.03	45	83	23	602	68.4	6717	8710	6414	8440	4765	11998	8864	10411	11814	7288	7594	8662	9771	9602	O
O15335	Chondroadherin	CHAD	19.81	18	6	5	359	40.5	361	450	228	497	494	387	377	591	545	532	619	571	589	679	O
Q6UVK1	Chondroitin sulfate proteoglycan 4	CSPG4	37.707	6	8	8	2322	250.4	278	196	159	172	162	212	231	374	240	234	209	230	153	219	O
P10645	Chromogranin-A	CHGA	42.982	26	10	9	457	50.7	1234	1173	1163	570	1062	357	691	909	583	1415	964	875	570	1653	O
P26992	Ciliary neurotrophic factor receptor subunit alpha	CNTFR	18.3	15	5	4	372	40.6	414	679	699	621	817	636	641	949	763	644	568	733	457	1260	O
O75390	Citrate synthase, mitochondrial	CS	9.666	7	4	3	466	51.7	150	154	241	176	255	120	202	193	196	180	207	218	193	188	O
Q00610	Clathrin heavy chain 1	CLTC	4.002	1	2	2	1675	191.5	43	54	207	170	54	172	61	47	32	33	30	42	86	83	O
P10909	Clusterin	CLU	231.464	46	244	25	449	52.5	25915	23430	25851	22514	22291	22520	26083	20400	28688	24913	26504	26829	23493	25908	O
Q92187	CMP-N-acetylneuraminate-poly-alpha-2,8-sialyltransferase	ST8SIA4	8.024	8	3	3	359	41.3	252	351	279	227	395	295	280	246	247	256	286	288	220	346	O
Q14019	Coactosin-like protein	COTL1	9.38	26	6	4	142	15.9	532	266	558	1129	726	791	597	209	57	456	295	99	165	229	O
P00740	Coagulation factor IX	F9	108.25	46	49	16	461	51.7	4730	5461	4617	1420	5209	2852	3911	2257	3207	4343	3088	4428	4387	6191	O
P12259	Coagulation factor V	F5	482.151	38	185	74	2224	251.5	22594	25877	29293	13877	18502	20147	15006	17756	24814	30989	29017	27863	18554	26625	O
P08709	Coagulation factor VII	F7	51.701	23	13	7	466	51.6	1423	1331	1207	521	1319	645	1545	779	747	1146	867	1076	1203	1852	O
P00451	Coagulation factor VIII	F8	38.262	5	9	9	2351	266.8	1388	901	994	1419	720	727	616	1040	1628	1414	1379	1286	1096	1192	O
P00742	Coagulation factor X	F10	205.753	48	111	24	488	54.7	6277	9395	8556	5171	6717	5605	7468	7202	9046	7566	8807	7547	5136	9767	O
P03951	Coagulation factor XI	F11	229.357	64	85	36	625	70.1	7013	8304	7873	4324	4216	6987	3875	7053	10179	8472	8662	10307	6325	7565	O
P00748	Coagulation factor XII	F12	150.106	42	117	23	615	67.7	6477	4809	5087	5854	7566	6062	7724	6799	7054	6949	7844	7756	6057	6177	O
P00488	Coagulation factor XIII A chain	F13A1	183.309	45	49	26	732	83.2	3111	2871	2181	5732	3233	8771	4371	7692	3082	5059	6463	6532	3372	11077	O
P05160	Coagulation factor XIII B chain	F13B	322.711	65	162	39	661	75.5	12512	17579	15775	8424	15680	8485	14896	9348	12755	13198	14915	13638	8539	15880	O
P23528	Cofilin-1	CFL1	34.854	60	13	10	166	18.5	2066	1348	2489	3605	2675	5386	3450	215	320	1193	850	450	1374	1058	O
Q96EE4	Coiled-coil domain-containing protein 126	CCDC126	11.681	16	3	2	140	15.7	1230	371	320	2221	353	169	351	353	384	425	390	342	320	368	O
P02452	Collagen alpha-1(I) chain	COL1A1	34.3	6	8	7	1464	138.9	567	1177	798	441	1625	435	658	1377	1218	875	1040	834	939	1334	O
P02461	Collagen alpha-1(III) chain	COL3A1	30.178	5	6	6	1466	138.5	213	385	333	234	438	219	221	313	276	259	271	249	188	333	O
P20908	Collagen alpha-1(V) chain	COL5A1	6.544	2	3	3	1838	183.4	246	454	205	202	453	211	238	300	422	308	231	274	278	449	O
P12109	Collagen alpha-1(VI) chain	COL6A1	82.406	18	17	13	1028	108.5	1325	696	1501	1044	1638	1119	2041	1434	1861	1018	938	1107	1773	1408	O
Q99715	Collagen alpha-1(XII) chain	COL12A1	38.668	6	12	12	3063	332.9	766	823	550	780	600	590	573	1272	685	917	877	861	633	1100	O
P39059	Collagen alpha-1(XV) chain	COL15A1	13.832	4	5	5	1388	141.6	343	583	338	439	514	411	320	430	457	430	416	442	384	581	O
P39060	Collagen alpha-1(XVIII) chain	COL18A1	54.398	6	14	9	1754	178.1	2542	2715	2139	2433	2579	1629	2471	1714	3504	2619	3565	3120	1972	2771	O
P08123	Collagen alpha-2(I) chain	COL1A2	12.334	2	3	3	1366	129.2	87	155	140	71	216	66	97	227	124	96	116	108	104	144	O
P12110	Collagen alpha-2(VI) chain	COL6A2	14.885	4	4	4	1019	108.5	84	156	89	115	149	56	75	110	120	105	91	100	75	134	O
P12111	Collagen alpha-3(VI) chain	COL6A3	357.33	31	95	76	3177	343.5	7553	8319	6236	9588	7751	7126	7203	14283	10395	11548	8484	9642	7376	13231	O
Q9Y6Z7	Collectin-10	COLEC10	22.637	24	11	8	277	30.7	612	672	620	634	937	607	650	671	694	680	710	883	558	807	O
Q9BWP8	Collectin-11	COLEC11	39.151	24	11	6	271	28.6	770	845	905	835	1190	937	649	1186	832	865	958	1320	895	1120	O
P02745	Complement C1q subcomponent subunit A	C1QA	78.252	40	54	8	245	26	3134	2912	2608	2639	2826	1961	3585	2243	3046	3126	2818	3022	3366	2749	O
P02746	Complement C1q subcomponent subunit B	C1QB	148.96	48	183	11	253	26.7	8087	8476	7754	5643	8242	6383	10476	6673	8508	8717	8200	8814	9152	7809	O
P02747	Complement C1q subcomponent subunit C	C1QC	39.196	28	33	7	245	25.8	2285	2280	2126	1626	2550	2035	3088	1788	2489	2521	2402	2801	2726	2226	O
Q9BXJ0	Complement C1q tumor necrosis factor-related protein 5	C1QTNF5	12.464	10	2	2	243	25.3	208	242	147	216	184	170	208	300	192	230	173	159	144	243	O
P00736	Complement C1r subcomponent	C1R	317.155	54	265	32	705	80.1	25393	19551	22233	18979	23239	19274	23243	24612	20174	25996	20816	21090	27228	18471	O
Q9NZP8	Complement C1r subcomponent-like protein	C1RL	82.454	36	45	14	487	53.5	4160	3259	3180	114	4685	2849	4040	2999	3924	3443	3291	3368	3592	4372	O
P09871	Complement C1s subcomponent	C1S	344.74	54	339	31	688	76.6	28141	26861	26855	22485	28372	22978	27624	26592	23032	31571	26696	26336	29858	23219	O
P06681	Complement C2	C2	355.9	58	276	39	752	83.2	23569	23363	22524	22658	21606	23068	27766	32314	27809	26832	25552	26991	28269	27694	O
P01024	Complement C3	C3	2254.635	90	4732	188	1663	187	428448	272990	272119	391336	348252	436736	361712	368613	413006	329926	313375	299513	473459	235586	O
P0C0L4	Complement C4-A	C4A; C4B	1279.075	74	2019	5	1744	192.7	192710	144310	154316	153154	130564	185059	93625	145096	145119	187730	147018	161466	185769	98413	O
P0C0L5	Complement C4-B	C4B; C4B_2	1286.002	74	2055	5	1744	192.6	9368	8858	9080	10097	7305	11628	3289	12976	3622	7029	7280	8840	10462	5517	O
P01031	Complement C5	C5	869.898	68	732	105	1676	188.2	66613	40962	41974	54486	38395	64582	55933	83894	51828	56188	52072	56683	60838	47777	O
Q9NPY3	Complement component C1q receptor	CD93	47.114	20	12	10	652	68.5	885	1392	840	747	1352	445	780	942	991	1216	1101	1070	533	1573	O
P13671	Complement component C6	C6	424.417	64	256	53	934	104.7	26632	31358	26625	21485	23325	22745	25255	25942	30172	25304	24254	27285	26387	24981	O
P10643	Complement component C7	C7	461.387	67	347	49	843	93.5	15432	17393	18861	17345	20916	19590	19747	23328	19386	31423	26411	24446	15388	29869	O
P07357	Complement component C8 alpha chain	C8A	349.28	61	340	34	584	65.1	24564	25621	21103	20666	23311	19668	26556	30029	24202	26640	23991	24134	26630	22096	O
P07358	Complement component C8 beta chain	C8B	297.013	67	208	37	591	67	14286	13020	10593	10708	12665	10848	14519	15988	12808	13382	12144	11605	14920	12974	O
P07360	Complement component C8 gamma chain	C8G	119.481	80	100	14	202	22.3	9169	8512	6960	6781	7924	6956	8542	7732	7274	8147	7284	7567	7945	7516	O
P02748	Complement component C9	C9	265.811	59	176	37	559	63.1	26482	13197	13563	7574	18721	8525	12682	19562	9927	18373	11916	14210	23397	13527	O
P08174	Complement decay-accelerating factor	CD55	30.985	24	18	8	381	41.4	2272	3321	2389	1744	2612	1414	2227	2399	2364	2525	2345	2165	1715	3076	O
P00751	Complement factor B	CFB	518.293	65	535	61	764	85.5	50455	52572	50186	34356	51745	41446	37848	57837	22257	50671	44179	43666	51534	43733	O
P00746	Complement factor D	CFD	102.603	57	30	12	253	27	2608	4277	2950	2425	3807	2554	2667	2586	3703	3576	3647	3550	2557	5338	O
P08603	Complement factor H	CFH	953.616	68	1472	75	1231	139	84981	110308	104063	57231	111421	72386	91103	66923	79513	89861	92067	90977	84821	84433	O
Q03591	Complement factor H-related protein 1	CFHR1	132.303	58	156	2	330	37.6	3239	4710	3974	2969	3597	2605	4491	2903	4249	4925	4167	4699	3761	3933	O
P36980	Complement factor H-related protein 2	CFHR2	72.322	57	50	5	270	30.6	1331	1918	1713	1713	1450	1446	3126	914	3146	2889	2877	2295	2233	1462	O
Q02985	Complement factor H-related protein 3	CFHR3	97.218	47	67	3	330	37.3	1376	2940	3580	1218	1024	321	2923	1173	2177	2565	2567	1023	1004	1409	O
Q92496	Complement factor H-related protein 4	CFHR4	53.458	27	29	4	578	65.3	66	39	51	397	31	39	71	62	160	188	310	149	211	38	O

Q9BXR6	Complement factor H-related protein 5	CFHR5	94.669	44	35	18	569	64.4	4408	4190	3545	2067	3079	2648	3667	2742	2733	4517	4329	4499	3383	3516	O
P05156	Complement factor I	CFI	358.52	51	322	31	583	65.7	30813	33409	31632	19394	33587	22495	29954	22313	27443	25092	26528	27066	27697	30861	O
P17927	Complement receptor type 1	CR1	23.212	8	5	5	2039	223.5	176	643	499	284	758	300	469	360	532	724	576	550	280	767	O
P20023	Complement receptor type 2	CR2	91.8	29	32	26	1033	112.8	786	3882	2009	1297	3932	1756	1608	1073	2610	2818	2666	2242	1484	4229	O
Q12860	Contactin-1	CNTN1	126.211	31	34	25	1018	113.2	3397	3275	3215	3643	3021	3409	3512	4759	3883	3781	3632	4252	2741	5869	O
Q9P232	Contactin-3	CNTN3	71.998	21	18	15	1028	112.8	1282	2290	2054	1311	1691	2576	1895	1755	2600	2218	1864	2586	1803	2676	O
Q8IWV2	Contactin-4	CNTN4	85.257	25	19	16	1026	113.4	1037	1116	812	930	1129	1125	1182	1562	1260	1590	1324	1511	929	1551	O
P31146	Coronin-1A	CORO1A	29.235	19	8	7	461	51	321	316	324	2449	389	1640	440	491	162	371	277	218	391	247	O
Q9ULV4	Coronin-1C	CORO1C	17.04	10	4	4	474	53.2	182	95	222	435	196	982	159	53		237	125	28	83	219	O
P08185	Corticosteroid-binding globulin	SERPINA6	169.086	48	110	16	405	45.1	8405	8421	9406	10387	8074	12346	9953	14475	9782	8649	12100	10405	10046	13192	O
P24387	Corticotropin-releasing factor-binding protein	CRHBP	20.304	27	6	6	322	36.1	475	491	457	285	557	481	470	346	428	466	348	375	470	507	O
P02741	C-reactive protein	CRP	35.525	21	14	5	224	25	1397	1161	173	26	1213	934	331	203	27	165	55	110	2424	12	O
P06732	Creatine kinase M-type	CKM	36.681	31	13	11	381	43.1	207	329	254	605	655	731	296	887	267	276	267	273	357	393	O
P17540	Creatine kinase S-type, mitochondrial	CKMT2	27.593	21	9	5	419	47.5	287	310	496	408	491	253	440	367	402	352	421	420	431	440	O
P46109	Crk-like protein	CRKL	10.334	9	2	2	303	33.8	512	442	691	636	992	809	1083	83	54	301	215	99	210	256	O
Q9P126	C-type lectin domain family 1 member B	CLEC1B	5.19	8	2	2	229	26.6															X
Q9Y240	C-type lectin domain family 11 member A	CLEC11A	10.988	10	3	3	323	35.7	178	213	191	144	253	127	186	123	193	129	182	157	139	172	O
Q9UBG0	C-type mannose receptor 2	MRC2	44.06	10	12	10	1479	166.6	492	839	580	584	930	597	621	703	888	900	783	879	533	1091	O
P18850	Cyclic AMP-dependent transcription factor ATF-6 alpha	ATF6	18.433	7	6	5	670	74.5	42	53	58	33	59	21	42	47	48	61	60	43	24	57	O
Q99941	Cyclic AMP-dependent transcription factor ATF-6 beta	ATF6B	24.093	11	6	6	703	76.7	115	130	147	91	167	96	120	88	106	150	119	123	94	166	O
Q9NUQ9	CYFIP-related Rac1 interactor B	FAM49B	10.14	12	3	3	324	36.7															X
P01040	Cystatin-A	CSTA	7.035	35	2	2	98	11	235	363	545	353	484	271	374	299	389	422	199	394	321	417	O
P04080	Cystatin-B	CSTB	8.235	24	2	2	98	11.1	299	501	767	183	459	188	198	208	135	93	81	117	166	226	O
P01034	Cystatin-C	CST3	86.155	63	32	9	146	15.8	1348	1903	1508	930	1714	810	1291	1125	1550	1567	1605	1449	1222	2215	O
Q15828	Cystatin-M	CST6	38.354	58	7	7	149	16.5	491	492	872	211	430	363	422	704	550	579	671	727	387	468	O
P01036	Cystatin-S	CST4	17.86	31	4	3	141	16.2	701	817	713	225	443	85	304	570	255	318	432	148	1372	258	O
P21291	Cysteine and glycine-rich protein 1	CSRP1	15.171	17	2	2	193	20.6	208	85	227	189	232	146	320	10	14	92	67	14	35	63	O
P54108	Cysteine-rich secretory protein 3	CRISP3	29.053	39	23	7	245	27.6	790	1129	964	597	1328	700	1030	1213	1091	1198	1149	1328	1090	1807	O
P00167	Cytochrome b5	CYB5A	11.338	20	2	2	134	15.3	79	313	335	388	140	294	172	271	96	78	79	57	398	227	O
P31930	Cytochrome b-c1 complex subunit 1, mitochondrial	UQCRC1	7.644	6	2	2	480	52.6	77	53	104	69	94	43	76	56	78	69	61	81	71	71	O
P99999	Cytochrome c	CYCS	9.956	30	4	3	105	11.7	368	494	648	956	673	902	516	913	447	493	473	507	427	829	O
Q9NRR1	Cytokine-like protein 1	CYTL1	11.912	18	2	2	136	15.6	136	198	192	121	147	88	174	148	248	167	180	184	116	258	O
P21399	Cytoplasmic aconitate hydratase	ACO1	10.155	3	2	2	889	98.3	84	78	77	95	100	201	118	69	63	67	48	40	82	100	O
P30046	D-dopachrome decarboxylase	DDT	20.535	47	5	5	118	12.7	209	224	242	599	178	658	177	405	264	86	105	125	606	168	O
P07585	Decorin	DCN	12.569	12	3	2	359	39.7	293	293	299	282	398	202	332	248	269	250	296	309	219	346	O
P13716	Delta-aminolevulinic acid dehydratase	ALAD	30.426	23	8	7	330	36.3	155	229	171	889	132	735	221	594	56	99	63	90	736	211	O
Q07507	Dermatopontin	DPT	23.56	35	5	5	201	24	216	443	364	357	538	304	409	418	544	401	352	404	290	428	O
P81605	Dermcidin	DCD	10.623	26	3	3	110	11.3	391	685	365	400	817	153	337	242	473	318	274	309	274	481	O
P17661	Desmin	DES	10.804	8	4	2	470	53.5															X
Q08554	Desmocollin-1	DSC1	18.957	7	7	6	894	99.9	132	169	157	47	114	76	101	179	141	118	106	104	97	127	O
Q02487	Desmocollin-2	DSC2	32.813	11	9	7	901	99.9	463	734	610	282	621	245	426	468	460	543	435	460	352	635	O
Q14574	Desmocollin-3	DSC3	8.447	2	3	2	896	99.9	439	686	568	187	398	230	410	250	232	289	269	364	217	432	O
Q14126	Desmoglein-2	DSG2	89.157	16	21	15	1118	122.2	1126	2221	1511	1054	1337	813	1160	1351	1463	1679	1475	1778	1278	1929	O
P15924	Desmoplakin	DSP	24.117	3	5	5	2871	331.6	17	19	285	27	38	31	26	9	19	10	16	18	47	14	O
Q9UBP4	Dickkopf-related protein 3	DKK3	27.183	15	6	4	350	38.4	381	551	451	305	402	382	446	486	377	454	460	512	254	541	O
P09417	Dihydropteridine reductase	QDPR	10.683	11	2	2	244	25.8	103	99	113	264	134	396	108	209	87	93	75	62	160	112	O
Q01459	Di-N-acetylchitobiase	CTBS	73.402	42	23	11	385	43.7	1572	2189	2470	1555	2650	1478	2464	1859	2124	2804	2543	2362	2256	2190	O
Q9H4A9	Dipeptidase 2	DPEP2	69.735	30	14	11	486	53.3	665	1018	806	647	1104	723	957	947	912	940	1009	1006	600	1053	O
P53634	Dipeptidyl peptidase 1	CTSC	33.147	18	7	7	463	51.8	1017	899	1136	1806	1272	1070	1082	937	620	887	854	994	679	1351	O
Q9UHL4	Dipeptidyl peptidase 2	DPP7	6.093	4	2	2	492	54.3															X
Q9NY33	Dipeptidyl peptidase 3	DPP3	6.192	3	4	3	737	82.5	138	178	179	454	174	532	199	262	112	139	120	103	255	200	O
P27487	Dipeptidyl peptidase 4	DPP4	62.881	24	26	19	766	88.2	1348	1793	1345	321	1972	2629	1830	3199	2393	2174	1977	2237	1663	2085	O
Q96PD2	Discoidin, CUB and LCCL domain-containing protein 2	DCBLD2	8.225	3	2	2	775	85	39	48	47	57	45	39	52	60	49	59	48	50	49	89	O
Q13217	DnaJ homolog subfamily C member 3	DNAJC3	10.916	7	3	3	504	57.5	115	152	166	199	129	139	163	133	160	95	129	118	132	108	O
Q9P2X0	Dolichol-phosphate mannosyltransferase subunit 3	DPM3	3.806	24	2	2	92	10.1	22	21	16	32	19	30	13	17	24	24	28	25	25	20	O
P09172	Dopamine beta-hydroxylase	DBH	114.485	41	35	20	617	69	4506	349	3050	7548	6562	1595	6645	2173	2192	2995	1495	6304	4332	4789	O
Q9UJU6	Drebrin-like protein	DBNL	7.767	9	2	2	430	48.2	105	77	144	244	204	194	221	22	16	68	54	12	43	33	O
Q14118	Dystroglycan	DAG1	47.779	11	9	8	895	97.4	1176	2234	1683	981	1957	1135	1452	1421	1649	1619	1612	1567	1171	2198	O
Q13508	Ecto-ADP-ribosyltransferase 3	ART3	36.137	24	10	7	389	43.9	1359	2059	1613	1154	1310	926	1288	2397	1418	1591	1818	1987	1525	3703	O
Q93070	Ecto-ADP-ribosyltransferase 4	ART4	21.3	20	6	5	314	35.9	606	679	812	589	826	620	745	653	789	726	733	673	492	1192	O
O75356	Ectonucleoside triphosphate diphosphohydrolase 5	ENTPD5	22.756	19	5	5	428	47.5	710	487	510	315	439	502	605	430	576	539	439	478	404	757	O
P22413	Ectonucleotide pyrophosphatase/phosphodiesterase family member 1	ENPP1	13.573	8	4	4	925	104.9	58	66	58	51	54	66	67	102	76	65	69	90	57	84	O
Q13822	Ectonucleotide pyrophosphatase/phosphodiesterase family member 2	ENPP2	79.944	29	26	19	863	98.9	2459	2279	1751	2027	2245	2241	1961	4579	2306	3584	2781	2811	2064	3153	O
Q12805	EGF-containing fibulin-like extracellular matrix protein 1	EFEMP1	134.829	56	55	20	493	54.6	2352	3814	2586	2464	3922	1671	3142	3939	4326	4946	4069	3719	2673	5451	O
Q9H4M9	EH domain-containing protein 1	EHD1	10.995	5	2	2	534	60.6	139	122	121	233	147	144	167	48	76	117	126	83	102	107	O
Q9NZN3	EH domain-containing protein 3	EHD3	14.42	11	4	3	535	60.8	205	131	180	269	264	106	404	13	16	160	110	17	18	67	O
P13804	Electron transfer flavoprotein subunit alpha, mitochondrial	ETFA	10.56	11	3	3	333	35.1	154	159	203	157	207	109	177	157	162	192	194	183	162	158	O
P68104	Elongation factor 1-alpha 1	EEF1A1	11.27	7	3	3	462	50.1	73	32	641	205	64	177	71	34	20	49	38	24	113	40	O
P26641	Elongation factor 1-gamma	EEF1G	6.77	8	3	3	437	50.1	492	207	512	1044	728	684	963	52	11	432	251	50	115	154	O
P13639	Elongation factor 2	EEF2	4.882	2	2	2	858	95.3	12	18	89	113	23	96	20	14	5	17	12	13	21	9	O
P49411	Elongation factor Tu, mitochondrial	TUFM	7.598	5	2	2	452	49.5	72	62	172	100	102	52	72	74	75	87	95	95	73	77	O
Q9NT22	EMILIN-3	EMILIN3	16.851	6	3	3	766	82.6	79	84	94	77	70	73	94	55	125	96	84	133	59	120	O
P17813	Endoglin	ENG	48.679	19	16	10	658	70.5	1541	2205	1743	2019	1714	1008	1906	2087	1866	2778	2565	2841	1318	2984	O
O94919	Endonuclease domain-containing 1 protein	ENDOD1	34.045	23	7	6	500	55	1041	1356	917	916	1468	531	1041	657	739	583	1134	814	522	506	O
Q9NZ08	Endoplasmic reticulum aminopeptidase 1	ERAP1	71.792	21	21	17	941	107.2	1121	1363	663	1859	2800	2512	2130	1742	1154	1992	2354	1881	1719	1519	O
Q6P179	Endoplasmic reticulum aminopeptidase 2	ERAP2	60.666	16	17	14	960	110.4	2565	3510	3175	3552	3757	4542	3401	1736	6490	3885	4004	2566	1176	1490	O
P11021	Endoplasmic reticulum chaperone BiP	HSPA5	149.657	49	43	25	654	72.3	3901	3710	3223	4474	3768	4407	4042	5057	3929	4281	5014	4803	3830	3978	O
Q9BS26	Endoplasmic reticulum resident protein 44	ERP44	39.911	25	11	8	406	46.9	927	775	729	874	880	966	969	1112	836	1012	702	1008	572	735	O
P14625	Endoplasmin	HSP90B1	106.703	40	35	27	803	92.4	5122	3757	4389	2808	3303	4133	4984	4965	2938	3309	2845	3317	3539	4673	O
Q9HCU0	Endosialin	CD248	11.371	5	4	4	757	80.8	450	628	423	275	604	246	341	475	466	502	467	380	273	640	O
Q96AP7	Endothelial cell-selective adhesion molecule	ESAM	12.541	11	3	3	390	41.2	196	324	259	180	386	193	274	213	248	155	227	197	189	203	O
Q9Y5X9	Endothelial lipase	LIPG	10.88	8	4	4	500	56.8	392	214	278	140	207	300	144	140	171	153	163	184	144	127	O
Q9UNN8	Endothelial protein C receptor	PROCR	21.499	17	5	3	238	26.7	290	559	414	315	522	223	365	621	597	538	431	469	319	725	O
P42892	Endothelin-converting enzyme 1	ECE1	4.955	4	2	2	770	87.1	27	49	47	22	37	33	46	37	36	34	38	31	27	41	O
P42126	Enoyl-CoA delta isomerase 1, mitochondrial	ECI1	18.808	21	4	4	302	32.8	284	271	341	440	317	902	557	707	274	320	358	254	276	367	O
P21709	Ephrin type-A receptor 1	EPHA1	19.91	5	4	4	976	108.1	523	573	559	442	734	344	411	594	619	614	616	459	386	873	O
P54764	Ephrin type-A receptor 4	EPHA4	9.53	3	2	2	986	109.8															X
P00533	Epidermal growth factor receptor	EGFR	61.792	14	15	14	1210	134.2	1210	2146	1870	1413	1635	1701	1888	2142	2127	1713	1866	2308	1600	2162	O
Q9Y2E5	Epididymis-specific alpha-mannosidase	MAN2B2	12.264	3	2	2	1009	113.9	48	112	172	76	152	76	130	75	99	233	232	171	88	234	O
P16581	E-selectin	SELE	24.607	11	5	5	610	66.6	391	1770	1059	459	1388	775	389	400	478	379	496	431	324	780	O
P60842	Eukaryotic initiation factor 4A-I	EIF4A1	12.341	7	2	2	406	46.1	27	24	105	116	30	149	36	17	1	18	15	11	43	23	O
P06730	Eukaryotic translation initiation factor 4E	EIF4E	6.626	12	2	2	217	25.1	12	14	15	38	16	52	15	19	5	10	9	7	38	17	O
P63241	Eukaryotic translation initiation factor 5A-1	EIF5A	8.245	23	2	2	154	16.8	151	258	330	391	268	360	376	110	52	129	105	49	286	137	O
Q16394	Exostosin-1	EXT1	26.778	12	7	7	746	86.2	492	476	421	691	525	811	471	1033	534	539	557	638	551	676	O
Q93063	Exostosin-2	EXT2	55.631	26	13	12	718	82.2	742	743	679	823	747	997	710	1384	860	846	825	951	943	1028	O
Q9UBQ6	Exostosin-like 2	EXTL2	26.147	29	6	6	330	37.4	236	218	224	251	218	351	244	305	261	264	242	287	255	287	O
Q16610	Extracellular matrix protein 1	ECM1	187.391	58	132	28	540	60.6	5227	10287	8224	4574	9855	3878	3922	8704	15043	11213	10551	11275	7562	10090	O
O94769	Extracellular matrix protein 2	ECM2	28.927	13	7	6	699	79.7	518	410	449	607	353	486	429	613	505	582	628	530	538	655	O
Q8IXL6	Extracellular serine/threonine protein kinase FAM20C	FAM20C	54.884	25	15	13	584	66.2	1189	1552	1237	1154	1449	1300	1360	1267	1289	1419	1628	1728	1259	2043	O
P08294	Extracellular superoxide dismutase [Cu-Zn]	SOD3	79.699	43	22	11	240	25.8	5038	1212	4864	629	4830	452	709	6891	1032	819	767	899	712	936	O
P15311	Ezrin	EZR	36.143	17	11	6	586	69.4	248	279	294	404	257	452	245	372	285	286	244	204	297	271	O

P52907	F-actin-capping protein subunit alpha-1	CAPZA1	15.292	13	2	2	286	32.9	64	41	82	172	58	210	57	35	10	32	24	21	56	36	O
P47756	F-actin-capping protein subunit beta	CAPZB	6.752	17	3	3	277	31.3	44	42	91	219	44	116	61	50	32	37	22	16	61	58	O
P14324	Farnesyl pyrophosphate synthase	FDPS	7.511	5	2	2	419	48.2	101	116	159	255	127	352	147	137	93	128	121	58	149	141	O
Q01469	Fatty acid-binding protein 5	FABP5	18.473	40	4	4	135	15.2	32	28	285	68	52	39	40	33	19	28	22	27	50	15	O
P15090	Fatty acid-binding protein, adipocyte	FABP4	20.506	38	5	4	132	14.7															X
P05413	Fatty acid-binding protein, heart	FABP3	9.128	24	3	3	133	14.8	197	227	262	167	289	151	191	215	267	243	217	272	232	247	O
P07148	Fatty acid-binding protein, liver	FABP1	7.949	32	4	3	127	14.2	1268	893	1095	659	1820	933	1109	547	1844	1103	1135	1127	594	4191	O
Q96RD9	Fc receptor-like protein 5	FCRL5	57.516	21	15	14	977	106.4	708	1449	1019	882	1025	939	1495	732	853	992	1102	1048	710	1068	O
Q86UX7	Fermitin family homolog 3	FERMT3	58.289	30	18	15	667	75.9	1501	943	1852	2445	1769	8122	2016	298	218	1549	896	417	767	1314	O
P02794	Ferritin heavy chain	FTH1	19.32	34	5	4	183	21.2	186	223	279	172	491	2371	330	1580	384	418	496	914	259	642	O
P02792	Ferritin light chain	FTL	28.424	42	10	7	175	20	594	527	541	531	1221	6128	1079	3886	880	1139	1507	2751	603	1615	O
Q9UGM5	Fetuin-B	FETUB	65.967	35	38	12	382	42	4297	5673	5654	3309	6329	3817	5131	4106	6149	5428	4426	4063	4574	4798	O
P35555	Fibrillin-1	FBN1	143.768	15	29	27	2871	312.1	955	1682	1383	395	1506	724	1143	1052	1506	1380	1293	1377	811	1940	O
P02671	Fibrinogen alpha chain	FGA	197.616	38	99	28	866	94.9	7183	10450	8477	3542	8426	5808	9146	2317	1849	3170	3245	2284	3889	2148	O
P02675	Fibrinogen beta chain	FGB	63.027	41	13	13	491	55.9	857	1965	731	270	819	310	936	1042	389	1174	593	729	554	256	O
P02679	Fibrinogen gamma chain	FGG	29.066	22	9	8	453	51.5	708	1315	555	391	616	370	680	696	323	863	494	521	425	205	O
Q08830	Fibrinogen-like protein 1	FGL1	7.722	7	2	2	312	36.4	95	69	61	40	33	27	87	138	118	178	133	64	168	108	O
P11362	Fibroblast growth factor receptor 1	FGFR1	27.334	11	11	9	822	91.8	1004	1954	1467	764	1659	985	1411	1477	1471	1855	1564	1928	939	2233	O
P21802	Fibroblast growth factor receptor 2	FGFR2	7.868	3	2	2	821	92	126	182	139	87	200	90	119	116	159	154	153	142	88	232	O
P22455	Fibroblast growth factor receptor 4	FGFR4	17.308	5	3	3	802	87.9	108	150	225	75	239	101	99	119	193	138	167	188	102	195	O
Q9BYJ0	Fibroblast growth factor-binding protein 2	FGFBP2	4.022	13	2	2	223	24.6	12	6	19	38	21	17	16	10	24	10	27	13	21	25	O
Q86WI1	Fibrocystin-L	PKHD1L1	110.365	9	24	24	4243	465.4	1815	1414	1466	1898	2088	2272	2463	1434	1504	1817	1529	1400	720	1956	O
Q14314	Fibroleukin	FGL2	26.346	16	10	6	439	50.2	580	1019	703	510	1281	516	590	722	656	774	721	660	609	931	O
P02751	Fibronectin	FN1	1149.483	61	1090	116	2477	272.2	58798	36395	34099	55276	91268	12936	67755	73418	103492	43989	59143	57342	109243	79225	O
P23142	Fibulin-1	FBLN1	192.455	51	90	31	703	77.2	7246	6804	5775	4185	6140	6346	7380	7742	8452	8531	7178	8402	4846	8153	O
P98095	Fibulin-2	FBLN2	7.476	2	2	2	1184	126.5	35	38	34	21	36	20	31	50	42	38	29	34	29	45	O
Q15485	Ficolin-2	FCN2	79.891	40	37	11	313	34	1882	4650	2418	2878	3298	2406	3235	4361	3892	2641	3374	4466	3064	3069	O
O75636	Ficolin-3	FCN3	89.716	53	118	11	299	32.9	4776	9296	8459	11417	8522	12390	7899	11087	9888	10023	7373	8633	10492	10425	O
P21333	Filamin-A	FLNA	240.105	27	55	47	2647	280.6	3667	2104	4669	6272	4353	18341	4886	1008	293	4029	2439	917	1596	4344	O
P30043	Flavin reductase (NADPH)	BLVRB	56.866	60	30	9	206	22.1	902	1868	1647	5977	786	3383	1730	3432	865	738	675	660	4792	1307	O
P14207	Folate receptor beta	FOLR2	29.234	28	5	5	255	29.3	247	519	393	404	510	307	284	422	397	462	372	401	242	512	O
Q12841	Follistatin-related protein 1	FSTL1	44.751	42	16	11	308	35	990	1585	1549	896	1690	884	1164	845	1296	1005	1005	1121	777	1434	O
P04075	Fructose-bisphosphate aldolase A	ALDOA	79.548	58	28	14	364	39.4	1374	2237	2177	4597	2361	4760	1424	3682	1054	1867	1475	1469	2178	2037	O
P05062	Fructose-bisphosphate aldolase B	ALDOB	99.117	55	27	16	364	39.4	2702	1214	2659	3248	3755	10075	3423	3173	1614	1350	1746	1385	2816	3687	O
P09972	Fructose-bisphosphate aldolase C	ALDOC	34.117	29	9	5	364	39.4	103	127	124	174	126	254	83	238	89	123	96	108	112	125	O
P16930	Fumarylacetoacetase	FAH	58.065	43	16	12	419	46.3	825	732	907	1492	1099	3299	1099	1638	1170	591	626	526	834	1037	O
O15117	FYN-binding protein 1	FYB; FYB1	5.201	3	2	2	783	85.3	145	111	129	135	219	165	216	2	7	78	64	18	25	50	O
P09382	Galectin-1	LGALS1	18.673	33	4	4	135	14.7	358	496	452	271	570	376	471	228	483	397	372	412	347	601	O
Q05315	Galectin-10	CLC	9.383	24	3	3	142	16.4	147	145	138	906	214	110	133	86	137	64	76	57	115	47	O
P17931	Galectin-3	LGALS3	11.713	18	4	4	250	26.1	502	622	641	262	523	522	549	641	408	494	514	413	416	488	O
Q08380	Galectin-3-binding protein	LGALS3BP	115.214	48	45	21	585	65.3	2291	2631	2042	1716	2258	1887	2121	3271	7563	3492	2769	2014	1971	2677	O
P47929	Galectin-7	LGALS7; LGALS7B	8.009	20	2	2	136	15.1	36	31	3604	29	35	20	52	18	39	23	33	26	104		O
Q3ZCW2	Galectin-related protein	LGALSL	21.876	36	5	5	172	19	259	116	192	285	296	431	505	5		169	92	23	25	71	O
P09104	Gamma-enolase	ENO2	24.156	29	8	4	434	47.2	34	43	37	75	35	120	43	71	40	38	23	23	65	42	O
Q92820	Gamma-glutamyl hydrolase	GGH	90.164	36	23	12	318	35.9	2265	2511	2445	2077	3294	2720	2676	4114	1908	3602	2325	2859	4005	2572	O
Q9NS71	Gastrokine-1	GKN1	9.62	12	3	2	199	22	339	189	244	305	252	160	182	505	163	220	244	315	191	459	O
O95479	GDH/6PGL endoplasmic bifunctional protein	H6PD	68.546	28	21	18	791	88.8	996	850	732	1527	1236	1416	969	1683	1139	1439	1215	1520	1441	1562	O
Q13630	GDP-L-fucose synthase	TSTA3	5.551	10	2	2	321	35.9	103	157	212	860	96	477	172	373	80	117	80	65	397	136	O
P06396	Gelsolin	GSN	404.808	69	378	46	782	85.6	23368	30034	21748	26969	25140	26696	26716	41069	31382	27061	28055	20845	21663	37953	O
O60234	Glia maturation factor gamma	GMFG	7.657	27	3	3	142	16.8	196	176	260	371	315	395	322	81	71	104	82	76	143	119	O
P07093	Glia-derived nexin	SERPINE2	18.916	16	5	5	398	44	212	491	584	175	453	118	246	13	173	89	240	121	183	133	O
Q6ZMI3	Gliomedin	GLDN	14.58	8	4	4	551	58.9	141	222	148	212	169	154	133	232	248	192	176	199	137	293	O
P11413	Glucose-6-phosphate 1-dehydrogenase	G6PD	16.938	11	6	5	515	59.2	195	186	244	1698	290	863	336	221	109	148	153	139	370	166	O
P06744	Glucose-6-phosphate isomerase	GPI	43.845	23	10	9	558	63.1	650	679	1234	5490	951	4304	812	1487	461	752	529	532	890	751	O
P14314	Glucosidase 2 subunit beta	PRKCSH	33.857	20	9	8	528	59.4	1100	1651	1511	1127	1418	813	1144	1192	1138	1201	1219	1133	1021	1376	O
Q4G148	Glucoside xylosyltransferase 1	GXYLT1	6.155	5	2	2	440	50.5	121	201	172	141	163	181	192	228	177	254	298	243	102	321	O
P48506	Glutamate--cysteine ligase catalytic subunit	GCLC	12.875	8	5	5	637	72.7	344	543	530	1637	469	1359	504	1069	307	269	238	284	1379	508	O
Q07075	Glutamyl aminopeptidase	ENPEP	54.23	17	18	15	957	109.2	1188	1687	1608	1435	1565	2382	2111	2137	1988	1694	1769	2029	1462	1920	O
P35754	Glutaredoxin-1	GLRX	9.657	31	3	3	106	11.8	383	644	686	1288	442	1320	497	504	191	245	202	185	919	454	O
O76003	Glutaredoxin-3	GLRX3	5.043	8	2	2	335	37.4	72	79	113	192	83	195	106	81	34	48	37	18	147	68	O
P36269	Glutathione hydrolase 5 proenzyme	GGT5	7.893	4	2	2	586	62.2															X
P22352	Glutathione peroxidase 3	GPX3	47.328	35	68	7	226	25.5	4294	4484	4514	6357	3151	7508	5100	8692	5533	5018	5034	6063	4638	5174	O
P00390	Glutathione reductase, mitochondrial	GSR	48.285	30	11	9	522	56.2	665	898	880	1115	968	1720	819	2313	894	682	589	882	978	1127	O
P78417	Glutathione S-transferase omega-1	GSTO1	20.085	23	7	6	241	27.5	1188	1041	1076	2470	1251	2791	1318	1579	774	956	736	669	1512	1307	O
P09211	Glutathione S-transferase P	GSTP1	33.287	61	7	7	210	23.3	79	84	194	260	95	337	111	106	17	109	65	38	92	67	O
P48637	Glutathione synthetase	GSS	24.425	23	10	9	474	52.4	511	565	525	628	609	1115	497	1214	454	536	445	436	562	549	O
P04406	Glyceraldehyde-3-phosphate dehydrogenase	GAPDH	47.429	37	12	11	335	36	1002	853	2719	3693	1299	3127	1300	1051	942	1009	924	707	2875	946	O
P21695	Glycerol-3-phosphate dehydrogenase [NAD(+)], cytoplasmic	GPD1	9.522	9	3	3	349	37.5	128	151	171	121	253	339	140	157	159	101	77	87	146	146	O
P11216	Glycogen phosphorylase, brain form	PYGB	17.807	9	6	3	843	96.6															X
P06737	Glycogen phosphorylase, liver form	PYGL	33.875	14	11	8	847	97.1	519	420	761	2477	961	2051	663	523	559	364	340	425	654	571	O
Q9HC38	Glyoxalase domain-containing protein 4	GLOD4	16.521	15	4	4	313	34.8	240	395	441	1067	287	1118	320	688	180	306	209	207	721	350	O
P35052	Glypican-1	GPC1	34.317	22	7	7	558	61.6	216	359	295	629	308	161	296	373	282	249	263	310	215	402	O
Q92896	Golgi apparatus protein 1	GLG1	33.908	9	8	8	1179	134.5	167	369	326	263	417	152	340	113	206	70	184	134	172	115	O
Q8NBJ4	Golgi membrane protein 1	GOLM1	21.503	15	6	6	401	45.3	437	693	740	363	1011	507	396	565	871	813	729	853	486	764	O
Q9H4G4	Golgi-associated plant pathogenesis-related protein 1	GLIPR2	32.968	39	7	5	154	17.2	646	868	696	821	701	939	550	1279	561	715	654	879	644	547	O
Q14393	Growth arrest-specific protein 6	GAS6	17.137	9	5	5	678	74.9	370	400	370	326	485	393	332	502	412	544	427	464	329	595	O
P62993	Growth factor receptor-bound protein 2	GRB2	3.045	8	2	2	217	25.2	131	91	148	316	241	284	250	32	18	139	95	46	59	97	O
P10912	Growth hormone receptor	GHR	24.817	12	8	6	638	71.5	224	276	441	42	515	331	340	115	274	174	172	158	267	158	O
Q9UK05	Growth/differentiation factor 2	GDF2	12.933	8	3	3	429	47.3	150	133	114	16	204	131	90	111	158	171	130	169	122	192	O
O14793	Growth/differentiation factor 8	MSTN	21.406	15	5	4	375	42.7	201	360	478	331	433	284	352	553	302	380	343	501	204	947	O
P62826	GTP-binding nuclear protein Ran	RAN	11.827	19	3	3	216	24.4	114	135	203	377	112	255	177	134	81	49	57	49	410	74	O
Q9Y2T3	Guanine deaminase	GDA	10.696	13	3	3	454	51															X
Q02747	Guanylin	GUCA2A	4.02	17	2	2	115	12.4															X
P00738	Haptoglobin	HP	321.726	68	536	16	406	45.2	34965	6245	1347	2842	5938	3641	1794	3970	2923	6035	3049	8063	10069	2191	O
P00739	Haptoglobin-related protein	HPR	167.639	43	234	3	348	39	407	307	175	203	258	542	263	181	170	409	578	809	277	348	O
P48723	Heat shock 70 kDa protein 13	HSPA13	9.986	6	2	2	471	51.9	34	56	47	29	53	52	51	50	38	54	44	50	35	49	O
P0DMV9	Heat shock 70 kDa protein 1B	HSPA1B; HSPA1A	76.457	33	21	11	641	70	1153	1075	2127	6786	1629	5522	1532	1761	604	963	825	749	1663	897	O
P34932	Heat shock 70 kDa protein 4	HSPA4	19.882	7	4	4	840	94.3	88	136	142	270	152	317	168	264	110	103	108	90	228	123	O
P11142	Heat shock cognate 71 kDa protein	HSPA8	52.407	26	16	9	646	70.9	528	643	1161	2377	733	3490	711	1276	383	673	475	433	1143	710	O
P07900	Heat shock protein HSP 90-alpha	HSP90AA1	33.913	16	12	5	732	84.6	811	887	1777	2013	1242	3109	1033	919	584	865	679	620	1209	960	O
P28906	Hematopoietic progenitor cell antigen CD34	CD34	6.121	5	2	2	385	40.7	33	37	47	57	39	26	44	37	159	77	151	103	67	40	O
Q96RW7	Hemicentin-1	HMCN1	31.222	2	11	11	5635	613	350	467	247	336	463	342	283	377	339	351	325	329	276	390	O
Q8NDA2	Hemicentin-2	HMCN2	27.822	2	7	7	5059	541.6	39	98	44	35	87	40	80	67	81	88	63	63	41	37	O
P69905	Hemoglobin subunit alpha	HBA2; HBA1	65.895	92	40	9	142	15.2	1441	1308	671	7021	574	9553	403	1428	626	564	810	686	2790	637	O
P68871	Hemoglobin subunit beta	HBB	147.219	95	77	7	147	16	2594	1820	974	9658	653	12623	347	1889	234	535	320	652	4847	759	O
P02042	Hemoglobin subunit delta	HBD	78.926	83	32	5	147	16	382	232	157	721	142	1143	136	248	83	136	96	151	735	181	O
P02790	Hemopexin	HPX	414.071	81	1282	45	462	51.6	75049	111967	118950	88537	123349	73144	101578	113530	102874	99802	95192	107650	83791	105844	O
Q9Y251	Heparanase	HPSE	38.528	19	9	9	543	61.1	1684	1226	1927	3014	2850	1130	1899	834	939	374	876	616	719	316	O
P05546	Heparin cofactor 2	SERPIND1	345.346	75	355	38	499	57	25464	18597	17309	26643	18095	28294	19687	21890	19773	21047	18488	21767	21089	24286	O
P11150	Hepatic triacylglycerol lipase	LIPC	23.133	14	5	5	499	55.9	393	372	220	447	362	316	329	274	710	385	272	351	438	442	O
Q8TDQ0	Hepatitis A virus cellular receptor 2	HAVCR2	5.403	14	2	2	301	33.4	114	194	137	90	221	80	108	114	135	162	122	129	90	175	O

Q04756	Hepatocyte growth factor activator	HGFAC	133.753	42	56	22	655	70.6	2253	3806	3732	2287	4035	2549	3239	2820	4155	3160	2840	3537	2959	4091	O
P08581	Hepatocyte growth factor receptor	MET	38.947	10	11	11	1390	155.4	911	1178	876	999	1078	1059	1163	1717	1383	1367	1614	1612	1196	1840	O
P26927	Hepatocyte growth factor-like protein	MST1	207.27	56	103	18	711	80.3	6834	8073	6876	4209	6365	4646	7252	7072	8263	5957	7496	8554	6962	8149	O
P51858	Hepatoma-derived growth factor	HDGF	6.594	10	2	2	240	26.8	126	151	204	261	194	267	131	122	88	135	131	104	166	119	O
Q32P51	Heterogeneous nuclear ribonucleoprotein A1-like 2	HNRNPA1L2	10.964	12	2	2	320	34.2	17	19	72	158	22	82	22	33	18	28	20	7	50	17	O
P22626	Heterogeneous nuclear ribonucleoproteins A2/B1	HNRNPA2B1	13.763	9	2	2	353	37.4	26	29	126	158	60	190	52	49	7	77	49	15	67	22	O
P52790	Hexokinase-3	HK3	6.714	2	2	2	923	99	115	89	98	295	129	176	183	74	119	122	97	67	117	118	O
P04196	Histidine-rich glycoprotein	HRG	258.806	55	406	29	525	59.5	21279	27561	39030	19303	25460	21433	35942	27184	32844	28297	31445	34280	25115	50355	O
P16402	Histone H1.3	HIST1H1D	10.557	14	4	2	221	22.3	112	104	180	1466	233	155	167	120	81	151	140	52	307	72	O
Q16777	Histone H2A type 2-C	HIST2H2AC	11.747	27	4	3	129	14	419	515	910	2346	571	713	502	662	383	424	411	452	664	354	O
Q16778	Histone H2B type 2-E	HIST2H2BE	7.606	19	2	2	126	13.9	351	363	790	1624	323	474	344	369	334	347	278	257	521	279	O
P84243	Histone H3.3	H3F3A; H3F3AP4; H3F3B	3.59	12	3	2	136	15.3	139	126	295	921	190	465	147	283	76	211	141	82	196	82	O
P62805	Histone H4	I2H4B; HIST1H4H; HIST1H4C; HIS	15.789	30	5	4	103	11.4	248	288	747	4882	371	1618	338	1031	119	537	417	165	495	147	O
P04439	HLA class I histocompatibility antigen, A alpha chain	HLA-A	15.866	18	4	2	365	40.8	218	106	107	353	239	765	811	1157	152	454	365	93	216	1367	O
P01903	HLA class II histocompatibility antigen, DR alpha chain	HLA-DRA	16.744	22	4	4	254	28.6	238	222	222	158	792	197	333	614	284	536	284	494	427	1469	O
Q14520	Hyaluronan-binding protein 2	HABP2	116.542	35	76	21	560	62.6	10273	14501	13815	5864	10247	7714	10877	6890	11433	8969	9967	10591	7730	11371	O
Q12794	Hyaluronidase-1	HYAL1	20.64	15	3	3	435	48.3	120	110	116	65	83	134	121	99	99	113	138	116	111	156	O
Q16775	Hydroxyacylglutathione hydrolase, mitochondrial	HAGH	4.179	5	2	2	308	33.8	20	32	28	121	11	96	32	71	13	8	9	8	75	31	O
P00492	Hypoxanthine-guanine phosphoribosyltransferase	HPRT1	9.334	13	2	2	218	24.6	80	151	148	320	95	308	140	262	81	72	81	86	281	144	O
Q9Y4L1	Hypoxia up-regulated protein 1	HYOU1	92.178	22	26	16	999	111.3	2368	4148	3087	1703	3065	1667	2322	2567	3431	2900	2777	3214	1916	3497	O
O75144	ICOS ligand	ICOSLG; LOC102723996	21.109	16	9	4	302	33.3	308	684	489	408	440	258	602	462	646	685	749	690	315	715	O
P55899	IgG receptor FcRn large subunit p51	FCGRT	17.358	8	3	2	365	39.7	207	297	190	172	300	383	268	345	272	243	245	260	229	325	O
Q9Y6R7	IgGFc-binding protein	FCGBP	400.287	28	111	72	5405	571.6	8220	5722	5051	4950	6189	12436	6650	14693	13028	13235	8219	13102	6863	11806	O
P01876	Immunoglobulin heavy constant alpha 1	IGHA1	105.843	54	41	8	353	37.6	729	2354	797	6363	2135	969	3868	258	910	1815	4517	6126	1089	676	O
P01877	Immunoglobulin heavy constant alpha 2	IGHA2	45.06	44	16	5	340	36.6	296	323	160	364	200	140	909	140	77	279	263	405	378	42	O
P01880	Immunoglobulin heavy constant delta	IGHD	24.927	25	8	6	384	42.3	319	161	202	84	412	50	1833	239	12	322	15	737	773	96	O
P01857	Immunoglobulin heavy constant gamma 1	IGHG1	84.083	53	21	6	330	36.1	809	1026	1020	917	2790	1093	1251	524	2775	998	921	1175	860	1178	O
P01859	Immunoglobulin heavy constant gamma 2	IGHG2	43.068	26	14	2	326	35.9	357	411	322	327	948	350	716	146	1069	433	444	413	383	262	O
P01860	Immunoglobulin heavy constant gamma 3	IGHG3	59.383	45	19	5	377	41.3	1329	7465	1482	2205	2073	1000	989	470	1885	1183	1367	2389	348	1778	O
P01861	Immunoglobulin heavy constant gamma 4	IGHG4	37.455	33	12	2	327	35.9	500	715	543	529	2073	1053	1111	290	1689	784	784	961	823	691	O
P01871	Immunoglobulin heavy constant mu	IGHM	106.035	64	46	21	453	49.4	3193	3306	1325	11828	3584	2757	9497	1477	11219	5344	8147	10633	6461	3253	O
A0A0C4DH38	Immunoglobulin heavy variable 5-51	IGHV5-51	21.936	38	3	3	117	12.7	244	410	112	1074	515	349	478	102	976	501	700	620	295	523	O
P01591	Immunoglobulin J chain	IGJ; JCHAIN	13.659	30	5	4	159	18.1	400	510	255	724	536	275	737	295	829	692	728	926	552	475	O
P01834	Immunoglobulin kappa constant	IGKC	51.47	80	30	2	107	11.8	386	560	247	3706	739	451	1175	226	862	502	892	1223	583	416	O
P0DOX7	Immunoglobulin kappa light chain	N/A	44.71	49	27	2	214	23.4	936	1590	765	3699	1924	1026	3238	468	2198	1482	2551	3274	1429	999	O
A0A087WW87	Immunoglobulin kappa variable 2-40	IGKV2-40; IGKV2D-40	14.69	17	3	2	121	13.3	172	255	144	940	304	183	696	93	625	352	446	680	417	195	O
P06312	Immunoglobulin kappa variable 4-1	IGKV4-1	7.554	17	2	2	121	13.4	57	51	31	131	49	22	102	25	125	76	79	91	65	47	O
P0DOY2	Immunoglobulin lambda constant 2	IGLC2	40.078	81	13	3	106	11.3	210	465	141	1098	428	226	490	126	355	356	609	785	322	230	O
A0A075B6I9	Immunoglobulin lambda variable 7-46	IGLV7-46	4.839	14	2	2	117	12.5	29	57	45	181	98	42	115	16	97	167	216	195	72	47	O
B9A064	Immunoglobulin lambda-like polypeptide 5	IGLL5	28.964	39	14	3	214	23	579	1617	480	2408	1208	547	1849	272	1377	1219	2087	2798	1035	814	O
O14498	Immunoglobulin superfamily containing leucine-rich repeat protein	ISLR	27.442	18	6	6	428	46	208	285	255	249	266	218	213	438	276	228	272	265	198	355	O
Q8TDY8	Immunoglobulin superfamily DCC subclass member 4	IGDCC4	21.949	6	5	5	1250	134.1	166	191	141	125	187	137	140	139	188	197	205	216	140	223	O
Q13308	Inactive tyrosine-protein kinase 7	PTK7	20.564	6	5	5	1070	118.3	201	369	277	427	278	132	197	229	187	181	179	174	105	242	O
Q01973	Inactive tyrosine-protein kinase transmembrane receptor ROR1	ROR1	17.272	5	3	3	937	104.2	368	549	447	289	513	239	370	411	490	557	405	482	307	665	O
P55103	Inhibin beta C chain	INHBC	22.668	22	7	6	352	38.2	597	531	615	317	668	367	478	266	566	407	451	452	395	640	O
P14735	Insulin-degrading enzyme	IDE	5.356	2	2	2	1019	117.9	14	10	40	47	17	41	20	31	13	14	16	8	36	19	O
P05019	Insulin-like growth factor I	IGF1	16.9	15	5	3	195	21.8	135	158	180	122	152	118	160	127	160	187	190	153	128	160	O
P01344	Insulin-like growth factor II	IGF2	18.951	24	16	4	180	20.1	737	1036	972	653	955	782	1112	564	1018	897	915	771	822	946	O
P08833	Insulin-like growth factor-binding protein 1	IGFBP1	20.373	20	4	4	259	27.9	796	204	122	50	92	99	478	61	652	1472	1136	649	85	2382	O
P18065	Insulin-like growth factor-binding protein 2	IGFBP2	39.869	28	11	7	325	34.8	979	793	500	318	498	172	600	732	600	1877	1364	1099	587	2162	O
P17936	Insulin-like growth factor-binding protein 3	IGFBP3	90.359	53	47	18	291	31.7	4132	4781	5417	5974	4812	5319	5418	3094	5012	4269	4672	4547	4106	4346	O
P22692	Insulin-like growth factor-binding protein 4	IGFBP4	15.056	32	6	5	258	27.9	531	949	777	418	677	502	537	385	572	623	610	559	486	773	O
P24593	Insulin-like growth factor-binding protein 5	IGFBP5	62.797	43	15	9	272	30.6	1018	1102	978	471	768	541	1154	404	977	1059	1050	743	572	855	O
P24592	Insulin-like growth factor-binding protein 6	IGFBP6	17.952	24	6	4	240	25.3	292	656	443	210	497	266	381	419	386	408	435	454	327	717	O
Q16270	Insulin-like growth factor-binding protein 7	IGFBP7	31.718	32	6	6	282	29.1	619	1112	811	573	1031	571	711	891	732	1077	1039	919	522	1292	O
P35858	Insulin-like growth factor-binding protein complex acid labile subunit	IGFALS	187.17	50	126	26	605	66	10995	8559	10495	14064	8547	17760	11257	16272	12069	11160	10827	12588	13875	10008	O
Q9Y287	Integral membrane protein 2B	ITM2B	4.42	9	2	2	266	30.3	107	198	126	92	136	59	169	92	117	165	147	137	92	152	O
P56199	Integrin alpha-1	ITGA1	11.385	3	4	4	1179	130.8	184	210	220	218	237	259	236	476	249	308	270	293	180	332	O
P17301	Integrin alpha-2	ITGA2	4.339	2	2	2	1181	129.2	13	32	29	32	28	61	37	43	25	31	43	29	24	49	O
P08648	Integrin alpha-5	ITGA5	7.168	3	2	2	1049	114.5	125	158	141	127	199	125	159	316	242	285	223	236	215	255	O
P08514	Integrin alpha-IIb	ITGA2B	15.07	6	5	5	1039	113.3	102	80	118	152	137	163	158	46	53	52	48	27	42	46	O
P20701	Integrin alpha-L	ITGAL	8.383	2	2	2	1170	128.7	134	126	100	164	220	142	140	201	148	185	136	145	161	224	O
P05556	Integrin beta-1	ITGB1	44.018	17	16	12	798	88.4	1593	1909	1555	1892	1879	1534	1880	2457	1948	2362	2120	2251	1331	2590	O
P05107	Integrin beta-2	ITGB2	14.439	5	3	3	769	84.7	72	74	69	72	120	63	73	64	84	93	69	76	69	99	O
P05106	Integrin beta-3	ITGB3	17.636	7	4	4	788	87	235	228	284	347	396	413	362	101	25	111	141	67	92	130	O
Q13418	Integrin-linked protein kinase	ILK	20.909	16	6	6	452	51.4	156	100	231	329	183	1688	169	56	11	91	73	52	113	142	O
P19827	Inter-alpha-trypsin inhibitor heavy chain H1	ITIH1	573.47	55	705	42	911	101.3	58615	48591	43939	30321	39450	47210	51767	65763	57135	58907	52464	52699	55646	25864	O
P19823	Inter-alpha-trypsin inhibitor heavy chain H2	ITIH2	522.202	57	693	54	946	106.4	64768	44180	43159	50366	42973	57336	54467	74848	62492	61531	51702	55481	61268	38134	O
Q06033	Inter-alpha-trypsin inhibitor heavy chain H3	ITIH3	364.121	56	282	36	890	99.8	37562	20809	16851	12301	17976	11402	18977	28228	24884	39328	26384	22561	32166	12167	O
Q14624	Inter-alpha-trypsin inhibitor heavy chain H4	ITIH4	667.703	70	689	59	930	103.3	70738	74573	57099	49428	62920	64440	70788	71605	83599	77135	67251	72479	81467	65614	O
P05362	Intercellular adhesion molecule 1	ICAM1	81.856	31	17	11	532	57.8	1329	1412	1208	473	2176	932	1336	1100	967	1671	1486	1288	685	1954	O
P13598	Intercellular adhesion molecule 2	ICAM2	25.011	21	11	5	275	30.6	1458	3259	2653	1677	3298	1750	2086	2414	2381	3062	3329	2365	1372	3871	O
P32942	Intercellular adhesion molecule 3	ICAM3	22.802	12	9	7	547	59.5	292	556	404	305	584	300	418	496	468	450	483	494	286	530	O
Q9NPH3	Interleukin-1 receptor accessory protein	IL1RAP	40.91	20	21	13	570	65.4	2340	2917	2201	1448	2590	2648	2509	2300	2825	2137	2125	3625	1956	2787	O
P14778	Interleukin-1 receptor type 1	IL1R1	6.813	6	2	2	569	65.4															X
P27930	Interleukin-1 receptor type 2	IL1R2	25.776	21	7	7	398	45.4	609	1119	1128	762	911	884	936	719	1008	977	1128	1212	603	1142	O
P78552	Interleukin-13 receptor subunit alpha-1	IL13RA1	10.298	5	2	2	427	48.7	265	659	601	384	664	433	535	426	410	448	322	461	268	505	O
O95998	Interleukin-18-binding protein	IL18BP	6.469	9	2	2	194	21.1	59	67	50	43	83	17	38	42	55	67	52	40	24	81	O
P08887	Interleukin-6 receptor subunit alpha	IL6R	31.118	25	8	7	468	51.5	187	359	240	294	408	203	297	276	344	332	300	362	287	397	O
P40189	Interleukin-6 receptor subunit beta	IL6ST	39.106	12	11	9	918	103.5	377	698	631	484	696	468	572	809	620	729	652	678	522	888	O
O75874	Isocitrate dehydrogenase [NADP] cytoplasmic	IDH1	34.294	23	10	8	414	46.6	680	529	767	1861	1358	3127	767	1426	632	657	539	580	825	1076	O
P48735	Isocitrate dehydrogenase [NADP], mitochondrial	IDH2	14.211	9	5	4	452	50.9	351	321	657	551	625	341	451	348	430	377	479	467	403	488	O
P29622	Kallistatin	SERPINA4	298.829	64	233	25	427	48.5	18587	9086	17108	23156	14640	20605	18295	25044	23898	21435	22052	21194	18831	25227	O
P01042	Kininogen-1	KNG1	505.588	53	714	45	644	71.9	56142	88331	73301	39989	70390	39783	57707	34748	51514	59081	62876	58650	37461	75370	O
P21583	Kit ligand	KITLG	4.224	7	2	2	273	30.9															X
O43278	Kunitz-type protease inhibitor 1	SPINT1	13.815	6	3	3	529	58.4	290	369	317	187	327	161	276	289	359	326	278	275	229	402	O
P02788	Lactotransferrin	LTF	222.745	67	49	40	710	78.1	5280	9350	5779	3609	16759	4562	9236	4740	4630	3141	2729	3038	6275	1822	O
Q04760	Lactoylglutathione lyase	GLO1	7.748	14	3	3	184	20.8	8	17	16	32	13	26	12	26	12	10	7	4	23	17	O
P20700	Lamin-B1	LMNB1	9.619	6	3	3	586	66.4	22	37	30	30	35	21	22	36	27	30	20	18	10	39	O
P24043	Laminin subunit alpha-2	LAMA2	131.649	12	29	28	3122	343.7	2519	2713	2354	2317	2203	2095	2080	2950	2517	2765	2310	2766	2024	3249	O
P07942	Laminin subunit beta-1	LAMB1	99.023	18	28	24	1786	197.9	2219	2155	1815	2186	2044	2022	2127	2765	2484	2671	2184	2723	1686	2648	O
P55268	Laminin subunit beta-2	LAMB2	34.045	6	7	7	1798	195.9	320	376	309	285	299	306	296	352	365	366	366	379	268	480	O
P11047	Laminin subunit gamma-1	LAMC1	54.543	14	17	17	1609	177.5	1886	2041	1900	1665	1530	1827	1320	2093	1537	1858	1452	1678	1329	1808	O
Q14766	Latent-transforming growth factor beta-binding protein 1	LTBP1	164.935	27	41	30	1721	186.7	4391	5196	5516	3777	7574	3531	5862	2341	4136	1537	4027	2745	3878	1640	O
P48357	Leptin receptor	LEPROT; LEPR	11.61	4	3	3	1165	132.4	120	132	151	88	69	84	142	171	177	170	174	142	98	194	O
P02750	Leucine-rich alpha-2-glycoprotein	LRG1	122.178	65	86	19	347	38.2	8374	8196	6611	4546	7215	4543	4647	10725	6832	8139	8720	7249	6792	5025	O
Q6UXK5	Leucine-rich repeat neuronal protein 1	LRRN1	3.767	3	2	2	716	80.7	279	364	300	329	361	522	429	498	432	405	379	356	379	498	O
O14960	Leukocyte cell-derived chemotaxin-2	LECT2	5.367	11	2	2	151	16.4	515	711	783	662	928	639	642	343	523	466	530	542	539	525	O
P30740	Leukocyte elastase inhibitor	SERPINB1	34.013	25	8	7	379	42.7	660	542	646	2146	1066	1514	1073	594	304	304	303	335	623	450	O
O75023	Leukocyte immunoglobulin-like receptor subfamily B member 5	LILRB5	23.612	18	7	7	590	64.1	409	630	394	349	329	385	486	474	559	555	586	461	391	456	O
P09960	Leukotriene A-4 hydrolase	LTA4H	96.753	42	24	21	611	69.2	3412	2186	2212	6842	2310	4457	2282	5071	1882	2449	1927	2129	2420	3796	O

P48059	LIM and senescent cell antigen-like-containing domain protein 1	LIMS1	6.496	8	2	2	325	37.2	52	30	79	90	65	523	69	15	14	45	33	12	31	52	O
Q14847	LIM and SH3 domain protein 1	LASP1	18.31	20	5	5	261	29.7	723	579	1425	1271	1306	1424	1174	176	105	447	332	163	344	344	O
Q13449	Limbic system-associated membrane protein	LSAMP	29.4	24	9	7	338	37.4	341	523	438	265	357	274	439	420	513	590	511	551	311	582	O
Q86X29	Lipolysis-stimulated lipoprotein receptor	LSR	3.61	4	2	2	649	71.4															X
P18428	Lipopolysaccharide-binding protein	LBP	112.836	33	49	13	481	53.4	12554	7885	8269	6714	7925	4620	8948	3326	4169	10731	9201	7330	8655	6731	O
P06858	Lipoprotein lipase	LPL	10.068	5	2	2	475	53.1	67	213	113	51	62	121	79	110	31	122	68	42	57	124	O
P05451	Lithostathine-1-alpha	REG1A	14.556	31	3	3	166	18.7	349	227	425	152	248	195	155	154	98	659	455	197	120	342	O
P48304	Lithostathine-1-beta	REG1B	17.737	30	3	3	166	18.7															X
P23141	Liver carboxylesterase 1	CES1	73.609	35	16	15	567	62.5	983	914	883	4131	1440	3333	923	1300	1325	826	669	929	2399	1168	O
Q969E1	Liver-expressed antimicrobial peptide 2	LEAP2	4.283	26	2	2	77	8.8															X
P00338	L-lactate dehydrogenase A chain	LDHA	18.962	17	6	4	332	36.7	376	469	510	797	526	1587	396	477	218	314	223	315	385	462	O
P07195	L-lactate dehydrogenase B chain	LDHB	62.256	39	22	10	334	36.6	2280	3196	2675	5095	2822	6999	2780	6080	2467	3272	2439	2969	3356	3633	O
P12318	Low affinity immunoglobulin gamma Fc region receptor II-a	FCGR2A	12.086	9	4	2	317	35	160	201	163	92	297	133	139	138	194	286	209	178	144	245	O
O75015	Low affinity immunoglobulin gamma Fc region receptor III-B	FCGR3B	18.901	20	10	2	233	26.2	1471	2491	4140	1090	3298	1513	3001	2266	1804	1793	1524	2226	2488	1930	O
P01130	Low-density lipoprotein receptor	LDLR	6.912	3	2	2	860	95.3	129	68	56	74	56	125	199	60	152	58	33	61	88	85	O
P14151	L-selectin	SELL	52.882	19	21	7	372	42.2	1455	3018	1964	1385	3266	1585	2574	2295	2726	2513	2457	2252	1893	2840	O
P51884	Lumican	LUM	139.266	43	162	14	338	38.4	15548	30078	18376	17313	37071	15913	18716	22002	18473	26606	21766	18903	11839	31340	O
Q9Y5Y7	Lymphatic vessel endothelial hyaluronic acid receptor 1	LYVE1	40.803	21	20	7	322	35.2	1647	3024	2290	1037	3255	1451	1303	1561	2311	2809	2546	2632	1192	3114	O
Q5SQ64	Lymphocyte antigen 6 complex locus protein G6f	LY6G6F	8.768	14	3	2	297	32.4	137	129	398	78	167	74	207	7	6	36	32	12	16	8	O
P19256	Lymphocyte function-associated antigen 3	CD58	5.206	10	2	2	250	28.1	20	31	19	19	27	16	21	28	22	28	22	19	13	42	O
P33241	Lymphocyte-specific protein 1	LSP1	13.224	10	2	2	339	37.2	82	62	95	386	136	174	107	39	18	52	46	28	73	25	O
P04062	Lysosomal acid glucosylceramidase	GBA	9.014	7	2	2	536	59.7	305	280	246	224	461	229	322	255	315	221	266	244	250	257	O
O00754	Lysosomal alpha-mannosidase	MAN2B1	25.675	6	4	4	1011	113.7	255	251	262	265	333	277	286	196	196	178	186	149	164	186	O
P10619	Lysosomal protective protein	CTSA	23.377	13	4	4	480	54.4	1632	906	1144	1799	1836	852	1794	820	805	400	696	536	646	534	O
P42785	Lysosomal Pro-X carboxypeptidase	PRCP	19.43	11	5	4	496	55.8	216	279	269	344	294	400	324	285	304	258	271	274	228	338	O
P11279	Lysosome-associated membrane glycoprotein 1	LAMP1	21.472	12	6	5	417	44.9	731	1395	1444	849	1209	881	1240	978	1237	928	1234	1027	620	1331	O
P13473	Lysosome-associated membrane glycoprotein 2	LAMP2	23.605	15	13	6	410	44.9	448	867	740	498	844	537	747	467	785	689	694	568	469	712	O
P61626	Lysozyme C	LYZ	34.219	57	16	7	148	16.5	1816	1399	1679	1613	3340	990	2372	1615	1814	2026	1962	2254	1870	2823	O
P58215	Lysyl oxidase homolog 3	LOXL3	37.942	12	7	7	753	83.1	221	270	388	209	357	249	370	147	116	76	182	162	200	111	O
P09603	Macrophage colony-stimulating factor 1	CSF1	9.195	7	5	4	554	60.1	503	770	741	421	819	263	499	434	571	615	565	587	413	744	O
P07333	Macrophage colony-stimulating factor 1 receptor	CSF1R	57.775	12	15	9	972	107.9	1039	1500	1179	606	1875	1318	1178	1301	1422	1760	1353	1546	936	1959	O
P22897	Macrophage mannose receptor 1	MRC1	179.36	30	46	36	1456	165.9	4733	5743	5241	2932	6122	4517	3953	5612	3999	5502	5013	5072	3566	6278	O
Q9UEW3	Macrophage receptor MARCO	MARCO	12.265	5	2	2	520	52.6	133	175	130	82	290	117	167	53	195	263	232	246	165	195	O
P40925	Malate dehydrogenase, cytoplasmic	MDH1	22.723	20	6	6	334	36.4	306	386	476	1269	443	1142	370	897	244	330	323	322	569	472	O
P40926	Malate dehydrogenase, mitochondrial	MDH2	42.015	34	9	9	338	35.5	685	690	1280	1055	1026	581	711	807	730	806	848	973	784	742	O
P48740	Mannan-binding lectin serine protease 1	MASP1	184.863	43	71	24	699	79.2	3963	4986	4842	6608	4055	6192	5198	6964	6782	6673	6149	6549	5033	6125	O
O00187	Mannan-binding lectin serine protease 2	MASP2	171.402	45	51	26	686	75.7	2375	2797	2614	2749	2794	3397	2308	4560	2941	3113	2483	3096	3397	2760	O
P11226	Mannose-binding protein C	MBL2	81.314	57	37	13	248	26.1	5586	4687	6062	3003	2144	856	1303	3338	5086	4632	2290	4042	752	4962	O
P33908	Mannosyl-oligosaccharide 1,2-alpha-mannosidase IA	MAN1A1	116.343	31	39	19	653	72.9	4069	3562	3648	3588	3546	3949	4679	5244	4630	4010	3985	4396	4227	4676	O
Q9H8J5	MANSC domain-containing protein 1	MANSC1	7.96	6	3	2	431	46.8	313	473	349	274	456	189	300	448	356	441	452	355	290	508	O
P10721	Mast/stem cell growth factor receptor Kit	KIT	34.324	10	14	9	976	109.8	2762	3599	3116	2328	3125	1882	1773	4135	2411	3802	3336	3387	2059	4534	O
Q99542	Matrix metalloproteinase-19	MMP19	13.205	7	2	2	508	57.3	102	107	82	89	107	83	93	88	105	110	92	108	76	90	O
P14780	Matrix metalloproteinase-9	MMP9	103.378	41	32	21	707	78.4	3272	2574	2059	6490	3221	822	2859	2532	1549	1377	1224	1278	3314	673	O
O95866	Megakaryocyte and platelet inhibitory receptor G6b	C6orf25; MPIG6B	16.805	15	3	3	241	26.1	1260	1786	6780	653	1626	653	1572	7	40	594	438	76	95	101	O
P40967	Melanocyte protein PMEL	PMEL	9.798	9	3	3	661	70.2															X
P15529	Membrane cofactor protein	CD46	3.598	5	2	2	392	43.7	201	375	610	200	350	145	232	175	168	242	209	170	109	309	O
Q16853	Membrane primary amine oxidase	AOC3	38.867	17	10	9	763	84.6	608	439	423	743	534	748	647	1145	700	910	707	770	556	776	O
P55145	Mesencephalic astrocyte-derived neurotrophic factor	MANF	11.37	23	3	3	182	20.7															X
Q13421	Mesothelin	MSLN	15.546	9	4	4	630	68.9	277	350	322	200	164	216	403	146	207	474	326	228	135	705	O
P01033	Metalloproteinase inhibitor 1	TIMP1	43.952	53	11	7	207	23.2	564	1146	954	610	1239	514	785	480	667	458	728	639	529	637	O
P16035	Metalloproteinase inhibitor 2	TIMP2	28.235	36	9	8	220	24.4	1593	1277	1198	731	1226	708	1020	1382	870	1070	1085	1128	803	1568	O
Q13228	Methanethiol oxidase	SELENBP1	102.319	45	21	17	472	52.4	1349	1998	2044	4422	1874	3005	2302	5693	1835	860	820	1145	4834	2322	O
P55083	Microfibril-associated glycoprotein 4	MFAP4	20.691	20	5	3	255	28.6	46	36	36	27	72	8	72	109	73	84	75	94	84	118	O
Q15691	Microtubule-associated protein RP/EB family member 1	MAPRE1	11.173	14	4	3	268	30	398	252	547	952	597	1342	706	144		432	294	78	264	228	O
Q15555	Microtubule-associated protein RP/EB family member 2	MAPRE2	12.125	11	3	2	327	37	195	95	166	228	333	458	436	5		88	66	23	24	60	O
P20774	Mimecan	OGN	21.323	23	6	5	298	33.9	458	399	345	352	687	401	346	587	480	420	360	508	448	639	O
P26038	Moesin	MSN	103.997	44	26	17	577	67.8	877	967	1007	2100	1153	2533	1027	1638	676	1073	826	843	1045	971	O
P08571	Monocyte differentiation antigen CD14	CD14	88.633	39	37	13	375	40.1	2571	3555	2803	2064	2596	2295	2729	2820	2924	3092	3501	3258	2586	3467	O
Q13201	Multimerin-1	MMRN1	149.302	36	49	34	1228	138	4563	6677	7081	4640	7953	5321	7047	3070	3920	1228	3515	2690	4046	1842	O
Q9H8L6	Multimerin-2	MMRN2	33.876	6	8	5	949	104.3	1173	1425	1028	1480	1176	682	1514	1460	1369	1513	1189	1457	1242	1573	O
Q7Z7M0	Multiple epidermal growth factor-like domains protein 8	MEGF8	99.985	9	23	21	2845	302.9	1459	1527	1418	2367	1535	3316	1914	2416	2316	1714	1910	2327	1818	2100	O
Q9H1U4	Multiple epidermal growth factor-like domains protein 9	MEGF9	8.254	5	3	3	602	62.9	226	464	323	153	392	231	286	259	305	301	369	361	227	346	O
Q9UNW1	Multiple inositol polyphosphate phosphatase 1	MINPP1	101.584	40	34	19	487	55	3019	3755	3468	3076	3504	2654	3974	3334	3852	3213	3519	3524	3049	5202	O
P41218	Myeloid cell nuclear differentiation antigen	MNDA	4.124	6	2	2	407	45.8	60	40	70	953	94	311	89	41	4	27	35	34	53	32	O
P05164	Myeloperoxidase	MPO	90.516	32	23	19	745	83.8	973	1156	680	8652	2224	970	2615	1350	1103	600	530	532	814	455	O
Q99972	Myocilin	MYOC	34.312	24	9	9	504	56.9	751	1192	589	1047	909	1019	733	970	1151	1031	1122	1220	610	1381	O
P02144	Myoglobin	MB	9.151	19	2	2	154	17.2	189	190	133	200	601	316	189	394	99	239	179	168	131	315	O
P05976	Myosin light chain 1/3, skeletal muscle isoform	MYL1	9.714	16	4	2	194	21.1	123	128	181	163	206	86	157	121	181	105	144	158	150	195	O
P08590	Myosin light chain 3	MYL3	39.199	41	12	6	195	21.9	894	903	1405	1129	1535	691	1080	894	1158	952	1136	1318	1137	1269	O
P12829	Myosin light chain 4	MYL4	10.63	18	3	2	197	21.6															X
P60660	Myosin light polypeptide 6	MYL6	17.501	30	7	3	151	16.9	661	487	1550	1764	1018	3173	858	136	52	507	347	286	711	1510	O
O14950	Myosin regulatory light chain 12B	MYL12B; MYL12A	4.98	12	2	2	172	19.8	37	35	50	64	47	70	39	32	19	29	28	23	31	47	O
P10916	Myosin regulatory light chain 2, ventricular/cardiac muscle isoform	MYL2	22.431	41	8	7	166	18.8	934	790	1027	901	1167	484	786	852	981	978	787	1042	1061	869	O
P13533	Myosin-6	MYH6	293.819	35	77	19	1939	223.6	6728	5766	8169	7608	8696	4152	6861	6180	7504	6759	6902	7625	7889	7760	O
P12883	Myosin-7	MYH7	236.292	27	61	4	1935	223	454	349	664	487	656	348	552	415	533	362	522	569	513	607	O
P35579	Myosin-9	MYH9	182.614	24	42	39	1960	226.4	1528	1183	4130	3115	2253	8596	2094	688	407	1326	934	845	1794	3517	O
P58546	Myotrophin	LUZP6; MTPN	6.627	27	3	3	118	12.9	134	144	232	262	268	351	262	60	12	123	66	35	108	95	O
P20933	N(4)-(beta-N-acetylglucosaminyl)-L-asparaginase	AGA	26.381	22	4	4	346	37.2	137	195	266	30	308	227	272	163	260	200	241	249	221	347	O
P34059	N-acetylgalactosamine-6-sulfatase	GALNS	6.611	5	2	2	522	58	92	75	79	49	43	83	93	104	75	183	156	106	57	231	O
Q86SF2	N-acetylgalactosaminyltransferase 7	GALNT7	22.644	8	4	4	657	75.3	392	445	348	524	460	657	370	840	477	575	467	561	452	487	O
Q9UK23	N-acetylglucosamine-1-phosphodiester alpha-N-acetylglucosaminidase	NAGPA	11.871	6	4	4	515	56	219	400	294	169	368	272	297	269	335	321	310	398	234	444	O
Q9UJJ9	N-acetylglucosamine-1-phosphotransferase subunit gamma	GNPTG	45.641	28	8	6	305	34	1061	1034	1073	774	690	757	998	681	1148	1027	991	1073	819	1316	O
Q9NY97	N-acetyllactosaminide beta-1,3-N-acetylglucosaminyltransferase 2	B3GNT2	51.475	34	16	12	397	46	1468	1739	1705	1352	1720	1287	2004	2084	1978	2159	2037	1763	1490	2195	O
Q8N0V5	N-acetyllactosaminide beta-1,6-N-acetylglucosaminyl-transferase	GCNT2	11.528	9	3	3	402	45.8	109	125	113	95	99	106	140	119	111	103	131	134	89	178	O
Q96PD5	N-acetylmuramoyl-L-alanine amidase	PGLYRP2	210.485	60	103	21	576	62.2	3625	4242	4167	4843	4535	4156	3849	7107	4898	5553	5128	5226	4545	7471	O
P28331	NADH-ubiquinone oxidoreductase 75 kDa subunit, mitochondrial	NDUFS1	3.417	3	2	2	727	79.4	60	66	62	51	70	50	70	71	84	75	72	84	63	82	O
Q15223	Nectin-1	PVRL1; NECTIN1	43.328	25	8	8	517	57.1	199	361	274	172	399	237	237	303	300	284	314	321	193	307	O
Q92692	Nectin-2	PVRL2; NECTIN2	4.979	4	2	2	538	57.7															X
Q9NQS3	Nectin-3	PVRL3; NECTIN3	5.438	6	2	2	549	61	24	28	29	23	41	22	22	27	30	36	26	26	15	43	O
Q92859	Neogenin	NEO1	57.695	16	18	14	1461	159.9	786	1065	824	1067	1091	1123	1115	1737	1276	1600	1259	1485	831	1561	O
P08473	Neprilysin	MME	16.69	7	4	4	750	85.5	122	123	117	172	132	280	305	103	125	95	93	89	106	102	O
Q9UMX5	Neudesin	NENF	7.231	12	2	2	172	18.8															X
P13591	Neural cell adhesion molecule 1	NCAM1	107.559	34	47	22	858	94.5	4539	6217	5884	3636	4992	3446	6147	6499	5847	6971	6977	7623	3695	7833	O
O15394	Neural cell adhesion molecule 2	NCAM2	69.942	25	21	18	837	93	1055	1389	1445	1066	1088	1131	1213	1527	1342	1681	1859	1806	1049	1974	O
P32004	Neural cell adhesion molecule L1	L1CAM	31.075	9	9	9	1257	139.9	656	993	948	545	711	556	530	970	979	740	647	654	480	826	O
O00533	Neural cell adhesion molecule L1-like protein	CHL1	138.702	31	46	28	1208	135	4232	4475	4080	3239	4467	4109	4676	5218	4940	6060	5191	6707	3782	6241	O
Q9Y4C0	Neurexin-3	NRXN3	10.034	2	2	2	1643	180.5	145	103	104	100	73	141	154	176	161	143	142	186	102	187	O
Q09666	Neuroblast differentiation-associated protein AHNAK	AHNAK	8.846	3	3	3	5890	628.7	253	276	622	121	304	172	222	170	291	249	186	199	292	325	O
P41271	Neuroblastoma suppressor of tumorigenicity 1	BL1; C1orf151-NBL1; MINOS1-NBl	7.881	10	3	2	181	19.4	151	230	144	126	199	109	143	151	174	177	149	160	110	232	O
O14594	Neurocan core protein	NCAN	10.294	3	2	2	1321	143	49	30	25	25	23	24	31	29	44	32	36	44	15	45	O
P46531	Neurogenic locus notch homolog protein 1	NOTCH1	11.689	1	3	3	2555	272.3	319	434	382	120	420	263	317	354	344	347	329	366	263	594	O

Q04721	Neurogenic locus notch homolog protein 2	NOTCH2	36.939	5	9	9	2471	265.2	735	1119	937	598	1130	642	979	810	1123	1117	1090	1121	650	1394	O
Q9UM47	Neurogenic locus notch homolog protein 3	NOTCH3	11.851	1	3	3	2321	243.5															X
Q92823	Neuronal cell adhesion molecule	NRCAM	28.995	8	6	6	1304	143.8	357	633	498	364	559	515	596	649	630	596	541	664	352	530	O
Q7Z3B1	Neuronal growth regulator 1	NEGR1	16.536	15	4	4	354	38.7	379	680	474	300	650	380	435	501	570	596	521	622	348	881	O
O14786	Neuropilin-1	NRP1	112.301	29	33	22	923	103.1	3383	3587	2784	2422	3617	3097	4150	5012	3461	5294	4121	4934	2225	4355	O
O60462	Neuropilin-2	NRP2	24.569	11	10	10	931	104.8	422	365	267	315	424	363	331	452	433	356	325	435	271	473	O
P30990	Neurotensin/neuromedin N	NTS	11.725	29	2	2	170	19.8															X
Q14697	Neutral alpha-glucosidase AB	GANAB	13.776	6	5	5	944	106.8	346	243	291	376	264	263	293	301	266	294	246	188	336	311	O
P22894	Neutrophil collagenase	MMP8	19.23	12	4	4	467	53.4	263	344	272	1793	769	142	476	277	253	94	35	81	279	65	O
P59665	Neutrophil defensin 1	DEFA1; DEFA1B	14.46	20	13	4	94	10.2	722	618	710	1370	1351	1057	931	1105	530	673	761	836	1023	771	O
P80188	Neutrophil gelatinase-associated lipocalin	LCN2	27.508	43	11	6	198	22.6	489	1083	761	2006	1471	419	962	591	611	238	236	443	810	510	O
Q6GTS8	N-fatty-acyl-amino acid synthase/hydrolase PM20D1	PM20D1	23.284	13	5	5	502	55.7	90	5	29	1154	1725	71	3455	19	38	1478	348	1025	1422	988	O
P14543	Nidogen-1	NID1	119.917	27	31	24	1247	136.3	3060	2213	2032	1585	2856	2139	2431	1658	1307	971	1436	1444	2080	1540	O
Q14112	Nidogen-2	NID2	43.465	11	12	11	1375	151.2	979	998	1072	1081	1869	1149	934	1072	801	365	830	619	900	431	O
Q9GZT8	NIF3-like protein 1	NIF3L1	27.511	19	6	6	377	41.9	389	537	508	491	532	844	483	1032	524	474	424	458	521	490	O
Q5JPE7	Nodal modulator 2	NOMO2	40.106	13	12	11	1267	139.4	1237	1279	1115	1260	1209	1305	1532	1173	1385	1442	1311	1593	1169	1545	O
Q99784	Noelin	OLFM1	48.511	19	16	9	485	55.3	1610	1815	1335	1403	1955	2304	2060	2383	2098	2012	1859	2490	1510	2411	O
O95897	Noelin-2	OLFM2	23.809	15	7	7	454	51.4	189	288	161	318	157	254	130	274	214	338	381	313	275	706	O
P10153	Non-secretory ribonuclease	RNASE2	14.24	20	4	3	161	18.3	88	237	161	123	371	87	224	131	264	178	147	147	153	201	O
P61916	NPC intracellular cholesterol transporter 2	NPC2	13.646	33	4	3	151	16.6	554	820	665	686	946	402	619	632	654	580	594	652	477	925	O
Q16288	NT-3 growth factor receptor	NTRK3	10.748	5	4	4	839	94.4	142	267	206	149	195	145	193	246	217	273	269	244	158	263	O
Q02818	Nucleobindin-1	NUCB1	73.861	38	14	14	461	53.8	941	1167	1146	648	898	714	1110	469	789	859	845	766	578	1418	O
P80303	Nucleobindin-2	NUCB2	4.82	4	2	2	420	50.2	82	106	104	78	109	78	99	79	72	43	68	61	70	44	O
P15531	Nucleoside diphosphate kinase A	NME1	21.379	48	7	3	152	17.1	235	332	452	1521	284	1270	358	733	90	202	190	148	889	356	O
P22392	Nucleoside diphosphate kinase B	NME2; NME1-NME2	19.139	49	6	2	152	17.3	166	94	119	258	90	661	101	184	30	68	71	52	154	91	O
P55209	Nucleosome assembly protein 1-like 1	NAP1L1	10.026	7	2	2	391	45.3		4	8	19	4	82	12	10		3	2	3	13	5	O
Q9H173	Nucleotide exchange factor SIL1	SIL1	6.179	6	2	2	461	52.1	101	103	93	110	84	72	81	85	73	114	93	70	88	81	O
Q6UWY5	Olfactomedin-like protein 1	OLFML1	6.615	5	2	2	402	45.9															X
Q8WWZ8	Oncoprotein-induced transcript 3 protein	OIT3	29.972	19	9	9	545	60	1274	991	886	726	1188	639	820	595	1002	852	879	898	741	1131	O
Q99650	Oncostatin-M-specific receptor subunit beta	OSMR	12.214	3	2	2	979	110.4	264	322	264	140	275	136	269	327	279	211	254	277	185	284	O
Q14982	Opioid-binding protein/cell adhesion molecule	OPCML	11.807	8	2	2	345	38	60	80	70	35	59	45	62	64	79	75	74	76	40	130	O
Q8IYS5	Osteoclast-associated immunoglobulin-like receptor	OSCAR	8.096	11	3	2	282	30.5	52	80	49	32	73	40	61	33	60	61	72	62	58	107	O
Q99983	Osteomodulin	OMD	16.144	13	4	4	421	49.5	564	792	547	414	954	570	661	901	1134	821	610	676	644	1084	O
P10451	Osteopontin	SPP1	18.375	15	3	3	314	35.4	310	352	378	134	430	136	264	293	285	285	386	429	297	619	O
Q86UD1	Out at first protein homolog	OAF	34.684	45	12	9	273	30.7	1705	1870	1585	1267	1991	1177	1502	1307	1821	1672	1647	1821	1159	2379	O
P04746	Pancreatic alpha-amylase	AMY2A	72.903	32	20	2	511	57.7	577	438	382	452	481	680	842	1616	480	393	340	566	679	599	O
P16233	Pancreatic triacylglycerol lipase	PNLIP	8.103	6	2	2	465	51.1	309	208	164	225	175	194	283	826	234	179	122	222	257	159	O
O95497	Pantetheinase	VNN1	51.946	27	18	10	513	57	1663	691	478	425	2159	3611	717	1413	1195	454	677	838	1293	1141	O
O95428	Papilin	PAPLN	32.962	8	7	7	1278	137.6	182	283	210	138	194	115	209	177	250	225	236	148	179	253	O
Q99497	Parkinson disease protein 7	PARK7	25.012	34	6	5	189	19.9	186	187	195	416	237	271	328	207	145	186	148	67	255	188	O
O00151	PDZ and LIM domain protein 1	PDLIM1	29.93	27	5	5	329	36	427	175	478	592	654	1422	941	43	3	294	186	70	114	276	O
Q6UXB8	Peptidase inhibitor 16	PI16	85.284	29	39	12	463	49.4	3053	6794	4280	2803	7103	2965	2844	4561	4041	5057	3875	5326	2938	6547	O
P19021	Peptidyl-glycine alpha-amidating monooxygenase	PAM	61.276	21	16	16	973	108.3	1360	1342	1149	1137	1235	1170	1385	1764	1699	1659	1524	1527	1221	1727	O
P62937	Peptidyl-prolyl cis-trans isomerase A	PPIA	40.4	69	13	10	165	18	776	608	1058	3825	1150	3153	1152	1032	144	763	505	266	1040	543	O
P23284	Peptidyl-prolyl cis-trans isomerase B	PPIB	35.409	40	11	10	216	23.7	850	920	1035	1424	1117	847	1007	672	425	680	954	723	636	505	O
P45877	Peptidyl-prolyl cis-trans isomerase C	PPIC	5.67	10	2	2	212	22.7	324	426	365	359	377	311	292	463	347	335	341	331	339	480	O
P62942	Peptidyl-prolyl cis-trans isomerase FKBP1A	FKBP1A	11.45	26	2	2	108	11.9															X
Q15063	Periostin	POSTN	103.773	36	23	20	836	93.3	2313	729	1451	1353	1628	2387	1601	4059	2070	2326	1938	2538	1821	2694	O
Q06830	Peroxiredoxin-1	PRDX1	36.984	45	9	8	199	22.1	809	1169	1904	3885	975	3450	1590	1781	673	739	590	610	3825	1367	O
P32119	Peroxiredoxin-2	PRDX2	57.692	41	26	11	198	21.9	1449	3754	3041	9371	1769	6414	3669	4664	1422	1691	1271	1151	10888	2975	O
P30044	Peroxiredoxin-5, mitochondrial	PRDX5	9.634	19	3	3	214	22.1	506	367	753	807	766	997	945	135	204	355	283	213	322	264	O
P30041	Peroxiredoxin-6	PRDX6	33.471	43	10	7	224	25	505	760	1088	2436	547	1871	1090	817	444	613	443	372	2560	705	O
P04180	Phosphatidylcholine-sterol acyltransferase	LCAT	63.111	43	30	12	440	49.5	1135	795	736	247	753	144	1320	333	1250	915	779	812	1001	947	O
P30086	Phosphatidylethanolamine-binding protein 1	PEBP1	39.305	48	7	7	187	21	512	680	868	1422	884	1798	923	726	266	368	285	307	1007	594	O
Q96S96	Phosphatidylethanolamine-binding protein 4	PEBP4	37.162	48	7	7	227	25.7	884	940	586	952	1577	828	792	1248	468	1067	730	1165	736	1376	O
P80108	Phosphatidylinositol-glycan-specific phospholipase D	GPLD1	238.088	53	111	31	840	92.3	17623	14639	12278	8385	12093	14107	17804	13471	15170	21108	20692	18094	16547	17117	O
P36871	Phosphoglucomutase-1	PGM1	8.113	6	3	3	562	61.4	89	73	97	139	152	248	85	78	46	45	40	45	75	75	O
P00558	Phosphoglycerate kinase 1	PGK1	85.626	49	20	15	417	44.6	810	913	1451	3940	1058	5516	1163	1361	183	600	558	519	1665	827	O
P18669	Phosphoglycerate mutase 1	PGAM1; LOC643576	18.861	18	5	4	254	28.8	443	430	785	2183	515	1735	570	904	436	469	347	374	636	477	O
Q53H76	Phospholipase A1 member A	PLA1A	14.246	8	2	2	456	49.7	61	40	45	56	40	42	35	44	56	51	48	44	60	42	O
Q8NCC3	Phospholipase A2 group XV	PLA2G15	20.528	14	4	4	412	46.6	117	120	117	127	180	146	130	118	100	173	112	115	69	162	O
P55058	Phospholipid transfer protein	PLTP	90.502	27	24	13	493	54.7	2993	2889	3090	4417	4026	3891	3908	2402	3316	3688	4311	4144	2266	3856	O
P36955	Pigment epithelium-derived factor	SERPINF1	183.15	56	133	21	418	46.3	13153	12154	11475	15084	15335	20631	13696	23831	18634	13231	13771	16336	15517	17647	O
Q9BTY2	Plasma alpha-L-fucosidase	FUCA2	42.738	27	17	12	467	54	2387	3395	2740	2708	2463	4159	2864	4244	2505	3523	3756	3684	2417	4440	O
P03952	Plasma kallikrein	KLKB1	350.853	73	223	43	638	71.3	20941	28429	23992	11480	22552	20832	27066	11776	17311	15746	15614	20785	15733	24601	O
P05155	Plasma protease C1 inhibitor	SERPING1	248.145	44	316	25	500	55.1	60143	86901	81809	8411	63681	21548	55757	73188	27922	56819	52327	58743	34137	71956	O
P05154	Plasma serine protease inhibitor	SERPINA5	109.963	41	42	16	406	45.6	3860	8499	6380	1659	5868	433	6150	440	4450	3483	4699	3579	1822	7745	O
P00747	Plasminogen	PLG	670.614	78	757	75	810	90.5	49614	45595	45516	39032	43879	38044	46698	46930	43748	50279	54719	54583	44177	54908	O
P05121	Plasminogen activator inhibitor 1	SERPINE1	29.607	25	8	8	402	45	249	452	587	527	500	651	440	463	259	123	293	264	356	199	O
P13796	Plastin-2	LCP1	237.743	70	80	30	627	70.2	6044	5942	5434	10445	6413	7003	4567	11331	5853	6983	5116	6484	5068	6055	O
P13797	Plastin-3	PLS3	59.14	23	20	7	630	70.8	165	251	219	310	305	363	208	405	168	228	189	210	216	321	O
P02775	Platelet basic protein	PPBP	70.566	44	77	9	128	13.9	8293	14474	14709	9929	18110	7530	12890	4684	9041	3998	11530	5840	7151	5036	O
P16284	Platelet endothelial cell adhesion molecule	PECAM1	51.014	17	11	9	738	82.5	920	2094	1511	864	1518	1230	1619	1727	1007	1538	1627	1161	719	1718	O
P02776	Platelet factor 4	PF4	54.492	45	39	4	101	10.8	9014	13601	14247	8619	17579	4944	13763	3060	8549	3041	7342	4799	7158	4028	O
P10720	Platelet factor 4 variant	PF4V1	46.318	58	30	4	104	11.5	1396	2738	2390	1436	3064	1011	2786	448	788	529	1727	836	1178	629	O
P16671	Platelet glycoprotein 4	CD36	4.658	4	2	2	472	53															X
P07359	Platelet glycoprotein Ib alpha chain	GP1BA	69.422	14	25	9	652	71.5	2324	2276	2527	2412	3243	1241	3414	1440	1772	1615	1787	1819	1283	1379	O
P40197	Platelet glycoprotein V	GP5	108.503	34	32	14	560	60.9	4268	4296	2504	6814	5911	5624	5639	2276	2110	929	2386	1786	2019	1453	O
Q9HCN6	Platelet glycoprotein VI	GP6	20.482	17	7	5	339	36.8	1720	2019	3473	1022	2082	1292	1865	62	136	399	416	218	198	236	O
Q13093	Platelet-activating factor acetylhydrolase	PLA2G7	46.005	31	11	10	441	50	895	1003	1059	2353	1336	2229	2113	2286	1236	1379	792	902	2023	1198	O
P09619	Platelet-derived growth factor receptor beta	PDGFRB	39.411	9	8	8	1106	123.9	696	476	394	583	407	278	774	1461	1360	689	532	428	234	510	O
P04085	Platelet-derived growth factor subunit A	PDGFA	6.928	18	2	2	211	24															X
P08567	Pleckstrin	PLEK	26.494	28	5	5	350	40.1	233	101	146	186	160	556	308	24	21	197	131	29	50	115	O
Q8IUK5	Plexin domain-containing protein 1	PLXDC1	23.758	12	6	5	500	55.7	341	609	454	421	566	346	548	626	483	635	464	642	322	608	O
Q6UX71	Plexin domain-containing protein 2	PLXDC2	45.263	22	14	10	529	59.5	1179	1760	1482	1201	2022	1322	1539	1583	1381	1585	1440	1529	857	1664	O
Q9UIW2	Plexin-A1	PLXNA1	26.014	6	7	7	1896	210.9	335	429	328	344	442	400	418	643	450	460	379	423	369	596	O
O43157	Plexin-B1	PLXNB1	24.31	4	8	7	2135	232.2	405	351	376	425	366	447	403	667	519	453	485	519	398	645	O
O15031	Plexin-B2	PLXNB2	84.703	13	15	15	1838	205	710	706	975	526	919	700	930	898	820	936	793	757	765	955	O
Q9Y4D7	Plexin-D1	PLXND1	39.144	7	8	8	1925	211.9	317	520	434	327	494	353	485	524	466	592	515	464	291	640	O
O00592	Podocalyxin	PODXL	22.197	9	7	5	558	58.6	526	1099	839	640	993	638	718	609	887	1028	1060	1024	519	1185	O
P15151	Poliovirus receptor	PVR	19.761	12	12	5	417	45.3	816	1368	1774	1115	1331	1202	1302	1466	1413	1782	2008	1904	1096	2516	O
P01833	Polymeric immunoglobulin receptor	PIGR	31.213	18	10	10	764	83.2	896	1322	804	284	994	387	712	405	355	469	496	313	384	1746	O
Q10472	Polypeptide N-acetylgalactosaminyltransferase 1	GALNT1	15.403	6	3	3	559	64.2	262	267	248	443	287	367	277	473	252	267	328	314	242	293	O
Q10471	Polypeptide N-acetylgalactosaminyltransferase 2	GALNT2	36.366	19	10	8	571	64.7	918	1058	901	1088	1089	1083	1026	1405	1042	992	1063	982	1003	976	O
Q7Z7M9	Polypeptide N-acetylgalactosaminyltransferase 5	GALNT5	18.091	6	4	4	940	106.2	583	293	460	464	617	484	748	891	472	846	756	613	633	784	O
Q8NCL4	Polypeptide N-acetylgalactosaminyltransferase 6	GALNT6	8.772	5	2	2	622	71.1	33	43	41	45	43	33	33	45	47	42	36	50	40	44	O
P08397	Porphobilinogen deaminase	HMBS	10.217	14	3	3	361	39.3	129	188	224	768	91	558	191	365	114	93	66	53	486	156	O
P20742	Pregnancy zone protein	PZP	224.1	31	83	28	1482	163.8	16335	20612	1437	2686	13314	206	14279	386	1617	4244	1451	6896	1680	388	O
P02545	Prelamin-A/C	LMNA	7.006	5	3	3	664	74.1	25	42	204	35	52	22	35	28	38	36	27	22	33	30	O
Q9UHG3	Prenylcysteine oxidase 1	PCYOX1	113.067	48	43	21	505	56.6	6689	4312	4847	14664	2994	13214	6194	4300	10394	4189	3292	3282	6049	4132	O
Q9H3G5	Probable serine carboxypeptidase CPVL	CPVL	6.072	5	2	2	476	54.1	36	60	50	40	58	37	38	36	46	28	37	35	42	45	O

P09668	Pro-cathepsin H	CTSH	16.377	18	6	5	335	37.4	523	412	537	411	231	772	713	747	538	382	293	500	452	617	O
Q15113	Procollagen C-endopeptidase enhancer 1	PCOLCE	94.652	55	29	17	449	47.9	2102	3167	2230	1851	2931	1818	1980	2764	2929	2379	2370	2370	1906	3731	O
P07737	Profilin-1	PFN1	84.533	71	21	10	140	15	2548	1376	3023	8531	4141	12783	4890	1057	71	2929	1754	470	1170	1361	O
Q9BQ51	Programmed cell death 1 ligand 2	PDCD1LG2	10.007	9	3	2	273	30.9	435	921	879	502	806	484	540	694	618	899	710	709	454	1072	O
Q8WUM4	Programmed cell death 6-interacting protein	PDCD6IP	4.189	2	3	2	868	96	193	257	413	668	266	625	287	306	175	199	157	160	377	240	O
O75340	Programmed cell death protein 6	PDCD6	5.035	13	2	2	191	21.9	47	50	47	62	50	63	54	85	50	48	51	34	45	59	O
Q9UQ80	Proliferation-associated protein 2G4	PA2G4	9.888	9	3	3	394	43.8	36	73	122	23	64	333	77	69	20	45	33	24	106	61	O
Q96NZ9	Proline-rich acidic protein 1	PRAP1	20.223	25	4	2	151	17.2	309	596	659	299	721	526	387	374	695	871	589	581	528	668	O
Q07954	Prolow-density lipoprotein receptor-related protein 1	LRP1	194.174	13	52	46	4544	504.3	5234	5227	4798	6446	4990	6346	5249	10423	6621	6808	5915	6882	5101	7458	O
P48147	Prolyl endopeptidase	PREP	6.252	4	2	2	710	80.6	24	30	61	55	36	84	24	63	25	26	24	24	39	38	O
Q12884	Prolyl endopeptidase FAP	FAP	56.51	16	16	11	760	87.7	493	1182	451	1378	1129	1360	630	1692	926	992	824	900	982	969	O
P27918	Properdin	CFP	104.743	34	55	13	469	51.2	6392	5846	5603	3981	7356	3909	6728	4309	7152	6300	6509	7882	4836	7995	O
P29122	Proprotein convertase subtilisin/kexin type 6	PCSK6	13.957	5	4	4	969	106.4	507	635	579	725	635	667	772	382	384	258	440	320	403	238	O
Q8NBP7	Proprotein convertase subtilisin/kexin type 9	PCSK9	73.771	26	21	14	692	74.2	4209	3057	3394	2218	2808	3654	2465	3028	2271	2834	1946	2395	2628	3459	O
P07602	Prosaposin	PSAP	33.137	24	12	10	524	58.1	2889	2692	2679	1993	3087	1262	3026	732	2246	1699	1913	1634	1658	2023	O
Q15185	Prostaglandin E synthase 3	PTGES3	9.354	13	2	2	160	18.7	72	70	172	242	130	262	186	86	46	85	55	34	108	69	O
P41222	Prostaglandin-H2 D-isomerase	PTGDS	35.275	38	10	6	190	21	744	1183	749	388	1125	612	776	612	945	870	884	1048	589	993	O
Q06323	Proteasome activator complex subunit 1	PSME1	18.302	21	6	5	249	28.7	323	437	595	1142	443	923	513	394	373	239	262	205	921	421	O
Q9UL46	Proteasome activator complex subunit 2	PSME2	9.342	12	2	2	239	27.4	177	303	310	595	313	481	247	208	227	172	176	169	374	319	O
Q92530	Proteasome inhibitor PI31 subunit	PSMF1	9.963	10	2	2	271	29.8	32	41	37	111	28	68	33	41	20	31	24	22	71	26	O
P25786	Proteasome subunit alpha type-1	PSMA1	13.645	25	5	5	263	29.5	358	485	804	1445	658	1959	381	1321	280	398	343	385	642	499	O
P25787	Proteasome subunit alpha type-2	PSMA2	13.89	14	2	2	234	25.9	44	63	102	144	86	240	46	155	43	45	33	40	81	63	O
P25788	Proteasome subunit alpha type-3	PSMA3	11.384	15	4	4	255	28.4	197	229	430	685	338	1074	200	679	173	214	151	167	383	287	O
P25789	Proteasome subunit alpha type-4	PSMA4	19.835	25	5	5	261	29.5	315	344	639	1143	524	1482	303	1025	243	299	199	257	546	414	O
P28066	Proteasome subunit alpha type-5	PSMA5	18.422	22	4	4	241	26.4	286	339	694	1086	530	1526	312	984	312	337	212	274	617	462	O
P60900	Proteasome subunit alpha type-6	PSMA6	17.692	25	5	5	246	27.4	61	88	186	360	123	495	61	342	98	88	49	85	145	114	O
O14818	Proteasome subunit alpha type-7	PSMA7	23.297	28	6	6	248	27.9	277	313	532	704	432	913	294	707	246	273	231	253	412	351	O
P20618	Proteasome subunit beta type-1	PSMB1	16.889	18	5	3	241	26.5	78	107	198	393	143	574	73	361	48	61	53	72	154	104	O
P49720	Proteasome subunit beta type-3	PSMB3	12.525	20	3	3	205	22.9	67	111	197	317	151	479	83	195	70	66	61	28	170	86	O
P28070	Proteasome subunit beta type-4	PSMB4	14.684	14	4	3	264	29.2	182	416	651	997	743	2184	485	872	204	314	296	251	565	512	O
P28072	Proteasome subunit beta type-6	PSMB6	13.263	14	3	3	239	25.3	146	238	432	575	284	823	176	605	157	188	102	142	380	253	O
P02760	Protein AMBP	AMBP	318.319	58	438	23	352	39	20563	25753	23758	17209	23496	18322	21219	28319	24052	23951	21421	23310	23556	20190	O
O60888	Protein CutA	CUTA	11.942	26	5	3	179	19.1	398	754	617	729	634	654	590	897	647	656	606	691	531	759	O
O60610	Protein diaphanous homolog 1	DIAPH1	8.407	2	2	2	1272	141.3	103	65	123	304	91	361	74	56	9	84	53	27	119	88	O
Q96HD1	Protein disulfide isomerase CRELD1	CRELD1	23.378	13	5	4	420	45.4	297	559	521	206	459	263	440	271	436	366	432	405	281	461	O
P07237	Protein disulfide-isomerase	P4HB	53.336	31	19	17	508	57.1	2667	2225	2092	2487	1835	2380	1891	2534	2068	2129	1741	1819	2171	1625	O
P30101	Protein disulfide-isomerase A3	PDIA3	67.484	39	16	16	505	56.7	1049	952	1042	1681	935	1378	948	1597	856	1134	1070	1057	1060	1179	O
P13667	Protein disulfide-isomerase A4	PDIA4	29.38	14	6	6	645	72.9	249	260	237	200	303	259	260	343	217	272	224	254	250	263	O
Q15084	Protein disulfide-isomerase A6	PDIA6	15.761	10	3	3	440	48.1	264	193	315	358	235	283	268	207	170	292	260	197	187	255	O
P49257	Protein ERGIC-53	LMAN1	13.766	6	3	2	510	57.5	141	219	275	99	110	84	237	314	519	127	103	112	141	133	O
Q92520	Protein FAM3C	FAM3C; WNT16	33.82	37	8	7	227	24.7	1036	1179	1263	975	1193	1254	976	1362	1093	1090	1081	1163	810	1105	O
Q6P4E1	Protein GOLM2	GOLM2	8.765	6	2	2	436	49.5	19	35	31	26	45	29	22	33	42	29	35	28	36	28	O
Q9ULI3	Protein HEG homolog 1	HEG1	74.818	16	19	16	1381	147.4	1560	2723	2237	1187	2606	1335	1717	1803	1757	2239	2127	2131	1247	2817	O
P78504	Protein jagged-1	JAG1	13.804	5	4	4	1218	133.7	176	289	240	202	244	160	241	252	246	271	253	243	142	307	O
Q99435	Protein kinase C-binding protein NELL2	NELL2	4.678	2	2	2	816	91.3	100	90	105	96	83	101	86	128	121	109	113	118	76	208	O
Q9BUN1	Protein MENT	C1orf56	21.016	14	5	4	341	36.7	44	41	56	202	32	61	42	103	286	175	287	187	47	38	O
Q8WZA1	Protein O-linked-mannose beta-1,2-N-acetylglucosaminyltransferase 1	POMGNT1	25.996	16	8	7	660	75.2	277	278	280	295	228	349	483	574	422	468	424	470	359	449	O
P31949	Protein S100-A11	S100A11	9.888	26	2	2	105	11.7	127	127	724	1016	225	301	213	136	98	85	85	76	146	74	O
P80511	Protein S100-A12	S100A12	4.169	16	2	2	92	10.6	242	266	187	3366	558	1222	512	266	90	68	120	147	304	57	O
P26447	Protein S100-A4	S100A4	4.539	17	2	2	101	11.7	122	192	146	324	128	370	158	121	60	119	99	81	281	125	O
P06703	Protein S100-A6	S100A6	9.773	27	7	4	90	10.2	351	787	637	1855	435	1217	587	637	149	309	296	234	1095	383	O
P31151	Protein S100-A7	S100A7	25.283	35	5	4	101	11.5	481	528	1836	199	590	410	525	447	562	1070	301	937	1354	902	O
P05109	Protein S100-A8	S100A8	48.75	63	27	12	93	10.8	1808	1762	1835	9153	3146	5205	2424	1816	780	638	807	983	2169	464	O
P06702	Protein S100-A9	S100A9	75.738	89	31	12	114	13.2	3007	3636	2969	11551	5168	4616	4875	1737	1901	1179	1272	1342	2877	1057	O
Q7Z5N4	Protein sidekick-1	SDK1	8.26	2	3	3	2213	242	269	317	250	189	258	295	194	359	313	373	267	293	245	284	O
Q9UK55	Protein Z-dependent protease inhibitor	SERPINA10	144.175	56	55	23	444	50.7	4240	3790	2602	3838	2788	3582	2975	3655	3252	4054	3026	4195	4123	3891	O
P22061	Protein-L-isoaspartate(D-aspartate) O-methyltransferase	PCMT1	11.927	12	2	2	227	24.6	107	102	103	217	105	208	138	111	82	94	68	75	184	95	O
Q92954	Proteoglycan 4	PRG4	118.793	21	52	25	1404	151	7319	4775	5453	5861	8382	5975	6153	2729	9613	4164	4377	4860	5689	5801	O
P00734	Prothrombin	F2	609.481	68	658	50	622	70	40137	43516	39933	32164	32978	21695	50408	38763	50887	34821	39014	38664	33210	25592	O
Q14517	Protocadherin Fat 1	FAT1	24.755	2	8	8	4588	506	384	329	542	375	614	710	307	269	346	270	327	287	347	421	O
Q6V0I7	Protocadherin Fat 4	FAT4	17.038	1	4	4	4981	542.4	298	332	271	293	318	270	353	329	407	407	342	361	263	432	O
Q9NPG4	Protocadherin-12	PCDH12	51.946	13	11	10	1184	128.9	1626	2029	1597	1517	2337	1657	1991	1625	2202	2020	1991	1892	1550	2192	O
Q9HCL0	Protocadherin-18	PCDH18	10.082	3	3	3	1135	126.1	724	952	575	653	933	523	698	753	919	807	685	851	556	1168	O
Q9HC56	Protocadherin-9	PCDH9	9.741	3	5	5	1237	136	364	368	394	287	623	422	444	278	587	715	478	382	229	428	O
P16109	P-selectin	SELP	83.401	28	19	18	830	90.8	2291	3155	6191	1861	3034	1581	1921	566	776	787	1097	764	660	643	O
Q8IWL2	Pulmonary surfactant-associated protein A1	SFTPA1	3.776	9	2	2	248	26.2	245	181	138	222	135	111	182	330	284	276	278	284	229	500	O
P35247	Pulmonary surfactant-associated protein D	SFTPD	13.508	13	4	3	375	37.7	427	153	236	413	655	171	266	278	515	685	306	1179	321	604	O
P00491	Purine nucleoside phosphorylase	PNP	43.344	52	11	11	289	32.1	512	861	983	4106	574	4354	883	2424	222	554	545	387	2108	785	O
P55786	Puromycin-sensitive aminopeptidase	NPEPPS	18.484	6	6	5	919	103.2	148	132	211	344	139	397	150	216	94	141	109	102	215	138	O
Q8IZP2	Putative protein FAM10A4	ST13P4	8.963	13	5	3	240	27.4	354	598	708	1195	585	957	760	468	280	439	332	199	1052	465	O
Q9HBR0	Putative sodium-coupled neutral amino acid transporter 10	SLC38A10	38.766	8	7	7	1119	119.7	82	133	112	66	133	74	86	111	117	145	122	131	75	165	O
P11177	Pyruvate dehydrogenase E1 component subunit beta, mitochondrial	PDHB	11.054	8	2	2	359	39.2	199	177	289	207	250	152	203	208	191	186	199	187	213	192	O
P30613	Pyruvate kinase PKLR	PKLR	4.787	4	2	2	574	61.8	15	10	15	58	15	30	18	29	18	8	6	5	53	14	O
P14618	Pyruvate kinase PKM	PKM	84.558	50	22	20	531	57.9	1811	2080	3089	4072	2719	5962	2070	1356	874	1696	1432	1209	1535	1790	O
Q08257	Quinone oxidoreductase	CRYZ	19.96	19	4	4	329	35.2	267	178	315	548	490	1214	323	439	281	175	206	220	367	473	O
P31150	Rab GDP dissociation inhibitor alpha	GDI1	39.344	23	8	4	447	50.6	85	103	93	182	92	347	111	193	80	122	101	73	127	139	O
P50395	Rab GDP dissociation inhibitor beta	GDI2	65.284	42	15	11	445	50.6	644	977	936	4389	830	3663	1093	2096	497	751	601	606	2049	974	O
Q15404	Ras suppressor protein 1	RSU1	14.809	20	4	4	277	31.5	1030	437	693	1273	780	4361	734	269	407	1139	608	413	971	643	O
P62491	Ras-related protein Rab-11A	RAB11A	14.811	15	3	3	216	24.4	203	97	209	119	264	201	332	20	26	114	78	49	51	100	O
O00194	Ras-related protein Rab-27B	RAB27B	13.785	17	3	3	218	24.6	545	213	406	397	640	513	978	3	5	318	176	12	47	176	O
Q9NRW1	Ras-related protein Rab-6B	RAB6B	6.829	11	2	2	208	23.4	607	405	570	652	619	553	984	159	195	424	306	208	254	364	O
P51149	Ras-related protein Rab-7a	RAB7A	13.511	22	3	3	207	23.5	199	117	221	563	252	345	354	42	32	147	95	52	72	105	O
P61224	Ras-related protein Rap-1b	RAP1B	19.724	29	6	5	184	20.8	364	182	329	510	279	558	345	62	57	328	209	91	153	295	O
P23467	Receptor-type tyrosine-protein phosphatase beta	PTPRB	33.781	6	9	8	1997	224.2	542	952	698	668	710	601	622	811	563	692	741	687	404	756	O
P08575	Receptor-type tyrosine-protein phosphatase C	PTPRC	30.317	6	8	7	1306	147.4	637	1064	816	597	1346	546	739	838	954	3151	953	5212	689	1091	O
P23468	Receptor-type tyrosine-protein phosphatase delta	PTPRD	14.276	2	3	2	1912	214.6	172	139	122	142	146	169	144	265	161	161	99	159	138	147	O
Q12913	Receptor-type tyrosine-protein phosphatase eta	PTPRJ	38.473	9	12	10	1337	145.9	741	1578	937	1082	1360	831	1335	912	1537	1232	1762	1158	849	1589	O
P10586	Receptor-type tyrosine-protein phosphatase F	PTPRF	127.757	19	26	23	1907	212.7	1792	1534	1422	1671	1520	1701	1729	2656	2125	2062	1882	1876	1890	2138	O
P23470	Receptor-type tyrosine-protein phosphatase gamma	PTPRG	33.602	6	9	7	1445	161.9	679	1163	937	734	1119	697	816	1194	1148	1212	1006	1256	743	1391	O
Q15262	Receptor-type tyrosine-protein phosphatase kappa	PTPRK	11.756	3	3	3	1439	162															X
P28827	Receptor-type tyrosine-protein phosphatase mu	PTPRM	65.772	13	14	12	1452	163.6	647	1132	879	746	858	795	791	1101	885	1170	1103	943	579	1332	O
Q13332	Receptor-type tyrosine-protein phosphatase S	PTPRS	71.363	12	15	13	1948	216.9	1713	1833	1113	1254	1646	1470	1394	2697	1908	1983	1561	1904	1145	1963	O
P23471	Receptor-type tyrosine-protein phosphatase zeta	PTPRZ1	42.513	5	13	11	2315	254.4	1104	1072	1030	752	966	856	1191	1148	1080	1249	1387	1636	978	1260	O
P78509	Reelin	RELN	89.982	7	19	19	3460	388.1	1858	1167	1044	1355	1580	1735	1364	1554	1204	1376	1475	1643	927	1493	O
Q06141	Regenerating islet-derived protein 3-alpha	REG3A	16.158	19	2	2	175	19.4	359	243	280	135	200	138	206	191	97	479	336	181	173	164	O
Q96B86	Repulsive guidance molecule A	RGMA	11.65	11	2	2	450	49.3	61	135	105	68	176	84	95	113	92	95	99	119	76	195	O
Q15293	Reticulocalbin-1	RCN1	18.764	19	6	5	331	38.9	445	562	545	397	335	340	363	350	423	489	537	381	385	499	O
Q9BZR6	Reticulon-4 receptor	RTN4R	3.974	3	2	2	473	50.7	142	144	141	135	231	205	167	253	163	131	127	129	151	270	O
Q86UN3	Reticulon-4 receptor-like 2	RTN4RL2	15.225	11	5	4	420	46.1	346	433	368	278	405	532	409	414	375	280	255	320	294	470	O
P00352	Retinal dehydrogenase 1	ALDH1A1	63.36	30	16	11	501	54.8	733	492	716	2003	1274	2122	703	724	548	385	250	221	1504	804	O
Q99969	Retinoic acid receptor responder protein 2	RARRES2	31.142	55	9	7	163	18.6	983	917	779	642	1185	552	721	605	863	699	629	671	1244	1125	O

P02753	Retinol-binding protein 4	RBP4	270.364	79	612	18	201	23	38325	51341	51812	28597	34602	30588	40686	45902	49401	36479	41031	39826	35965	65216	O
O95980	Reversion-inducing cysteine-rich protein with Kazal motifs	RECK	39.358	14	10	10	971	106.4	679	1048	798	352	840	455	892	680	946	1282	1082	1317	519	1297	O
P52565	Rho GDP-dissociation inhibitor 1	ARHGDIA	13.293	19	4	3	204	23.2	311	274	491	1118	431	1250	475	280	62	443	258	128	438	279	O
P52566	Rho GDP-dissociation inhibitor 2	ARHGDIB	20.223	39	8	8	201	23	446	403	662	1818	829	1724	872	361	201	487	323	167	437	280	O
Q07960	Rho GTPase-activating protein 1	ARHGAP1	9.465	7	2	2	439	50.4	210	202	181	308	160	413	138	105	76	197	123	118	259	130	O
P34096	Ribonuclease 4	RNASE4	29.244	54	11	8	147	16.8	1464	1531	1874	1314	1714	1252	1413	1204	1325	1587	1771	1918	1372	1994	O
P13489	Ribonuclease inhibitor	RNH1	60.798	41	13	11	461	49.9	294	511	467	594	419	416	384	501	343	278	261	235	594	434	O
P07998	Ribonuclease pancreatic	RNASE1	15.739	30	4	2	156	17.6	446	812	635	196	616	313	431	595	555	647	628	702	417	879	O
O00584	Ribonuclease T2	RNASET2	18.36	21	7	5	256	29.5	874	1600	1741	1641	2234	802	1737	1290	1610	1454	1616	1503	1073	1561	O
P49247	Ribose-5-phosphate isomerase	LOC101060545; RPIA	9.64	10	2	2	311	33.2	43	77	56	167	63	193	84	234	39	24	48	58	216	66	O
Q9Y6N7	Roundabout homolog 1	ROBO1	13.674	2	2	2	1651	180.8	31	44	29	28	32	34	42	45	47	40	33	42	25	42	O
Q8WZ75	Roundabout homolog 4	ROBO4	34.027	12	11	8	1007	107.4	324	1021	566	424	587	359	384	721	608	873	763	550	265	1164	O
Q86TD4	Sarcalumenin	SRL	7.327	3	3	3	932	100.7	228	332	301	333	406	183	249	271	305	273	288	319	269	379	O
P16615	Sarcoplasmic/endoplasmic reticulum calcium ATPase 2	ATP2A2	28.834	9	10	8	1042	114.7	383	350	509	415	540	245	408	392	433	389	404	458	404	416	O
Q6ZMJ2	Scavenger receptor class A member 5	SCARA5	10.594	5	2	2	495	54	29	55	51	26	48	26	34	31	38	39	31	42	17	46	O
Q86VB7	Scavenger receptor cysteine-rich type 1 protein M130	CD163	173.244	37	52	28	1156	125.4	2269	2666	2242	1254	4087	1480	1819	1654	2515	3838	3490	2889	1479	3319	O
Q13103	Secreted phosphoprotein 24	SPP2	27.089	27	11	6	211	24.3	792	759	754	424	459	558	774	575	1020	711	761	866	491	578	O
P05060	Secretogranin-1	CHGB	20.404	6	3	3	677	78.2	170	238	168	79	73	70	140	175	181	119	166	245	69	110	O
Q8WXD2	Secretogranin-3	SCG3	3.539	4	2	2	468	53	131	135	126	52	97	93	114	98	147	139	143	148	68	152	O
Q9BYH1	Seizure 6-like protein	SEZ6L	4.065	3	2	2	1024	111.7	27	30	31	20	25	21	27	31	33	39	38	27	22	33	O
P49908	Selenoprotein P	SEPP1; SELENOP	46.303	18	28	9	381	43.2	3820	4782	5151	4342	4300	3380	4282	4202	4999	4450	4833	5070	2991	7276	O
Q14563	Semaphorin-3A	SEMA3A	10.482	5	3	3	771	88.8	96	126	84	76	92	87	135	92	107	108	105	107	105	136	O
Q13275	Semaphorin-3F	SEMA3F	13.736	9	4	4	785	88.3	63	59	47	54	78	63	65	55	55	60	53	61	45	77	O
Q9NPR2	Semaphorin-4B	SEMA4B	25.397	9	9	8	837	92.7	1217	1064	1034	983	1518	1403	1514	1569	1009	1498	1329	1150	1377	1495	O
Q92854	Semaphorin-4D	SEMA4D	14.549	6	3	3	862	96.1	442	648	590	769	773	612	646	284	198	189	276	228	226	211	O
O75326	Semaphorin-7A	SEMA7A	24.615	11	5	5	666	74.8	239	319	350	342	351	285	429	420	383	402	309	305	305	429	O
P10124	Serglycin	SRGN	37.287	24	14	4	158	17.6	1954	2597	2287	790	3721	869	2397	441	1592	514	1688	977	859	629	O
Q92743	Serine protease HTRA1	HTRA1	8.96	6	3	3	480	51.3	416	379	367	173	501	320	355	106	268	304	286	249	283	299	O
P00995	Serine protease inhibitor Kazal-type 1	SPINK1	11.777	43	3	3	79	8.5	134	125	125	57	149	52	103	145	94	137	134	130	89	180	O
Q9NQ38	Serine protease inhibitor Kazal-type 5	SPINK5	49.625	17	15	14	1064	120.6	613	1347	731	170	503	236	659	536	673	619	688	590	410	718	O
Q15257	Serine/threonine-protein phosphatase 2A activator	PPP2R4; PTPA	4.549	6	2	2	358	40.6	96	188	166	736	135	497	219	272	96	114	106	44	498	211	O
P62136	Serine/threonine-protein phosphatase PP1-alpha catalytic subunit	PPP1CA	7.373	9	2	2	330	37.5	10	15	9	26	11	40	7	22	2	14	11	9	12	7	O
P02787	Serotransferrin	TF	343.83	61	165	45	698	77	13203	13738	30324	15823	23608	11453	12351	1081	17381	12221	14339	9794	14280	8377	O
Q86U17	Serpin A11	SERPINA11	30.81	19	9	8	422	47	1310	973	1486	490	853	900	900	448	745	514	537	740	971	1107	O
P48594	Serpin B4	SERPINB4	4.122	10	2	2	390	44.8	24	32	239	11	20	18	26	59	14	22	1	25	175	19	O
P0DJI8	Serum amyloid A-1 protein	SAA1	29.41	65	9	3	122	13.5	762	201	178	134	149	158	156	313	196	222	190	230	335	213	O
P35542	Serum amyloid A-4 protein	SAA4	21.539	45	17	5	130	14.7	1487	739	898	1930	995	2503	1185	923	1009	502	1236	1011	1329	623	O
P02743	Serum amyloid P-component	APCS	89.404	34	95	12	223	25.4	10924	7612	6481	4373	6519	12770	7901	12464	5844	7602	8121	8574	9414	7677	O
P27169	Serum paraoxonase/arylesterase 1	PON1	160.962	69	52	13	355	39.7	8784	7718	5245	9339	5681	4751	7597	4432	16816	8057	4583	6762	9445	12959	O
Q15166	Serum paraoxonase/lactonase 3	PON3	68.632	52	21	12	354	39.6	870	728	701	2842	802	518	903	3637	2867	2778	2600	2653	2034	1620	O
P04278	Sex hormone-binding globulin	SHBG	118.741	59	48	18	402	43.8	3827	3897	2079	3198	4097	2072	2796	11069	3907	12155	6949	5093	3296	8587	O
P10768	S-formylglutathione hydrolase	ESD	10.238	11	2	2	282	31.4	51	70	75	189	58	280	75	105	36	14	26	39	223	50	O
O75368	SH3 domain-binding glutamic acid-rich-like protein	SH3BGRL	15.971	35	3	3	114	12.8	477	585	702	961	791	955	848	334	166	452	349	143	493	367	O
Q9UJC5	SH3 domain-binding glutamic acid-rich-like protein 2	SH3BGRL2	8.063	23	2	2	107	12.3	70	77	123	99	115	142	208	16	7	100	65	11	29	32	O
Q9H299	SH3 domain-binding glutamic acid-rich-like protein 3	SH3BGRL3	16.829	48	7	5	93	10.4	104	53	80	452	135	291	160	28	13	103	70	16	47	53	O
Q9HAT2	Sialate O-acetylesterase	SIAE	4.564	3	2	2	523	58.3	574	397	515	621	792	391	587	373	388	282	431	325	361	206	O
Q08ET2	Sialic acid-binding Ig-like lectin 14	SIGLEC14	18.271	26	7	5	396	43.9	150	178	163	100	222	217	254	294	204	187	132	129	210	427	O
O94933	SLIT and NTRK-like protein 3	SLITRK3	10.516	3	2	2	977	108.9	112	101	43	55	6	57	108	193	44	68	54	110	36	57	O
Q8WVQ1	Soluble calcium-activated nucleotidase 1	CANT1	9.605	9	3	3	401	44.8	364	376	347	720	401	351	420	533	714	369	543	500	514	347	O
A1L4H1	Soluble scavenger receptor cysteine-rich domain-containing protein SSC5D	SSC5D	62.872	13	17	16	1573	165.6	891	983	877	771	1315	868	1233	645	1912	1116	1058	1268	856	987	O
Q99523	Sortilin	SORT1	9.836	3	2	2	831	92	61	41	34	45	92	25	49	41	59	36	31	30	26	24	O
P09486	SPARC	SPARC	81.063	59	39	15	303	34.6	3518	7825	7056	3865	9032	3690	6469	2815	4407	1992	5023	3393	3525	1951	O
Q14515	SPARC-like protein 1	SPARCL1	102.723	32	30	20	664	75.2	2108	3961	4200	1746	3227	1973	2862	3674	3262	3991	3774	3451	2317	3821	O
Q9HCB6	Spondin-1	SPON1	20.277	10	5	5	807	90.9	458	540	632	313	400	298	519	642	438	620	474	607	353	745	O
O75563	Src kinase-associated phosphoprotein 2	SKAP2	8.26	6	2	2	359	41.2	212	139	210	436	500	352	456			188	128	33	37	63	O
Q14247	Src substrate cortactin	CTTN	7.845	4	2	2	550	61.5	175	128	370	145	274	263	286	7	8	112	68	28	26	82	O
Q9NY15	Stabilin-1	STAB1	50.178	7	14	12	2570	275.3	576	544	507	560	611	586	761	679	566	672	582	593	633	592	O
P31948	Stress-induced-phosphoprotein 1	STIP1	18.415	12	5	5	543	62.6	216	368	440	920	333	695	365	233	172	245	214	130	634	317	O
P08254	Stromelysin-1	MMP3	20.877	16	8	8	477	53.9	755	1091	658	963	402	678	362	1568	621	828	1176	940	485	1987	O
O00391	Sulfhydryl oxidase 1	QSOX1	160.233	40	46	28	747	82.5	3051	2894	2425	2077	2965	3253	2876	4313	3050	3620	3258	3607	2775	4153	O
Q6ZRP7	Sulfhydryl oxidase 2	QSOX2	27.146	11	6	6	698	77.5	639	422	483	412	572	395	456	754	930	976	729	969	1020	755	O
P00441	Superoxide dismutase [Cu-Zn]	SOD1	15.977	17	3	2	154	15.9	342	887	910	315	613	1123	789	824	341	413	411	215	1261	601	O
P04179	Superoxide dismutase [Mn], mitochondrial	SOD2	48.46	50	13	9	222	24.7	665	1121	834	1030	951	1952	1234	2199	944	1094	1004	976	976	1574	O
Q6UWP8	Suprabasin	SBSN	39.721	31	8	8	590	60.5	870	1482	1233	443	798	450	666	807	1001	829	709	819	505	716	O
O60279	Sushi domain-containing protein 5	SUSD5	14.288	13	5	5	629	68															X
Q8TER0	Sushi, nidogen and EGF-like domain-containing protein 1	SNED1	26.874	6	5	5	1413	152.1	366	403	314	328	405	389	370	334	372	516	478	448	284	479	O
Q4LDE5	ushi, von Willebrand factor type A, EGF and pentraxin domain-containing protein	SVEP1	24.929	3	6	6	3571	389.9	503	326	463	232	440	202	351	332	140	335	246	221	213	311	O
Q99536	Synaptic vesicle membrane protein VAT-1 homolog	VAT1	5.767	10	2	2	393	41.9	105	190	148	95	134	132	161	113	148	106	140	155	156	138	O
P31431	Syndecan-4	SDC4	10.951	12	2	2	198	21.6	444	733	702	450	739	388	543	177	278	94	316	200	257	174	O
Q9Y490	Talin-1	TLN1	308.761	37	74	66	2541	269.6	6902	3003	8145	7071	6656	24187	7671	883	451	5962	3510	1238	2439	5138	O
Q7Z7G0	Target of Nesh-SH3	ABI3BP	49.148	14	12	10	1075	118.6	1040	1917	1384	1115	2027	1454	1385	1303	2151	1903	1690	2185	959	1807	O
Q8TB96	T-cell immunomodulatory protein	ITFG1	12.39	4	2	2	612	68.1	20	38	42	37	34	28	33	32	33	34	37	34	23	40	O
P17987	T-complex protein 1 subunit alpha	TCP1	5.177	6	2	2	556	60.3															X
P40227	T-complex protein 1 subunit zeta	CCT6A	4.648	4	2	2	531	58	39	30	77	113	44	90	36	39	16	28	30	27	91	46	O
P24821	Tenascin	TNC	170.229	23	45	38	2201	240.7	2648	5428	2856	4030	2100	3315	1853	6507	3273	2835	3493	3614	2723	3578	O
Q9UQP3	Tenascin-N	TNN	7.596	3	3	3	1299	143.9	337	302	253	281	505	285	454	434	532	306	364	295	226	348	O
P22105	Tenascin-X	TNXB	317.414	22	99	61	4244	458.1	6027	9428	6725	7314	8761	4968	6469	10806	7926	9367	7581	8643	5601	11531	O
P05452	Tetranectin	CLEC3B	131.425	74	76	14	202	22.5	6396	9707	9004	6940	8853	5348	7856	9221	9296	10299	8997	10743	5938	10652	O
P10599	Thioredoxin	TXN	13.545	38	6	4	105	11.7	949	1377	1345	2516	1654	1678	1782	769	523	631	510	452	1456	940	O
Q8NBS9	Thioredoxin domain-containing protein 5	TXNDC5	9.479	6	3	3	432	47.6	250	280	324	338	380	174	325	196	189	285	239	222	139	248	O
O43396	Thioredoxin-like protein 1	TXNL1	16.932	22	4	4	289	32.2	55	61	85	157	47	170	82	68	29	51	36	29	108	57	O
P07996	Thrombospondin-1	THBS1	350.614	57	195	59	1170	129.3	22639	23061	19750	30443	29640	22588	30722	11959	7563	4804	11513	7745	9094	4655	O
P35443	Thrombospondin-4	THBS4	158.034	43	38	23	961	105.8	1352	3372	1025	1501	2141	1663	1857	1377	2061	2021	1499	1436	1744	2037	O
P04216	Thy-1 membrane glycoprotein	THY1	10.574	18	2	2	161	17.9	66	150	91	78	106	70	79	101	104	100	108	128	73	139	O
Q9UKU6	Thyrotropin-releasing hormone-degrading ectoenzyme	TRHDE	34.432	8	9	7	1024	116.9	855	456	882	703	1131	2227	1183	237	376	263	232	421	1019	481	O
P05543	Thyroxine-binding globulin	SERPINA7	234.568	54	168	21	415	46.3	13690	16942	16601	14085	24280	15829	23013	30287	18897	23694	14913	20335	18025	17340	O
P04066	Tissue alpha-L-fucosidase	FUCA1	38.858	31	13	12	466	53.7	1030	1811	1473	1488	1365	3211	1313	2553	1049	1767	1978	2197	1454	2526	O
P10646	Tissue factor pathway inhibitor	TFPI	31.313	32	8	8	304	35	806	1122	908	393	813	777	597	520	729	714	697	645	435	689	O
Q8WZ42	Titin	TTN	8.915	0	4	4	34350	3813.7	100	450	105	80	1171	133	114	303	106	258	193	72	117	191	O
P37837	Transaldolase	TALDO1	39.761	34	16	12	337	37.5	1860	2121	2568	10410	2850	7549	2514	3836	1274	1771	1433	1336	2867	1755	O
P20061	Transcobalamin-1	TCN1	15.501	11	3	3	433	48.2	73	142	75	52	111	27	73	46	68	80	88	80	57	85	O
P20062	Transcobalamin-2	TCN2	17.614	15	7	6	427	47.5	920	954	677	814	870	840	801	943	621	762	811	932	596	1365	O
P02786	Transferrin receptor protein 1	TFRC	80.755	26	23	17	760	84.8	2160	2022	2262	1237	3032	1218	3027	1579	3785	3639	3053	3194	2446	2737	O
Q03167	Transforming growth factor beta receptor type 3	TGFBR3	30.671	10	6	6	851	93.4	889	689	574	753	857	516	683	1116	735	872	814	928	602	1285	O
P01137	Transforming growth factor beta-1 proprotein	TGFB1	54.539	33	13	12	390	44.3	1258	1389	1591	1551	2204	1181	1797	931	1383	432	1224	869	1189	475	O
Q15582	Transforming growth factor-beta-induced protein ig-h3	TGFBI	193.152	60	60	30	683	74.6	4722	4209	2666	3954	3416	5805	5461	8361	4649	5261	6337	5204	3712	8795	O
Q01995	Transgelin	TAGLN	9.765	12	2	2	201	22.6	317	378	326	383	444	454	246	574	363	406	380	413	308	514	O
P37802	Transgelin-2	TAGLN2	52.802	54	13	9	199	22.4	919	633	1290	2764	1812	2959	2160	126	19	963	528	107	226	426	O
O43493	Trans-Golgi network integral membrane protein 2	TGOLN2	9.302	7	3	3	437	45.9	302	449	384	250	497	232	295	349	378	358	326	358	189	502	O
P55072	Transitional endoplasmic reticulum ATPase	VCP	23.666	9	5	5	806	89.3	112	138	301	807	139	915	125	229	74	112	98	74	497	144	O
P29401	Transketolase	TKT	58.137	31	14	14	623	67.8	699	944	1241	4355	1206	3521	993	2197	844	951	683	766	1131	702	O

P13693	Translationally-controlled tumor protein	TPT1	7.152	12	2	2	172	19.6	492	442	857	1245	766	1487	916	350	51	612	401	152	421	333	O
Q14956	Transmembrane glycoprotein NMB	GPNMB	26.239	10	4	4	572	63.9	232	493	368	158	372	197	337	361	322	354	297	299	249	364	O
Q24JP5	Transmembrane protein 132A	TMEM132A	6.648	3	2	2	1023	110															X
Q8N3T6	Transmembrane protein 132C	TMEM132C	22.077	5	5	4	1108	121.7	200	150	145	173	151	162	115	233	182	221	176	229	124	395	O
Q6ICL3	Transport and Golgi organization protein 2 homolog	C22orf25; TANGO2	8.492	9	2	2	276	30.9	103	72	72	105	59	143	98	130	58	75	63	75	113	74	O
P02766	Transthyretin	TTR	97.96	65	40	11	147	15.9	2758	3550	3632	3979	3152	2150	3539	3609	8816	4610	7038	4784	4062	4431	O
Q86YW5	Trem-like transcript 1 protein	TREML1	16.101	13	7	3	311	32.7	1176	1646	2033	267	1890	864	1527	21	29	146	241	96	42	79	O
Q5T2D2	Trem-like transcript 2 protein	TREML2	7.607	7	2	2	321	35.1	41	79	67	38	87	41	44	30	39	41	38	37	24	32	O
P55084	Trifunctional enzyme subunit beta, mitochondrial	HADHB	9.138	6	3	3	474	51.3	236	199	367	250	338	156	270	218	255	252	243	275	259	193	O
P60174	Triosephosphate isomerase	TPI1	66.258	58	20	13	286	30.8	984	1176	1687	5843	1220	6907	1365	2733	543	990	771	578	2317	1044	O
O14773	Tripeptidyl-peptidase 1	TPP1	13.538	10	4	4	563	61.2	336	244	274	409	339	336	297	204	253	173	262	222	243	197	O
P09493	Tropomyosin alpha-1 chain	TPM1	60.283	48	24	8	284	32.7	1407	1195	2349	2729	2399	2882	2223	1040	1032	1424	1472	1344	1402	1741	O
P06753	Tropomyosin alpha-3 chain	TPM3	41.823	32	19	3	285	32.9	402	315	518	602	619	583	568	176	245	393	347	293	336	527	O
P67936	Tropomyosin alpha-4 chain	TPM4	66.669	46	24	8	248	28.5	1811	1193	2314	2679	3112	6139	3787	198	82	2313	1353	416	722	2020	O
P07951	Tropomyosin beta chain	TPM2	39.445	27	14	2	284	32.8	207	183	444	332	351	1171	377	37	45	295	176	82	106	316	O
P19429	Troponin I, cardiac muscle	TNNI3	6.557	10	2	2	210	24															X
P07477	Trypsin-1	PRSS1	16.739	25	6	3	247	26.5	739	704	519	531	609	571	722	1047	596	661	579	764	770	642	O
P07478	Trypsin-2	PRSS2	12.858	16	5	2	247	26.5	27	43	25	19	32	23	27	23	15	12	30	18	31	29	O
P23381	Tryptophan--tRNA ligase, cytoplasmic	WARS	27.805	21	7	7	471	53.1	553	631	537	449	529	867	455	458	316	316	256	383	398	443	O
Q8WUA8	Tsukushin	TSKU	18.778	15	5	4	353	37.8	726	557	408	1014	520	311	535	397	567	521	403	383	337	527	O
Q71U36	Tubulin alpha-1A chain	TUBA1A	54.006	35	11	5	451	50.1	739	583	1805	1208	1128	4058	1428	236	84	587	364	296	631	981	O
P68366	Tubulin alpha-4A chain	TUBA4A	51.627	33	10	4	448	49.9	519	352	1098	725	645	2857	527	142	64	369	260	222	338	615	O
P07437	Tubulin beta chain	TUBB	34.039	26	10	4	444	49.6	412	360	1026	873	585	2160	659	253	129	410	345	255	534	558	O
Q9H4B7	Tubulin beta-1 chain	TUBB1	41.432	26	10	7	451	50.3	316	250	312	310	409	2562	390	109	105	239	156	192	203	600	O
P04350	Tubulin beta-4A chain	TUBB4A	23.788	20	6	2	444	49.6	54	40	133	109	63	194	67	39	30	50	41	31	92	55	O
O75347	Tubulin-specific chaperone A	TBCA	6.407	18	2	2	108	12.8	15	41	52	88	27	73	29	27	5	19	16	12	44	22	O
Q9Y275	Tumor necrosis factor ligand superfamily member 13B	TNFSF13B	10.407	10	2	2	285	31.2	49	41	37	63	59	34	41	73	54	50	42	49	46	66	O
P98066	Tumor necrosis factor-inducible gene 6 protein	TNFAIP6	13.955	14	3	3	277	31.2	134	139	190	185	224	164	187	240	142	167	191	173	176	249	O
Q9Y274	Type 2 lactosamine alpha-2,3-sialyltransferase	ST3GAL6	14.748	14	4	4	331	38.2	427	689	670	521	818	510	734	453	575	406	542	510	512	543	O
Q12866	Tyrosine-protein kinase Mer	MERTK	8.861	4	3	3	999	110.2	129	234	243	148	227	159	142	163	168	235	259	241	119	266	O
P35590	Tyrosine-protein kinase receptor Tie-1	TIE1	55.083	15	12	11	1138	125	639	694	675	754	729	810	657	1067	1056	1021	1042	937	766	1150	O
P30530	Tyrosine-protein kinase receptor UFO	AXL	20.6	5	4	3	894	98.3	246	484	341	202	484	193	282	444	439	420	326	491	178	560	O
P29350	Tyrosine-protein phosphatase non-receptor type 6	PTPN6	15.681	10	4	4	595	67.5	333	185	359	1096	372	1062	414	110	98	275	184	81	229	188	O
P78324	Tyrosine-protein phosphatase non-receptor type substrate 1	SIRPA	22.922	12	6	5	504	54.9	438	948	562	425	935	428	572	550	569	695	645	566	396	932	O
P54578	Ubiquitin carboxyl-terminal hydrolase 14	USP14	10.609	6	3	3	494	56	89	158	170	522	94	438	155	253	53	85	71	57	418	119	O
Q9Y4E8	Ubiquitin carboxyl-terminal hydrolase 15	USP15	9.757	3	2	2	981	112.3	11	20	28	80	20	55	28	33	6	9	9	6	58	16	O
P45974	Ubiquitin carboxyl-terminal hydrolase 5	USP5	4.716	4	2	2	858	95.7	7	17	23	43	11	41	17	20	0	7	5		43	12	O
P15374	Ubiquitin carboxyl-terminal hydrolase isozyme L3	UCHL3	6.049	11	2	2	230	26.2															X
Q96FW1	Ubiquitin thioesterase OTUB1	OTUB1	11.754	18	3	3	271	31.3	114	125	172	220	124	258	151	158	111	123	126	105	155	118	O
P62987	Ubiquitin-60S ribosomal protein L40	UBA52	11.449	27	5	3	128	14.7	463	767	819	2124	594	672	721	439	335	456	340	251	931	477	O
Q9BSL1	Ubiquitin-associated domain-containing protein 1	UBAC1	7.034	6	2	2	405	45.3	58	110	98	283	66	119	101	106	65	59	49	40	275	109	O
P68036	Ubiquitin-conjugating enzyme E2 L3	UBE2L3	9.13	24	2	2	154	17.9	154	168	230	326	252	328	262	150	61	134	92	75	222	130	O
P61088	Ubiquitin-conjugating enzyme E2 N	UBE2N	5.907	16	2	2	152	17.1	173	321	393	911	251	779	365	295	126	220	161	73	494	221	O
Q13404	Ubiquitin-conjugating enzyme E2 variant 1	UBE2V1; TMEM189-UBE2V1	9.069	18	5	4	147	16.5	443	606	694	1346	546	1432	700	636	116	418	301	182	1111	449	O
P61960	Ubiquitin-fold modifier 1	UFM1	8.311	51	2	2	85	9.1	193	164	212	371	273	306	302	126	91	204	155	86	180	175	O
P22314	Ubiquitin-like modifier-activating enzyme 1	UBA1	19.754	8	6	6	1058	117.8	184	198	382	861	206	930	271	429	129	203	162	123	513	246	O
Q7Z7M8	UDP-GlcNAc:betaGal beta-1,3-N-acetylglucosaminyltransferase 8	B3GNT8	45.046	39	10	9	397	43.4	357	467	547	636	451	724	656	1376	516	704	565	570	641	521	O
P07911	Uromodulin	UMOD	44.34	18	15	11	640	69.7	330	517	525	283	434	268	347	615	457	579	677	613	356	636	O
P06132	Uroporphyrinogen decarboxylase	UROD	11.329	11	3	3	367	40.8	59	99	129	443	80	549	111	289	16	66	58	68	187	88	O
P11684	Uteroglobin	SCGB1A1	4.686	12	2	2	91	10	526	178	305	73	329	156	180	658	217	304	298	330	308	316	O
Q16851	UTP--glucose-1-phosphate uridylyltransferase	UGP2	23.146	14	6	6	508	56.9	442	288	731	1382	714	1496	219	472	320	370	180	222	424	507	O
P54725	UV excision repair protein RAD23 homolog A	RAD23A	15.728	15	3	2	363	39.6	30	96	101	130	60	126	67	71	38	40	28	28	194	53	O
P54727	UV excision repair protein RAD23 homolog B	RAD23B	8.501	9	4	2	409	43.1	304	326	445	456	531	399	571	107	82	287	200	69	183	169	O
P19320	Vascular cell adhesion protein 1	VCAM1	199.327	60	59	32	739	81.2	4027	4429	4439	2830	6100	2570	4427	5635	5005	6989	5674	4423	2626	7278	O
P49767	Vascular endothelial growth factor C	VEGFC	11.073	11	4	4	419	46.9	143	201	249	114	232	116	226	92	136	66	145	96	133	72	O
P35968	Vascular endothelial growth factor receptor 2	KDR	8.334	3	3	3	1356	151.4	206	405	338	272	341	243	267	271	289	236	277	254	235	309	O
P35916	Vascular endothelial growth factor receptor 3	FLT4	97.121	16	22	18	1363	152.7	1270	2462	2227	1468	2298	1604	1304	1661	1813	1546	1948	1611	1357	1996	O
P50552	Vasodilator-stimulated phosphoprotein	VASP	31.242	16	7	7	380	39.8	493	286	533	945	807	765	1112	26	14	387	259	63	100	207	O
Q6EMK4	Vasorin	VASN	51.67	16	15	9	673	71.7	743	943	661	756	1057	809	776	1104	1141	913	656	974	672	976	O
P13611	Versican core protein	VCAN	26.767	3	9	8	3396	372.6	737	971	959	877	980	1257	1094	1206	838	1142	1565	1564	671	1115	O
Q12907	Vesicular integral-membrane protein VIP36	LMAN2	28.098	24	9	7	356	40.2	712	1226	1063	984	1208	700	901	1400	1009	1100	943	901	851	1232	O
P08670	Vimentin	VIM	32.918	22	12	8	466	53.6	424	386	777	3751	711	2031	760	399	234	492	449	246	761	326	O
P18206	Vinculin	VCL	198.078	44	51	40	1134	123.7	3482	3802	4580	6163	4055	16224	3476	4828	1641	3989	3071	2844	2704	4513	O
P02774	Vitamin D-binding protein	GC	606.337	81	1135	47	474	52.9	66630	121351	111771	80456	95832	61697	117314	94289	100362	102330	119617	104449	90294	113340	O
P04070	Vitamin K-dependent protein C	PROC	97.623	44	37	17	461	52	5679	5972	6611	4754	5152	4470	6344	4288	5897	4407	5539	6211	4894	5983	O
P07225	Vitamin K-dependent protein S	PROS1	224.163	51	121	30	676	75.1	10379	13287	10913	7821	8741	10786	13273	8617	11821	9161	11626	11053	8939	9126	O
P22891	Vitamin K-dependent protein Z	PROZ	107.017	47	55	14	400	44.7	2715	9166	2632	6375	2310	5281	2297	6392	6778	6990	6406	7767	7111	11725	O
P04004	Vitronectin	VTN	228.957	63	336	22	478	54.3	24959	27267	34972	25165	33114	24405	30303	14734	22781	19755	23010	23698	28801	32423	O
P54289	Voltage-dependent calcium channel subunit alpha-2/delta-1	CACNA2D1	170.89	34	39	29	1103	124.5	2570	3132	3466	2937	2380	2640	4000	4580	3707	4119	4047	3901	2295	4628	O
P04275	von Willebrand factor	VWF	664.688	53	196	114	2813	309.1	19471	8240	5856	12138	8638	4750	7504	24581	22242	25279	23223	22024	18437	22592	O
Q9Y279	V-set and immunoglobulin domain-containing protein 4	VSIG4	8.302	7	2	2	399	44	232	377	405	151	362	164	195	312	275	330	278	298	74	395	O
Q15904	V-type proton ATPase subunit S1	ATP6AP1	13.254	7	2	2	470	52	144	175	155	101	184	96	152	114	140	152	163	152	131	193	O
Q8TEU8	WAP, Kazal, immunoglobulin, Kunitz and NTR domain-containing protein 2	WFIKKN2	3.835	3	2	2	576	63.9	189	280	279	110	215	146	246	227	345	249	262	285	184	348	O
O75083	WD repeat-containing protein 1	WDR1	56.652	27	13	12	606	66.2	304	204	362	1338	339	1945	320	377	19	248	208	152	291	320	O
O43895	Xaa-Pro aminopeptidase 2	XPNPEP2	29.456	15	8	8	674	75.6	158	651	573	667	383	135	587	1161	140	292	623	706	397	138	O
P12955	Xaa-Pro dipeptidase	PEPD	81	28	24	12	493	54.5	976	915	720	1362	945	1363	975	2865	1276	1194	1052	1292	1218	1269	O
Q9H1B5	Xylosyltransferase 2	XYLT2	24.503	9	7	6	865	96.7	351	380	428	580	417	414	458	330	320	194	284	227	284	151	O
P25311	Zinc-alpha-2-glycoprotein	AZGP1	265.807	68	313	26	298	34.2	19202	26802	22188	15622	21632	13131	20446	24058	23695	19857	22696	23069	16676	28728	O
Q15942	Zyxin	ZYX	11.136	10	5	4	572	61.2	434	338	1326	227	535	523	522	26	55	107	121	42	75	102	O

Supplementary Table S3. Proteins showing statistically significant changes (*P* -value <0.05) from TMT-labeling based quantitative analyses of psoriasis groups with and without CVD risk factors and a healthy group using one-way analysis of variance (ANOVA)

[a] CVD risk O represents normalized signal-to-noise ratio values from psoriasis group with CVD risk factors.
[b] CVD risk X represents normalized signal-to-noise ratio values from psoriasis group without CVD risk factors.
[c] Control represents normalized signal-to-noise ratio values from a healthy control group.
[d] ANOVA *P*-values are obtained from ANOVA analysis of three kinds of samples composed of psoriasis groups with and without CVD risk factors and a healthy control group.
[e] Scheffe *P*-values were obtained from post-hoc analysis performed by pairwise multiple comparison procedures (Scheffe's method) between a psoriasis group with CVD risk factors and a healthy control group.
[f] Scheffe *P*-values were obtained from post-hoc analysis performed by pairwise multiple comparison procedures (Scheffe's method) between a psoriasis group without CVD risk factors and a healthy control group.
[g] Scheffe *P*-values were obtained from post-hoc analysis performed by pairwise multiple comparison procedures (Scheffe's method) between psoriasis groups with and without CVD risk factors.
[h] Ratio represents relative normalized signal-to-noise value ratios between a psoriasis group with CVD risk factors and a healthy control group.
[i] Ratio represents relative normalized signal-to-noise value ratios between a psoriasis group without CVD risk factors and a healthy control group.
[j] Ratio represents relative normalized signal-to-noise value ratios between psoriasis groups with and without CVD risk factors.

Accession No.	Protein Description	Gene Symbol	CVD risk O 1[a]	CVD risk O 2[a]	CVD risk O 3[a]	CVD risk O 4[a]	CVD risk O 5[a]	CVD risk O 6[a]	CVD risk O 7[a]	CVD risk X 1[b]	CVD risk X 2[b]	CVD risk X 3[b]	CVD risk X 4[b]	Control 1[c]	Control 2[c]	Control 3[c]	ANOVA *P*-value[d]	Scheffe *P*-value[e]	Scheffe *P*-value[f]	Scheffe *P*-value[g]	Ratio (CVD risk O/Control)[h]	Ratio (CVD risk X/Control)[i]	Ratio (CVD risk O/CVD risk X)[j]
Q92854	Semaphorin-4D	SEMA4D	442	648	590	769	773	612	646	284	198	189	276	228	226	211	1.652E-06	1.560E-05	9.406E-01	9.741E-06	2.882	1.067	2.702
O00754	Lysosomal alpha-mannosidase	MAN2B1	255	251	262	265	333	277	286	196	196	178	186	149	164	186	6.723E-06	2.078E-05	2.056E-01	1.333E-04	1.656	1.136	1.458
P08514	Integrin alpha-IIb	ITGA2B	102	80	118	152	137	163	158	46	53	52	48	27	42	46	1.157E-05	4.044E-05	3.298E-01	1.734E-04	3.377	1.287	2.624
P07996	Thrombospondin-1	THBS1	22639	23061	19750	30443	29640	22588	30722	11959	7563	4804	11513	7745	9094	4655	5.047E-05	2.305E-04	6.939E-01	4.174E-04	3.566	1.251	2.851
O60234	Glia maturation factor gamma	GMFG	196	176	260	371	315	395	322	81	71	104	82	76	143	119	5.451E-05	1.798E-03	4.968E-01	1.077E-04	2.585	0.751	3.442
Q9HCN6	Platelet glycoprotein VI	GP6	1720	2019	3473	1022	2082	1292	1865	62	136	399	416	218	198	236	6.164E-05	7.323E-04	9.627E-01	2.081E-04	8.843	1.162	7.608
P21926	CD9 antigen	CD9	501	397	827	251	523	873	614	179	108	185	170	114	163	142	1.205E-04	6.642E-04	8.903E-01	6.960E-04	4.085	1.152	3.548
Q6ZRP7	Sulfhydryl oxidase 2	QSOX2	639	422	483	412	572	395	456	754	930	976	729	969	1020	755	1.248E-04	6.248E-04	8.402E-01	7.925E-04	0.528	0.926	0.570
Q5SQ64	Lymphocyte antigen 6 complex locus protein G6f	LY6G6F	137	129	398	78	167	74	207	7	6	36	32	12	16	8	1.408E-04	7.293E-04	8.690E-01	8.427E-04	14.204	1.703	8.342
P16278	Beta-galactosidase	GLB1	223	147	205	195	251	137	236	50	48	94	109	79	74	58	1.521E-04	1.218E-03	9.994E-01	6.025E-04	2.833	1.070	2.647
P08648	Integrin alpha-5	ITGA5	125	158	141	127	199	125	159	316	242	285	223	236	215	255	1.545E-04	3.666E-03	6.302E-01	3.134E-04	0.628	1.132	0.554
**Q15942**	**Zyxin**	**ZYX**	**434**	**338**	**1326**	**227**	**535**	**523**	**522**	**26**	**55**	**107**	**121**	**42**	**75**	**102**	**1.767E-04**	**1.584E-03**	**9.951E-01**	**6.255E-04**	**7.653**	**1.057**	**7.239**
Q86YW5	Trem-like transcript 1 protein	TREML1	1176	1646	2033	267	1890	864	1527	21	29	146	241	96	42	79	2.146E-04	1.732E-03	9.999E-01	7.968E-04	18.538	1.504	12.328
P16109	P-selectin	SELP	2291	3155	6191	1861	3034	1581	1921	566	776	787	1097	764	660	643	2.223E-04	1.143E-03	8.975E-01	1.228E-03	4.152	1.170	3.549
P35590	Tyrosine-protein kinase receptor Tie-1	TIE1	639	694	675	754	729	810	657	1067	1056	1021	1042	937	766	1150	2.540E-04	9.162E-03	4.361E-01	4.086E-04	0.745	1.100	0.677
P29122	Proprotein convertase subtilisin/kexin type 6	PCSK6	507	635	579	725	635	667	772	382	384	258	440	320	403	238	2.549E-04	8.849E-04	6.636E-01	2.157E-03	2.016	1.142	1.765
Q8NI99	Angiopoietin-related protein 6	ANGPTL6	287	243	261	389	245	453	302	790	749	543	491	615	875	570	3.240E-04	1.712E-03	9.326E-01	1.614E-03	0.453	0.937	0.484
P01008	Antithrombin-III	SERPINC1	34544	41108	38709	38802	29599	32812	37930	75418	53704	59434	51287	66567	46280	72408	3.683E-04	2.272E-03	9.827E-01	1.548E-03	0.586	0.971	0.604
**Q14847**	**LIM and SH3 domain protein 1**	**LASP1**	**723**	**579**	**1425**	**1271**	**1306**	**1424**	**1174**	**176**	**105**	**447**	**332**	**163**	**344**	**344**	**3.706E-04**	**4.386E-03**	**9.054E-01**	**9.592E-04**	**3.979**	**0.933**	**4.264**
P02671	Fibrinogen alpha chain	FGA	7183	10450	8477	3542	8426	5808	9146	2317	1849	3170	3245	2284	3889	2148	5.401E-04	4.384E-03	9.898E-01	1.683E-03	2.731	0.954	2.864
Q9UJU6	Drebrin-like protein	DBNL	105	77	144	244	204	194	221	22	16	68	54	12	43	33	6.089E-04	2.350E-03	8.272E-01	3.671E-03	5.771	1.359	4.247
P30044	Peroxiredoxin-5, mitochondrial	PRDX5	506	367	753	807	766	997	945	135	204	355	283	213	322	264	6.089E-04	7.221E-03	8.821E-01	1.445E-03	2.756	0.916	3.009
P46109	Crk-like protein	CRKL	512	442	691	636	992	809	1083	83	54	301	215	99	210	256	6.377E-04	9.723E-03	7.749E-01	1.306E-03	3.919	0.867	4.521
P40197	Platelet glycoprotein V	GP5	4268	4296	2504	6814	5911	5624	5639	2276	2110	929	2386	1786	2019	1453	8.425E-04	1.198E-02	8.916E-01	2.550E-03	2.857	1.098	2.602
P31431	Syndecan-4	SDC4	444	733	702	450	739	388	543	177	278	94	316	200	257	174	9.259E-04	7.497E-03	9.773E-01	2.594E-03	2.718	1.029	2.641
P05106	Integrin beta-3	ITGB3	235	228	284	347	396	413	362	101	25	111	141	67	92	130	9.583E-04	9.957E-03	9.075E-01	2.263E-03	3.365	0.981	3.430
**Q05682**	**Caldesmon**	**CALD1**	**341**	**205**	**717**	**181**	**414**	**230**	**407**	**39**	**23**	**125**	**93**	**39**	**54**	**124**	**1.012E-03**	**8.604E-03**	**9.640E-01**	**2.690E-03**	**4.911**	**0.964**	**5.096**
P08581	Hepatocyte growth factor receptor	MET	911	1178	876	999	1078	1059	1163	1717	1383	1367	1614	1612	1196	1840	1.122E-03	6.528E-03	9.974E-01	3.893E-03	0.670	0.981	0.683
P04275	von Willebrand factor	VWF	19471	8240	5856	12138	8638	4750	7504	24581	22242	25279	23223	22024	18437	22592	1.130E-03	1.198E-02	8.916E-01	2.550E-03	0.453	1.134	0.399
Q9Y251	Heparanase	HPSE	1684	1226	1927	3014	2850	1130	1899	834	939	374	876	616	719	316	1.168E-03	2.922E-03	6.065E-01	1.041E-02	3.565	1.373	2.596
P54727	UV excision repair protein RAD23 homolog B	RAD23B	304	326	445	456	531	399	571	107	82	287	200	69	183	169	1.500E-03	5.611E-03	8.913E-01	7.377E-03	3.090	1.205	2.565
Q06481	Amyloid-like protein 2	APLP2	259	445	308	182	423	183	281	92	143	100	175	124	139	111	1.561E-03	9.489E-03	9.997E-01	4.829E-03	2.391	1.025	2.333
Q9NRW1	Ras-related protein Rab-6B	RAB6B	607	405	570	652	619	553	984	159	195	424	306	208	254	364	1.657E-03	1.282E-02	9.676E-01	4.226E-03	2.275	0.983	2.316
Q9H1B5	Xylosyltransferase 2	XYLT2	351	380	428	580	417	414	458	330	320	194	284	227	284	151	1.785E-03	2.970E-03	3.511E-01	2.879E-02	1.958	1.276	1.534
P06702	Protein S100-A9	S100A9	3007	3636	2969	11551	5168	4616	4875	1737	1901	1179	1272	1342	2877	1057	1.960E-03	1.401E-02	9.780E-01	5.082E-03	2.910	0.866	3.362
P10619	Lysosomal protective protein	CTSA	1632	906	1144	1799	1836	852	1794	820	805	400	696	536	646	534	1.991E-03	6.464E-03	8.451E-01	1.060E-02	2.488	1.188	2.093
P23528	Cofilin-1	CFL1	2066	1348	2489	3605	2675	5386	3450	215	320	1193	850	450	1374	1058	2.048E-03	4.724E-02	5.262E-01	2.882E-03	3.126	0.671	4.660
Q15166	Serum paraoxonase/lactonase 3	PON3	870	728	701	2842	802	518	903	3637	2867	2778	2600	2653	2034	1620	2.183E-03	4.654E-02	5.540E-01	3.126E-03	0.500	1.413	0.354
P58215	Lysyl oxidase homolog 3	LOXL3	221	270	388	209	357	249	370	147	116	76	182	162	200	111	2.207E-03	3.573E-02	6.712E-01	3.526E-03	1.874	0.828	2.263
Q15555	Microtubule-associated protein RP/EB family member 2	MAPRE2	195	95	166	228	333	458	436	5		88	66	23	24	60	2.404E-03	2.106E-02	9.154E-01	5.198E-03	7.659	1.480	5.175
P10586	Receptor-type tyrosine-protein phosphatase F	PTPRF	1792	1534	1422	1671	1520	1701	1729	2656	2125	2062	1882	1876	1890	2138	2.446E-03	5.944E-02	4.939E-01	3.281E-03	0.825	1.108	0.745
Q9UKU6	Thyrotropin-releasing hormone-degrading ectoenzyme	TRHDE	855	456	882	703	1131	2227	1183	237	376	263	232	421	1019	481	2.573E-03	3.133E-01	1.060E-01	2.576E-03	1.659	0.433	3.835
P21291	Cysteine and glycine-rich protein 1	CSRP1	208	85	227	189	232	146	320	10	14	92	67	14	35	63	2.575E-03	1.429E-02	9.998E-01	7.556E-03	5.418	1.238	4.378
P04424	Argininosuccinate lyase	ASL	737	453	524	837	817	1755	729	228	267	303	356	324	395	538	2.608E-03	7.522E-02	4.263E-01	3.284E-03	1.995	0.688	2.898
P51149	Ras-related protein Rab-7a	RAB7A	199	117	221	563	252	345	354	42	32	147	95	52	72	105	2.619E-03	1.897E-02	9.645E-01	6.280E-03	3.841	1.033	3.719
P19021	Peptidyl-glycine alpha-amidating monooxygenase	PAM	1360	1342	1149	1137	1235	1170	1385	1764	1699	1659	1524	1527	1221	1727	3.255E-03	1.074E-01	3.675E-01	3.830E-03	0.841	1.114	0.755
P10599	Thioredoxin	TXN	949	1377	1345	2516	1654	1678	1782	769	523	631	510	452	1456	940	3.295E-03	8.600E-02	4.473E-01	4.125E-03	1.700	0.640	2.655
Q15389	Angiopoietin-1	ANGPT1	1246	1257	1537	1148	1681	1128	1657	612	863	284	866	602	864	338	3.883E-03	1.513E-02	9.691E-01	1.387E-02	2.294	1.091	2.102
O43852	Calumenin	CALU	1293	1870	2049	1343	2101	1006	1752	666	1024	481	1136	820	909	593	3.922E-03	1.760E-02	9.943E-01	1.226E-02	2.107	1.068	1.973
Q14247	Src substrate cortactin	CTTN	175	128	370	145	274	263	286	7	8	112	68	28	26	82	3.995E-03	4.280E-02	8.040E-01	6.885E-03	5.169	1.075	4.806
Q02763	Angiopoietin-1 receptor	TEK	105	167	122	150	152	132	150	194	202	190	168	217	163	231	4.297E-03	1.178E-02	8.316E-01	2.183E-02	0.685	0.925	0.741
Q99439	Calponin-2	CNN2	628	234	438	1031	858	418	904	17	17	290	205	61	70	138	4.635E-03	3.390E-02	9.333E-01	9.596E-03	7.208	1.479	4.873
P62491	Ras-related protein Rab-11A	RAB11A	203	97	209	119	264	201	332	20	26	114	78	49	51	100	4.655E-03	5.034E-02	7.874E-01	7.720E-03	3.067	0.894	3.429
Q13510	Acid ceramidase	ASAH1	349	278	274	484	476	206	423	206	235	180	252	203	163	137	4.825E-03	7.602E-03	4.396E-01	5.707E-02	2.121	1.302	1.630
O75563	Src kinase-associated phosphoprotein 2	SKAP2	212	139	210	436	500	352	456			188	128	33	37	63	5.289E-03	2.431E-02	9.997E-01	1.482E-02	7.419	3.554	2.087
O95866	Megakaryocyte and platelet inhibitory receptor G6b	MPIG6B	1260	1786	6780	653	1626	653	1572	7	40	594	438	76	95	101	5.372E-03	2.500E-02	9.999E-01	1.483E-02	22.570	2.974	7.588
Q76LX8	A disintegrin and metalloproteinase with thrombospondin motifs 13	ADAMTS13	2218	2149	1765	1728	2772	2317	2879	2906	3842	3571	3596	3937	2575	3131	5.399E-03	5.227E-02	8.208E-01	9.155E-03	0.703	1.082	0.650
Q13201	Multimerin-1	MMRN1	4563	6677	7081	4640	7953	5321	7047	3070	3920	1228	3515	2690	4046	1842	5.535E-03	2.707E-02	9.991E-01	1.455E-02	2.163	1.026	2.108
O75368	SH3 domain-binding glutamic acid-rich-like protein	SH3BGRL	477	585	702	961	791	955	848	334	166	452	349	143	493	367	5.566E-03	2.333E-02	9.946E-01	1.663E-02	2.271	0.973	2.335
O43505	Beta-1,4-glucuronyltransferase 1	B3GNT1; B4GAT1	488	499	527	458	422	545	608	796	716	595	687	791	534	761	5.592E-03	2.999E-02	9.915E-01	1.364E-02	0.729	1.005	0.725
P23381	Tryptophan--tRNA ligase, cytoplasmic	WARS	553	631	537	449	529	867	455	458	316	316	256	383	398	443	5.667E-03	1.328E-01	4.457E-01	6.718E-03	1.409	0.825	1.707
P14543	Nidogen-1	NID1	3060	2213	2032	1585	2856	2139	2431	1658	1307	971	1436	1444	2080	1540	5.988E-03	1.569E-01	4.008E-01	6.832E-03	1.381	0.795	1.736
Q3ZCW2	Galectin-related protein	LGALSL	259	116	192	285	296	431	505	5		169	92	23	25	71	6.249E-03	2.636E-02	9.972E-01	1.791E-02	7.552	2.243	3.366
P33241	Lymphocyte-specific protein 1	LSP1	82	62	95	386	136	174	107	39	18	52	46	28	73	25	6.384E-03	3.109E-02	9.978E-01	1.614E-02	3.543	0.924	3.835
Q8IWL2	Pulmonary surfactant-associated protein A1	SFTPA1	245	181	138	222	135	111	182	330	284	276	278	284	229	500	6.522E-03	1.915E-02	9.084E-01	2.637E-02	0.513	0.864	0.594
O14773	Tripeptidyl-peptidase 1	TPP1	336	244	274	409	339	336	297	204	253	173	262	222	243	197	6.531E-03	2.946E-02	1.000E+00	1.744E-02	1.447	1.010	1.433
P06681	Complement C2	C2	23569	23363	22524	22658	21606	23068	27766	32314	27809	26832	25552	26991	28269	27694	6.628E-03	3.779E-02	9.765E-01	1.475E-02	0.850	1.017	0.836
P58546	Myotrophin	MTPN	134	144	232	262	268	351	262	60	12	123	66	35	108	95	6.737E-03	7.406E-02	7.471E-01	1.021E-02	2.972	0.817	3.636
Q9NZN3	EH domain-containing protein 3	EHD3	205	131	180	269	264	106	404	13	16	160	110	17	18	67	7.073E-03	1.592E-02	7.707E-01	3.863E-02	6.585	2.206	2.985
P30740	Leukocyte elastase inhibitor	SERPINB1	660	542	646	2146	1066	1514	1073	594	304	304	303	335	623	450	7.309E-03	7.331E-02	7.789E-01	1.135E-02	2.328	0.802	2.901
Q9HAT2	Sialate O-acetylesterase	SIAE	574	397	515	621	792	391	587	373	388	282	431	325	361	206	7.610E-03	1.171E-02	4.861E-01	7.636E-02	1.862	1.240	1.502
Q6YHK3	CD109 antigen	CD109	2818	4640	3433	3263	3520	4025	3855	6284	5226	5227	5042	4770	3574	6050	8.091E-03	1.320E-01	5.565E-01	1.008E-02	0.761	1.135	0.670
P63241	Eukaryotic translation initiation factor 5A-1	EIF5A	151	258	330	391	268	360	376	110	52	129	105	49	286	137	8.419E-03	8.536E-02	7.603E-01	1.259E-02	1.937	0.629	3.079
P50552	Vasodilator-stimulated phosphoprotein	VASP	493	286	533	945	807	765	1112	26	14	387	259	63	100	207	8.690E-03	6.083E-02	9.045E-01	1.569E-02	5.738	1.395	4.113
Q08380	Galectin-3-binding protein	LGALS3BP	2291	2631	2042	1716	2258	1887	2121	3271	7563	3492	2769	2014	1971	2677	8.714E-03	9.793E-01	4.608E-02	1.124E-02	0.962	1.925	0.500
P29279	CCN family member 2	CTGF	587	948	1031	621	792	527	826	410	631	205	460	347	405	386	8.762E-03	3.099E-02	9.843E-01	2.639E-02	2.007	1.124	1.786
P14618	Pyruvate kinase PKM	PKM	1811	2080	3089	4072	2719	5962	2070	1356	874	1696	1432	1209	1535	1790	9.057E-03	6.650E-02	8.843E-01	1.573E-02	2.061	0.886	2.326
Q14019	Coactosin-like protein	COTL1	532	266	558	1129	726	791	597	209	57	456	295	99	165	229	9.203E-03	2.265E-02	8.570E-01	4.029E-02	3.999	1.548	2.583
O14960	Leukocyte cell-derived chemotaxin-2	LECT2	515	711	783	662	928	639	642	343	523	466	530	542	539	525	9.272E-03	1.572E-01	5.260E-01	1.113E-02	1.302	0.870	1.497
P80303	Nucleobindin-2	NUCB2	82	106	104	78	109	78	99	79	72	43	68	61	70	44	9.474E-03	2.135E-02	8.095E-01	4.591E-02	1.604	1.120	1.432
O15117	FYN-binding protein 1	FYB; FYB1	145	111	129	135	219	165	216	2	7	78	64	18	25	50	9.501E-03	9.799E-02	7.404E-01	1.371E-02	5.173	1.220	4.239
P04278	Sex hormone-binding globulin	SHBG	3827	3897	2079	3198	4097	2072	2796	11069	3907	12155	6949	5093	3296	8587	9.581E-03	1.879E-01	4.639E-01	1.096E-02	0.555	1.506	0.368
Q08830	Fibrinogen-like protein 1	FGL1	95	69	61	40	33	27	87	138	118	178	133	64	168	108	9.682E-03	1.140E-01	6.797E-01	1.314E-02	0.520	1.253	0.415
O15335	Chondroadherin	CHAD	361	450	228	497	494	387	377	591	545	532	619	571	589	679	9.733E-03	2.635E-02	9.095E-01	3.723E-02	0.651	0.933	0.698
Q71U36	Tubulin alpha-1A chain	TUBA1A	739	583	1805	1208	1128	4058	1428	236	84	587	364	296	631	981	9.809E-03	2.631E-01	3.483E-01	1.041E-02	2.459	0.500	4.920
P36871	Phosphoglucomutase-1	PGM1	89	73	97	139	152	248	85	78	46	45	40	45	75	75	9.858E-03	1.056E-01	7.196E-01	1.386E-02	1.940	0.799	2.429
Q9UJC5	SH3 domain-binding glutamic acid-rich-like protein 2	SH3BGRL2	70	77	123	99	115	142	208	16	7	100	65	11	29	32	1.021E-02	2.522E-02	8.706E-01	4.271E-02	4.984	1.963	2.539
Q9ULZ3	Apoptosis-associated speck-like protein containing a CARD	PYCARD	62	118	177	596	165	272	119	92	32	48	48	38	76	48	1.057E-02	4.447E-02	9.995E-01	2.569E-02	3.989	1.019	3.914
Q9BYE9	Cadherin-related family member 2	CDHR2	2298	1207	1190	1000	5569	4944	2580	330	682	778	820	869	807	1105	1.088E-02	1.302E-01	6.594E-01	1.429E-02	2.895	0.704	4.113
P23284	Peptidyl-prolyl cis-trans isomerase B	PPIB	850	920	1035	1424	1117	847	1007	672	425	680	954	723	636	505	1.097E-02	3.089E-02	9.357E-01	3.836E-02	1.655	1.099	1.506
P68036	Ubiquitin-conjugating enzyme E2 L3	UBE2L3	154	168	230	326	252	328	262	150	61	134	92	75	222	130	1.181E-02	1.160E-01	7.382E-01	1.655E-02	1.727	0.765	2.256
P37802	Transgelin-2	TAGLN2	919	633	1290	2764	1812	2959	2160	126	19	963	528	107	226	426	1.183E-02	5.735E-02	9.823E-01	2.489E-02	7.081	1.617	4.379

P52907	F-actin-capping protein subunit alpha-1	CAPZA1	64	41	82	172	58	210	57	35	10	32	24	21	56	36	1.208E-02	1.523E-01	6.241E-01	1.523E-02	2.580	0.662	3.896
Q8WZA1	Protein O-linked-mannose beta-1,2-N-acetylglucosaminyltransferase 1	POMGNT1	277	278	280	295	228	349	483	574	422	468	424	470	359	449	1.247E-02	1.028E-01	8.082E-01	1.873E-02	0.734	1.108	0.662
P62993	Growth factor receptor-bound protein 2	GRB2	131	91	148	316	241	284	250	32	18	139	95	46	59	97	1.260E-02	7.663E-02	9.221E-01	2.234E-02	3.102	1.057	2.934
P52566	Rho GDP-dissociation inhibitor 2	ARHGDIB	446	403	662	1818	829	1724	872	361	201	487	323	167	437	280	1.280E-02	3.238E-02	9.057E-01	4.755E-02	3.271	1.163	2.812
P10720	Platelet factor 4 variant	PF4V1	1396	2738	2390	1436	3064	1011	2786	448	788	529	1727	836	1178	629	1.297E-02	7.312E-02	9.428E-01	2.392E-02	2.404	0.991	2.425
P04004	Vitronectin	VTN	24959	27267	34972	25165	33114	24405	30303	14734	22781	19755	23010	23698	28801	32423	1.338E-02	9.968E-01	5.603E-02	1.794E-02	1.010	0.709	1.425
P60660	Myosin light polypeptide 6	MYL6	661	487	1550	1764	1018	3173	858	136	52	507	347	286	711	1510	1.338E-02	6.475E-01	1.557E-01	1.358E-02	1.626	0.312	5.211
P20160	Azurocidin	AZU1	170	252	129	232	286	148	441	142	138	80	102	92	134	105	1.420E-02	4.747E-02	9.921E-01	3.807E-02	2.149	1.049	2.048
P26641	Elongation factor 1-gamma	EEF1G	492	207	512	1044	728	684	963	52	11	432	251	50	115	154	1.496E-02	6.263E-02	9.946E-01	3.258E-02	6.202	1.748	3.549
O14950	Myosin regulatory light chain 12B	MYL12B	37	35	50	64	47	70	39	32	19	29	28	23	31	47	1.507E-02	1.648E-01	6.608E-01	1.910E-02	1.461	0.803	1.820
P00568	Adenylate kinase isoenzyme 1	AK1	347	476	534	622	372	864	577	297	189	207	161	209	913	370	1.529E-02	7.343E-01	1.416E-01	1.584E-02	1.089	0.429	2.537
P02766	Transthyretin	TTR	2758	3550	3632	3979	3152	2150	3539	3609	8816	4610	7038	4784	4062	4431	1.552E-02	2.493E-01	4.881E-01	1.726E-02	0.735	1.360	0.540
P68366	Tubulin alpha-4A chain	TUBA4A	519	352	1098	725	645	2857	527	142	64	369	260	222	338	615	1.603E-02	3.203E-01	3.972E-01	1.688E-02	2.452	0.533	4.603
Q96PD5	N-acetylmuramoyl-L-alanine amidase	PGLYRP2	3625	4242	4167	4843	4535	4156	3849	7107	4898	5553	5128	5226	4545	7471	1.607E-02	5.849E-02	9.999E-01	3.821E-02	0.731	0.987	0.741
P29350	Tyrosine-protein phosphatase non-receptor type 6	PTPN6	333	185	359	1096	372	1062	414	110	98	275	184	81	229	188	1.683E-02	6.026E-02	9.998E-01	3.993E-02	3.293	1.006	3.274
P05109	Protein S100-A8	S100A8	1808	1762	1835	9153	3146	5205	2424	1816	780	638	807	983	2169	464	1.755E-02	7.494E-02	9.873E-01	3.568E-02	3.002	0.838	3.583
O00194	Ras-related protein Rab-27B	RAB27B	545	213	406	397	640	513	978	3	5	318	176	12	47	176	1.834E-02	9.790E-02	9.334E-01	3.156E-02	6.742	1.606	4.198
O43157	Plexin-B1	PLXNB1	405	351	376	425	366	447	403	667	519	453	485	519	398	645	1.861E-02	8.599E-02	9.702E-01	3.507E-02	0.761	1.020	0.746
P06733	Alpha-enolase	ENO1	2089	1991	3887	8736	3150	9254	2768	2048	916	1388	1214	1125	2433	1794	1.885E-02	1.308E-01	8.353E-01	2.772E-02	2.553	0.780	3.273
P04745	Alpha-amylase 1	AMY1A	3998	7944	3473	1809	3134	1878	4001	2618	1308	1633	1618	1344	1844	1147	1.908E-02	3.769E-02	8.192E-01	8.159E-02	2.594	1.242	2.089
Q9UBW5	Bridging integrator 2	BIN2	206	161	647	173	379	261	492	3		258	137	6	27	82	1.916E-02	4.446E-02	9.071E-01	6.666E-02	8.654	3.464	2.498
Q9BYH1	Seizure 6-like protein	SEZ6L	27	30	31	20	25	21	27	31	33	39	38	27	22	33	2.038E-02	8.646E-01	1.287E-01	2.219E-02	0.939	1.291	0.727
O95810	Caveolae-associated protein 2	SDPR; CAVIN2	119	85	260	109	164	358	181	12	8	118	68	28	37	123	2.046E-02	1.810E-01	7.178E-01	2.629E-02	2.912	0.820	3.552
P61960	Ubiquitin-fold modifier 1	UFM1	193	164	212	371	273	306	302	126	91	204	155	86	180	175	2.068E-02	7.477E-02	9.992E-01	4.524E-02	1.769	0.979	1.807
Q92896	Golgi apparatus protein 1	GLG1	167	369	326	263	417	152	340	113	206	70	184	134	172	115	2.154E-02	8.873E-02	9.849E-01	4.198E-02	2.068	1.019	2.030
P04080	Cystatin-B	CSTB	299	501	767	183	459	188	198	208	135	93	81	117	166	226	2.183E-02	1.861E-01	7.262E-01	2.804E-02	2.184	0.762	2.867
O00299	Chloride intracellular channel protein 1	CLIC1	809	718	1418	1993	1045	4368	1076	438	128	688	460	254	806	788	2.192E-02	1.830E-01	7.351E-01	2.839E-02	2.650	0.695	3.811
P05067	Amyloid-beta precursor protein	APP	4409	6091	6694	4503	9819	3039	4900	2145	4082	1198	3591	2665	3396	1893	2.196E-02	8.531E-02	9.924E-01	4.441E-02	2.126	1.039	2.046
Q9H4M9	EH domain-containing protein 1	EHD1	139	122	121	233	147	144	167	48	76	117	126	83	102	107	2.254E-02	1.397E-01	8.611E-01	3.345E-02	1.575	0.942	1.672
Q13822	Ectonucleotide pyrophosphatase/phosphodiesterase family member 2	ENPP2	2459	2279	1751	2027	2245	2241	1961	4579	2306	3584	2781	2811	2064	3153	2.274E-02	3.370E-01	4.687E-01	2.424E-02	0.799	1.238	0.645
P08311	Cathepsin G	CTSG	83	160	96	962	231	103	162	77	64	47	42	43	89	38	2.360E-02	7.079E-02	9.936E-01	5.776E-02	4.533	1.012	4.478
Q6P179	Endoplasmic reticulum aminopeptidase 2	ERAP2	2565	3510	3175	3552	3757	4542	3401	1736	6490	3885	4004	2566	1176	1490	2.371E-02	3.689E-02	4.361E-02	9.740E-01	2.007	2.310	0.869
P31949	Protein S100-A11	S100A11	127	127	724	1016	225	301	213	136	98	85	85	76	146	74	2.418E-02	7.164E-02	9.929E-01	5.929E-02	3.964	1.025	3.866
O00151	PDZ and LIM domain protein 1	PDLIM1	427	175	478	592	654	1422	941	43	3	294	186	70	114	276	2.475E-02	2.506E-01	6.278E-01	2.892E-02	4.366	0.856	5.098
P06753	Tropomyosin alpha-3 chain	TPM3	402	315	518	602	619	583	568	176	245	393	347	293	336	527	2.534E-02	3.775E-01	4.496E-01	2.654E-02	1.337	0.754	1.774
P02776	Platelet factor 4	PF4	9014	13601	14247	8619	17579	4944	13763	3060	8549	3041	7342	4799	7158	4028	2.582E-02	9.866E-02	9.891E-01	4.996E-02	2.192	1.032	2.125
Q9BUN1	Protein MENT	C1orf56	44	41	56	202	32	61	42	103	286	175	287	187	47	38	2.596E-02	8.866E-01	1.447E-01	2.837E-02	0.754	2.347	0.321
Q9H299	SH3 domain-binding glutamic acid-rich-like protein 3	SH3BGRL3	104	53	80	452	135	291	160	28	13	103	70	16	47	53	2.630E-02	6.616E-02	9.641E-01	7.315E-02	4.735	1.388	3.411
P01019	Angiotensinogen	AGT	25204	29241	24922	25504	20284	28851	35427	44323	39644	30132	36482	35908	27054	38241	2.635E-02	2.199E-01	7.094E-01	3.262E-02	0.802	1.116	0.719
Q13418	Integrin-linked protein kinase	ILK	156	100	231	329	183	1688	169	56	11	91	73	52	113	142	2.690E-02	3.083E-01	5.569E-01	2.972E-02	3.984	0.564	7.061
P02775	Platelet basic protein	PPBP	8293	14474	14709	9929	18110	7530	12890	4684	9041	3998	11530	5840	7151	5036	2.802E-02	6.200E-02	9.245E-01	8.731E-02	2.043	1.217	1.679
Q5T2D2	Trem-like transcript 2 protein	TREML2	41	79	67	38	87	41	44	30	39	41	38	37	24	32	2.828E-02	4.419E-02	7.099E-01	1.418E-01	1.839	1.193	1.542
Q9Y6R7	IgGFc-binding protein	FCGBP	8220	5722	5051	4950	6189	12436	6650	14693	13028	13235	8219	13102	6863	11806	2.841E-02	1.928E-01	7.938E-01	3.773E-02	0.664	1.161	0.572
P28331	NADH-ubiquinone oxidoreductase 75 kDa subunit, mitochondrial	NDUFS1	60	66	62	51	70	50	70	71	84	75	72	84	63	82	2.852E-02	8.241E-02	9.947E-01	6.703E-02	0.797	0.984	0.811
Q86UX7	Fermitin family homolog 3	FERMT3	1501	943	1852	2445	1769	8122	2016	298	218	1549	896	417	767	1314	2.855E-02	1.674E-01	8.561E-01	4.073E-02	3.198	0.889	3.598
P09493	Tropomyosin alpha-1 chain	TPM1	1407	1195	2349	2729	2399	2882	2223	1040	1032	1424	1472	1344	1402	1741	2.864E-02	2.553E-01	6.657E-01	3.390E-02	1.450	0.831	1.746
P30086	Phosphatidylethanolamine-binding protein 1	PEBP1	512	680	868	1422	884	1798	923	726	266	368	285	307	1007	594	2.951E-02	3.409E-01	5.381E-01	3.199E-02	1.591	0.646	2.461
P35579	Myosin-9	MYH9	1528	1183	4130	3115	2253	8596	2094	688	407	1326	934	845	1794	3517	2.996E-02	6.408E-01	2.770E-01	3.000E-02	1.594	0.409	3.902
Q8IWV2	Contactin-4	CNTN4	1037	1116	812	930	1129	1125	1182	1562	1260	1590	1324	1511	929	1551	3.006E-02	2.136E-01	7.650E-01	3.850E-02	0.787	1.078	0.730
Q14766	Latent-transforming growth factor beta-binding protein 1	LTBP1	4391	5196	5516	3777	7574	3531	5862	2341	4136	1537	4027	2745	3878	1640	3.048E-02	7.533E-02	9.691E-01	8.111E-02	1.859	1.093	1.701
P09619	Platelet-derived growth factor receptor beta	PDGFRB	696	476	394	583	407	278	774	1461	1360	689	532	428	234	510	3.058E-02	6.331E-01	4.306E-02	8.762E-02	1.320	2.587	0.510
P12111	Collagen alpha-3(VI) chain	COL6A3	7553	8319	6236	9588	7751	7126	7203	14283	10395	11548	8484	9642	7376	13231	3.059E-02	2.217E-01	7.532E-01	3.864E-02	0.762	1.109	0.687
O75503	Ceroid-lipofuscinosis neuronal protein 5	CLN5	254	177	184	158	170	249	323	288	245	363	442	382	240	357	3.183E-02	1.097E-01	9.948E-01	6.176E-02	0.664	1.026	0.647
P55083	Microfibril-associated glycoprotein 4	MFAP4	46	36	36	27	72	8	72	109	73	84	75	94	84	118	3.219E-02	7.231E-02	9.433E-01	9.268E-02	0.429	0.863	0.497
P80511	Protein S100-A12	S100A12	242	266	187	3366	558	1222	512	266	90	68	120	147	304	57	3.256E-02	1.247E-01	9.783E-01	5.790E-02	5.363	0.803	6.676
Q9Y490	Talin-1	TLN1	6902	3003	8145	7071	6656	24187	7671	883	451	5962	3510	1238	2439	5138	3.295E-02	1.950E-01	8.342E-01	4.487E-02	3.094	0.919	3.365
P28906	Hematopoietic progenitor cell antigen CD34	CD34	33	37	47	57	39	26	44	37	159	77	151	103	67	40	3.354E-02	2.991E-01	6.396E-01	3.828E-02	0.574	1.512	0.380
P08567	Pleckstrin	PLEK	233	101	146	186	160	556	308	24	21	197	131	29	50	115	3.410E-02	9.107E-02	9.906E-01	8.017E-02	3.715	1.437	2.585
P07711	Cathepsin L1	CTSL	189	225	224	204	250	160	202	237	274	255	244	232	250	374	3.447E-02	5.166E-02	7.086E-01	1.668E-01	0.729	0.885	0.824
Q15185	Prostaglandin E synthase 3	PTGES3	72	70	172	242	130	262	186	86	46	85	55	34	108	69	3.498E-02	9.746E-02	9.963E-01	7.825E-02	2.303	0.963	2.391
P07359	Platelet glycoprotein Ib alpha chain	GP1BA	2324	2276	2527	2412	3243	1241	3414	1440	1772	1615	1787	1819	1283	1379	3.507E-02	6.500E-02	8.624E-01	1.223E-01	1.668	1.107	1.506
Q15762	CD226 antigen	CD226	216	312	742	138	372	92	319	101	142	119	126	80	72	149	3.514E-02	6.334E-02	8.459E-01	1.272E-01	3.114	1.213	2.567
**P41218**	**Myeloid cell nuclear differentiation antigen**	**MNDA**	**60**	**40**	**70**	**953**	**94**	**311**	**89**	**41**	**4**	**27**	**35**	**34**	**53**	**32**	**3.577E-02**	**2.819E-01**	**6.889E-01**	**4.203E-02**	**5.817**	**0.668**	**8.715**
P0DP25	Calmodulin-3	CALM3	250	279	550	574	463	773	562	122	56	387	271	104	305	250	3.633E-02	1.717E-01	9.111E-01	5.427E-02	2.245	0.952	2.358
P37840	Alpha-synuclein	SNCA	733	557	928	1628	654	1094	1253	179	290	537	449	196	1312	358	3.639E-02	2.316E-01	7.878E-01	4.651E-02	1.572	0.585	2.689
P49767	Vascular endothelial growth factor C	VEGFC	143	201	249	114	232	116	226	92	136	66	145	96	133	72	3.684E-02	8.444E-02	9.611E-01	9.767E-02	1.825	1.094	1.668
P22891	Vitamin K-dependent protein Z	PROZ	2715	9166	2632	6375	2310	5281	2297	6392	6778	6990	6406	7767	7111	11725	3.698E-02	5.607E-02	7.302E-01	1.688E-01	0.496	0.749	0.662
P60842	Eukaryotic initiation factor 4A-I	EIF4A1	27	24	105	116	30	149	36	17	1	18	15	11	43	23	3.714E-02	4.878E-01	4.373E-01	3.786E-02	2.703	0.495	5.462
P07437	Tubulin beta chain	TUBB	412	360	1026	873	585	2160	659	253	129	410	345	255	534	558	3.722E-02	3.958E-01	5.358E-01	3.953E-02	1.931	0.633	3.053
P13693	Translationally-controlled tumor protein	TPT1	492	442	857	1245	766	1487	916	350	51	612	401	152	421	333	3.734E-02	1.273E-01	9.910E-01	6.880E-02	2.937	1.172	2.507
Q86U17	Serpin A11	SERPINA11	1310	973	1486	490	853	900	900	448	745	514	537	740	971	1107	3.767E-02	9.980E-01	1.191E-01	4.805E-02	1.051	0.597	1.761
P01137	Transforming growth factor beta-1 proprotein	TGFB1	1258	1389	1591	1551	2204	1181	1797	931	1383	432	1224	869	1189	475	3.854E-02	7.300E-02	8.865E-01	1.248E-01	1.856	1.175	1.580
Q92859	Neogenin	NEO1	786	1065	824	1067	1091	1123	1115	1737	1276	1600	1259	1485	831	1561	3.886E-02	3.402E-01	6.230E-01	4.322E-02	0.782	1.136	0.688
P07339	Cathepsin D	CTSD	3174	3908	4205	2855	3956	4827	3496	2398	2426	2991	3044	2594	2743	3998	3.913E-02	3.093E-01	6.721E-01	4.496E-02	1.213	0.872	1.390
Q16610	Extracellular matrix protein 1	ECM1	5227	10287	8224	4574	9855	3878	3922	8704	15043	11213	10551	11275	7562	10090	3.986E-02	2.201E-01	8.382E-01	5.313E-02	0.681	1.180	0.577
Q00013	55 kDa erythrocyte membrane protein	MPP1	186	116	192	546	200	535	289	112	57	81	68	45	366	211	3.994E-02	5.264E-01	4.212E-01	4.040E-02	1.422	0.383	3.716
P07951	Tropomyosin beta chain	TPM2	207	183	444	332	351	1171	377	37	45	295	176	82	106	316	4.020E-02	2.339E-01	8.139E-01	5.210E-02	2.605	0.822	3.169
P67936	Tropomyosin alpha-4 chain	TPM4	1811	1193	2314	2679	3112	6139	3787	198	82	2313	1353	416	722	2020	4.043E-02	2.723E-01	7.447E-01	4.901E-02	2.854	0.937	3.046
P61224	Ras-related protein Rap-1b	RAP1B	364	182	329	510	279	558	345	62	57	328	209	91	153	295	4.090E-02	2.111E-01	8.633E-01	5.601E-02	2.043	0.915	2.233
P08473	Neprilysin	MME	122	123	117	172	132	280	305	103	125	95	93	89	106	102	4.120E-02	1.011E-01	9.847E-01	9.697E-02	1.801	1.047	1.720
O94769	Extracellular matrix protein 2	ECM2	518	410	449	607	353	486	429	613	505	582	628	530	538	655	4.338E-02	1.410E-01	9.917E-01	7.828E-02	0.809	1.013	0.798
Q14112	Nidogen-2	NID2	979	998	1072	1081	1869	1149	934	1072	801	365	830	619	900	431	4.429E-02	7.847E-02	8.673E-01	1.460E-01	1.776	1.180	1.506
O00533	Neural cell adhesion molecule L1-like protein	CHL1	4232	4475	4080	3239	4467	4109	4676	5218	4940	6060	5191	6707	3782	6241	4.628E-02	1.174E-01	9.943E-01	1.004E-01	0.750	0.960	0.781
P07737	Profilin-1	PFN1	2548	1376	3023	8531	4141	12783	4890	1057	71	2929	1754	470	1170	1361	4.665E-02	1.559E-01	9.846E-01	8.022E-02	5.326	1.452	3.667
Q9NY97	N-acetyllactosaminide beta-1,3-N-acetylglucosaminyltransferase 2	B3GNT2	1468	1739	1705	1352	1720	1287	2004	2084	1978	2159	2037	1763	1490	2195	4.670E-02	5.178E-01	4.699E-01	4.757E-02	0.887	1.137	0.780
P48740	Mannan-binding lectin serine protease 1	MASP1	3963	4986	4842	6608	4055	6192	5198	6964	6782	6673	6149	6549	5033	6125	4.774E-02	4.024E-01	6.026E-01	5.146E-02	0.868	1.125	0.771
P48357	Leptin receptor	LEPR	120	132	151	88	69	84	142	171	177	170	174	142	98	194	4.795E-02	4.173E-01	5.854E-01	5.119E-02	0.776	1.195	0.649
P51858	Hepatoma-derived growth factor	HDGF	126	151	204	261	194	267	131	122	88	135	131	104	166	119	4.817E-02	1.951E-01	9.342E-01	7.187E-02	1.470	0.920	1.598
Q15691	Microtubule-associated protein RP/EB family member 1	MAPRE1	398	252	547	952	597	1342	706	144		432	294	78	264	228	4.822E-02	2.080E-01	9.127E-01	6.924E-02	3.602	1.524	2.364
Q04917	14-3-3 protein eta	YWHAH	160	113	276	474	191	768	168	111	18	121	106	86	120	191	4.839E-02	4.127E-01	5.938E-01	5.186E-02	2.325	0.672	3.462
P05121	Plasminogen activator inhibitor 1	SERPINE1	249	452	587	527	500	651	440	463	259	123	293	264	356	199	4.864E-02	1.516E-01	9.929E-01	8.674E-02	1.781	1.042	1.710
Q05315	Galectin-10	CLC	147	145	138	906	214	110	133	86	137	64	76	57	115	47	4.955E-02	8.453E-02	8.602E-01	1.622E-01	3.511	1.242	2.828

Supplementary Table S4. Summary of the ingenuity pathway analysis (IPA) for the 90 proteins with statistically significant changes between psoriasis groups with and without CVD risk factors

† *P* -value is displayed in E notation: aEb indicates a value of a × 10^b.

‡ Numbers of molecules involved.

* The score is derived from a *P* -value and indicates the likelihood of the mapped genes in a network being found together due to random chance (score= $-\log_{10}P$)

**Top canonical pathways**	*P* -value†	Ratio
Integrin signaling	2.55E-07	9/205 (0.044)
Remodeling of epithelial adherens junctions	4.02E-07	6/66 (0.091)
GP6 signaling pathway	1.10E-06	7/124 (0.056)
Axonal guidance signaling	8.73E-06	11/492 (0.022)
Actin cytoskeleton signaling	9.41E-06	8/240 (0.033)
**Molecular and cellular functions**	*P* -value†	No.‡
Cell to cell signaling and interaction	1.92E-03 – 9.21E-17	43
Cellular movement	1.86E-03 – 1.82E-16	47
Cellular assembly and organization	1.86E-03 – 4.83E-10	39
Cellular function and maintenance	1.64E-03 – 4.83E-10	44
Cellular morphology	1.92E-03 - 1.50E-09	32
**Associated network functions**		Score* (No.‡)
Cellular assembly and organization, cellular compromise, cellular function and maintenance		39 (19)
Cell to cell signaling and interaction, hematological system development and function, inflammatory response		36 (18)
Cardiovascular system development and function, embryonic development, organ development		36 (18)

Supplementary Table S5. ELISA results of CALD1, LASP1, MNDA, and ZYX

[a] CVD risk_O represents concentration values of each biomarker candidate protein from psoriasis group with CVD risk factors.

[b] CVD risk_X represents concentration values of each biomarker candidate protein from psoriasis group without CVD risk factors.

[c] Mean concentration values of each biomarker candidate protein from psoriasis group with CVD risk factors.

[d] Mean concentration values of each biomarker candidate protein from psoriasis group without CVD risk factors.

[e] *P*-values are obtained from independent t-test or Mann-Whitney U-test comparing concentration values from psoriasis groups with and without CVD risk factors.

Samples	CALD1	LASP1	MNDA	ZYX
**CVD risk_O_1[a] [ng/mL]**	5.481	454.139	1.786	65.492
**CVD risk_O_2[a] [ng/mL]**	4.989	691.440	1.403	122.543
**CVD risk_O_3[a] [ng/mL]**	4.684	562.791	2.311	60.579
**CVD risk_O_4[a] [ng/mL]**	5.971	1082.956	1.645	188.350
**CVD risk_O_5[a] [ng/mL]**	4.751	490.820	0.909	100.924
**CVD risk_O_6[a] [ng/mL]**	2.736	321.938	1.171	99.267
**CVD risk_O_7[a] [ng/mL]**	1.149	574.794	0.945	127.554
**CVD risk_O_8[a] [ng/mL]**	4.220	211.638	0.976	25.552
**CVD risk_O_9[a] [ng/mL]**	4.712	581.515	1.121	50.772
**CVD risk_O_10[a] [ng/mL]**	5.529	778.067	1.086	49.956
**CVD risk_O_11[a] [ng/mL]**	8.928	1050.838	1.328	54.855
**CVD risk_O_12[a] [ng/mL]**	4.278	829.164	1.337	67.132
**CVD risk_O_13[a] [ng/mL]**	3.707	416.412	3.018	44.249
**CVD risk_O_14[a] [ng/mL]**	2.704	475.994	1.656	83.572
**CVD risk_O_15[a] [ng/mL]**	7.645	217.931	1.147	75.341
**CVD risk_O_16[a] [ng/mL]**	4.210	383.442	0.996	110.052
**CVD risk_O_17[a] [ng/mL]**	5.293	628.246	1.403	146.841
**CVD risk_O_18[a] [ng/mL]**	6.891	687.031	1.258	140.118
**CVD risk_O_19[a] [ng/mL]**	3.065	442.196	0.921	57.307
**CVD risk_O_20[a] [ng/mL]**	3.002	239.981	0.910	103.411
**CVD risk_O_21[a] [ng/mL]**	2.606	325.067	1.050	31.234
**CVD risk_O_22[a] [ng/mL]**	3.577	104.791	0.732	50.772
**CVD risk_X_1[b] [ng/mL]**	2.617	67.214	0.638	56.489
**CVD risk_X_2[b] [ng/mL]**	4.472	305.445	0.713	99.267
**CVD risk_X_3[b] [ng/mL]**	5.689	734.825	1.161	100.096
**CVD risk_X_4[b] [ng/mL]**	2.617	378.984	1.085	32.859
**CVD risk_X_5[b] [ng/mL]**	3.293	462.584	0.693	56.489
**CVD risk_X_6[b] [ng/mL]**	1.467	643.753	1.068	70.413
**CVD risk_X_7[b] [ng/mL]**	5.349	611.547	6.616	59.760
**CVD risk_X_8[b] [ng/mL]**	2.573	507.116	0.906	81.100
**CVD risk_X_9[b] [ng/mL]**	3.727	188.812	0.653	66.312
**CVD risk_X_10[b] [ng/mL]**	4.684	179.414	0.972	25.552
**CVD risk_X_11[b] [ng/mL]**	5.160	353.769	1.334	46.694
**CVD risk_X_12[b] [ng/mL]**	2.051	762.469	1.845	29.610
**CVD risk_X_13[b] [ng/mL]**	3.231	314.681	0.928	30.422
**CVD risk_X_14[b] [ng/mL]**	2.928	403.771	0.732	47.509
**CVD risk_X_15[b] [ng/mL]**	2.328	655.152	1.200	61.397
**CVD risk_X_16[b] [ng/mL]**	0.774	375.655	0.745	33.671
**CVD risk_X_17[b] [ng/mL]**	4.385	152.154	0.783	32.046
**CVD risk_X_18[b] [ng/mL]**	0.937	327.160	0.755	90.997
**CVD risk_X_19[b] [ng/mL]**	1.823	240.916	0.761	101.753
**CVD risk_X_20[b] [ng/mL]**	6.093	339.829	1.252	105.070
**Mean concentration values**				
CVD risk_O (ng/mL, mean±SD)[c]	4.551±1.793	525.054±257.726	1.323±0.523	84.358±41.920
CVD risk_X (ng/mL, mean±SD)[d]	3.310±1.580	400.262±198.312	1.242±1.299	61.375±27.142
P-value[e]	0.0313	0.1126	0.0131	0.0435

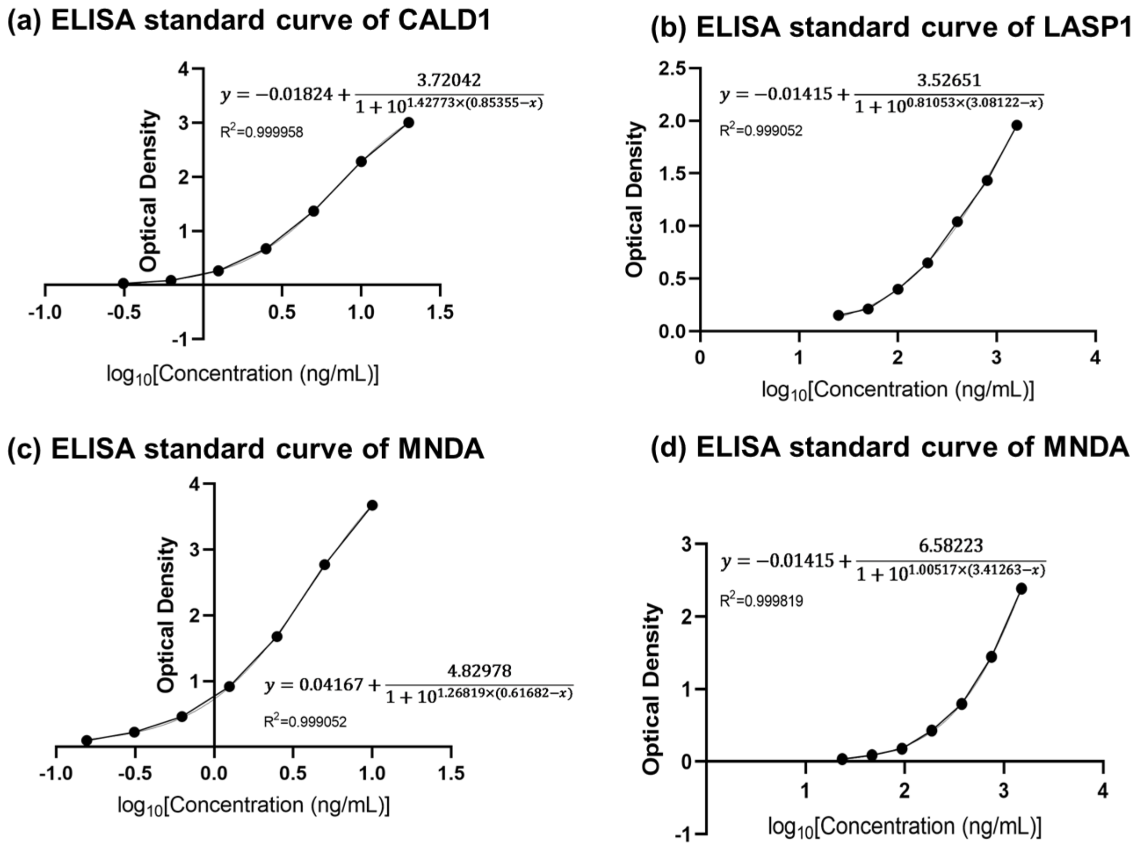

Supplementary Figure S1. Standard curves of each biomarker candidates were obtained using the four-parameter logistic (4PL) model. R^2 value of each standard curves are shown below the equation. (a) CALD1: Caldesmon, (b) LASP1: LIM and SH3 domain protein, (c) MNDA: Myeloid cell nuclear differentiation antigen, (d) ZYX: Zyxin

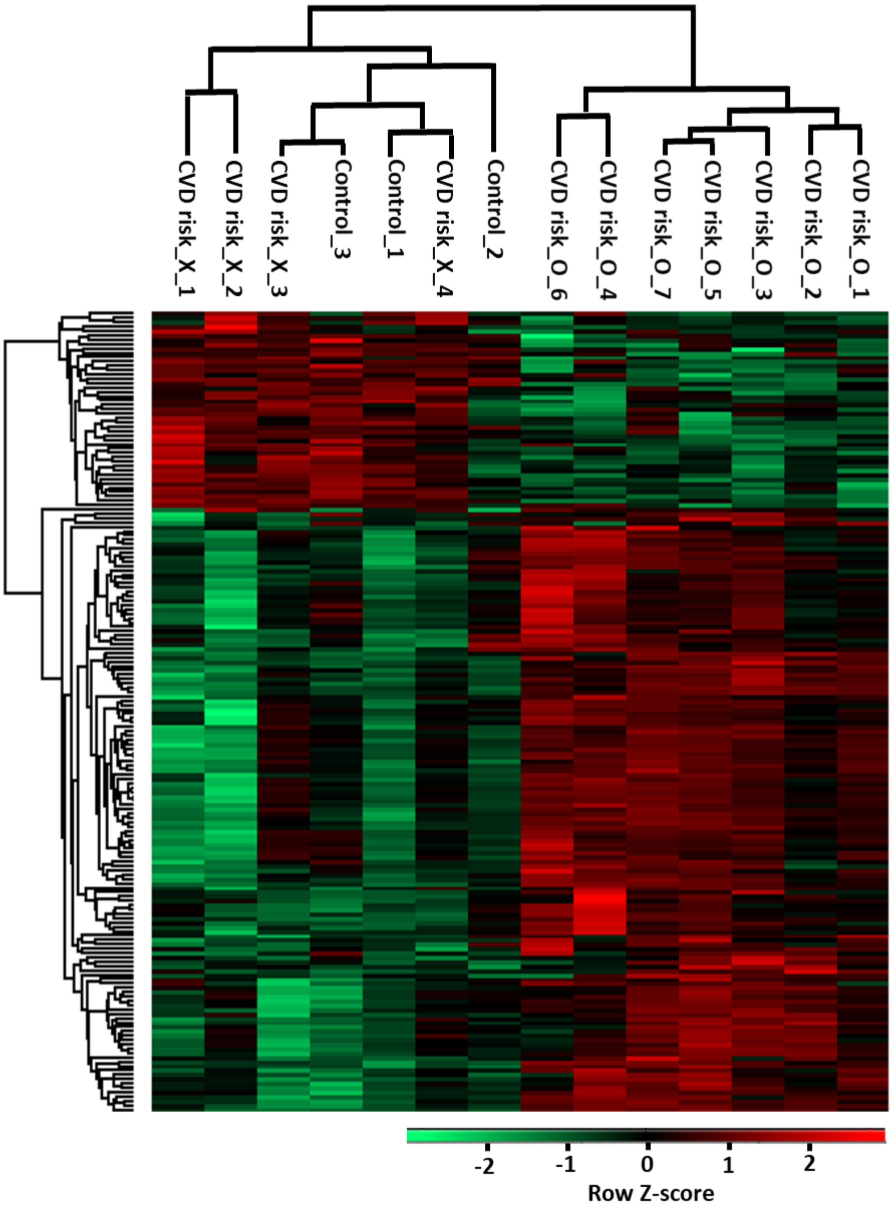

Supplementary Figure S2. Heat map exhibiting hierarchical clustering of 184 proteins with statistical significance (*P*-value <0.05) from ANOVA among the psoriasis groups with and without cardio vascular disease (CVD) risk factors and the healthy group. The rows represent each protein and the columns show four and seven biological replicates of psoriasis serum samples without and with CVD risk factors, and three biological replicates of the healthy group. Hierarchical clustering of the 184 proteins was performed in Perseus software (1.6.14.0) on log-transformed normalized signal-to-noise ratio values after z-score normalization of the data.
